# Supplementary material for: Sports Stars Brazil in children with autism spectrum disorder: A feasibility randomized controlled trial protocol
Source: PLoS One. 2023 Nov 8;18(11):e0291488. doi: 10.1371/journal.pone.0291488 (PMC10631688; doi:10.1371/journal.pone.0291488)
Supplement: S1 File — (DOCX) [file pone.0291488.s003.docx]

**Supporting Information - S3**

**Satisfaction questionnaire for the Sports Stars group.**

| Satisfaction of children | | Did not like | | I liked it a little | Really enjoyed |
| --- | --- | --- | --- | --- | --- |
| What is your level of satisfaction about | | |  | |  |
|  | Intervention time (10:00am to 11:00am). |  |  | |  |
|  | The duration of the intervention (1 hour per session). |  |  | |  |
|  | The period of the intervention (8 weeks).  The total amount of intervention (8 hours) |  |  | |  |
|  | The place where the interventions took place (for example: the location of the court, the size of the court, the cleanliness of the bathrooms, among others). |  |  | |  |
|  | The materials used during the activities (for example: balls, cones, disks, baskets, among others). |  |  | |  |
|  | The practice of modified sports (for example: the activities developed; the interaction and participation between your child, the other participants and the therapists, the objectives of the activities, the formation of teams, among others). |  |  | |  |
|  | The practice of activities to be done with other children (for example: doing the relay races, the activities in pairs of throwing and receiving a ball, and during the practice of the modified sport, among others). |  |  | |  |
|  | The therapists' interaction with you and your child (for example: the way they instruct you about the activities; the way they talk to your child before, during and after the interventions; among others). |  |  | |  |
|  | The way in which the therapist adapted the Sports Stars activities according to what your child was able to do (for example: when the therapist made the activity difficult if it was easy, or when he facilitated the activity when it was difficult, among others). |  |  | |  |
|  | The interaction between your child and the other children (for example: the communication between them and the other children during the intervention, the support between them during the activities). |  |  | |  |
|  | Your child's current skill level (in soccer, handball, basketball and athletics) when compared to before the intervention (for example: how far they can bounce a ball today compared to before the project, among others). |  |  | |  |

**S3. Table**. Adapted from Feitosa et al.^39^
