# Supplementary material for: Sports Stars Brazil in children with autism spectrum disorder: A feasibility randomized controlled trial protocol
Source: PLoS One. 2023 Nov 8;18(11):e0291488. doi: 10.1371/journal.pone.0291488 (PMC10631688; doi:10.1371/journal.pone.0291488)
Supplement: S2 File — (DOCX) [file pone.0291488.s004.docx]

Supporting Information - S4 (Sports Stars Session Samples)

| **Sports Stars Brazil- Session Plan**  **Handball**  Child’s name: ______________________ Therapist: ______________________  Week #_____ Date: ____/____/____ | | | | | | |
| --- | --- | --- | --- | --- | --- | --- |
| **Warm Up** | | **Involvement** | | **1 2 3 4** | | |
| ***Range of Motion***  ***Upper extremities and trunk***   - Rolling shoulders - Circle arms | | ***Lower Extremities and trunk***   - Standing toe touch - Stork stand (quad stretch) - Ankle circles | | | ***Increase Heart Rate***   - High knees - Jump up down, left right - Star jumps | |
| **Locomotor Activities** | | **Involvement** | | | **1 2 3 4** | |
| **Activity** | **Physical** | **Cognitive** | **Activity** | | | **Physical** |
| **Relay Race** | **+2** Runs and pass the baton quickly with coordination | +2 Analyses alone the technique in order to increase speed when passing the baton | +2 Competitive and encouraging | | | +2 Confident/ motivated to improve challenging parts of activity |
|  | **+1** Runs and pass the baton quickly, but without coordination | +1 Applies feedback from the therapist to increase speed when passing the baton | +1 Developing positive team connections | | | +1 Confident/ motivated to attempt challenging parts of activity |
|  | **0** Runs and pass the baton slowly and without coordination | 0 Understands when to pass the baton | 0 Interacts positively | | | 0 Confident to try most parts of activity |
|  | **-1** Initiates running and coordination to pass the baton | -1 Remembers the rule to pass the baton | -1 Neutral interaction | | | -1 Needs encouragement to try some parts of activity |
|  | **-2** Walks fast to pass the baton, has to stop to pass with coordination | -2 Learning about passing the baton | -2 Inappropriate peer interactions | | | **-**2 Needs encouragement to try any part of activity |
| **Run weaving pylons** | **+2** Runs quickly between pylons (short distance) without touching or bumping them | +2 Analyses alone the technique in order to increase speed when weaving the pylons | +2 Competitive and encouraging | | | +2 Confident/ motivated to improve challenging parts of activity |
|  | **+1** Runs slowly between pylons (short distance) without touching or bumping them | +1 Applies feedback from the therapist to increase speed when weaving the pylons | +1 Developing positive team connections | | | +1 Confident/ motivated to attempt challenging parts of activity |
|  | **0** Runs slowly between pylons (long distance) without touching or bumping them | 0 Understands when to weave the pylons | 0 Interacts positively | | | 0 Confident to try most parts of activity |
|  | **-1** Runs slowly between pylons (long distance) may touch or bump them | -1 Remembers the rule to weave the pylons without bumping or touching | -1 Neutral interaction | | | -1 Needs encouragement to try some parts of activity |
|  | **-2** Walks fast between pylons (long distance) may touch or bump them | -2 Learning about weaving the pylons | -2 Inappropriate peer interactions | | | **-**2 Needs encouragement to try any part of activity |
| **Lateral Running** | **+2** Runs in lateral without turning body or head to orientate, returns running laterally | +2 Analyses alone the technique in order to run in lateral without turning the body or head | +2 Competitive and encouraging | | | +2 Confident/ motivated to improve challenging parts of activity |
|  | **+1** Runs in lateral turning body or head to orientate, returns running laterally | +1 Applies feedback from the therapist to run in lateral without turning the body or head | +1 Developing positive team connections | | | +1 Confident/ motivated to attempt challenging parts of activity |
|  | **0** Initiates lateral run turning body or head to orientate, unable to complete the sequence | 0 Understands when to run in lateral | 0 Interacts positively | | | 0 Confident to try most parts of activity |
|  | **-1** Walks fast in lateral without turning body or head to orientate, returns walking laterally | -1 Remembers the rule to run in lateral without turning the body or head | -1 Neutral interaction | | | -1 Needs encouragement to try some parts of activity |
|  | **-2** Walks fast in lateral turning body or head to orientate, unable to complete the sequence | -2 Learning about running lateral without turning the body or head | -2 Inappropriate peer interactions | | | **-**2 Needs encouragement to try any part of activity |
| **Running backwards** | **+2** Run backwards fast without turning body or head to orientate | +2 Analyses alone the technique in order to run backwards without turning the body or head | +2 Competitive and encouraging | | | +2 Confident/ motivated to improve challenging parts of activity |
|  | **+1** Run backwards without turning body or head to orientate | +1 Applies feedback from the therapist to run backwards without turning the body or head | +1 Developing positive team connections | | | +1 Confident/ motivated to attempt challenging parts of activity |
|  | **0** Initiates running backwards turning body or head to orientate | 0 Understands when run backwards | 0 Interacts positively | | | 0 Confident to try most parts of activity |
|  | **-1** Walks backwards fast without turning body or head to orientate | -1 Remembers the rule to run backwards without turning the body or head | -1 Neutral interaction | | | -1 Needs encouragement to try some parts of activity |
|  | **-2** Walks backwards fast turning body or head to orientate | -2 Learning about running backwards without turning the body or head | -2 Inappropriate peer interactions | | | **-**2 Needs encouragement to try any part of activity |
| **Object Control Activities** | | **Involvement** | | **1 2 3 4** | | |
| **Activity** | **Physical** | **Cognitive** | **Activity** | | | **Physical** |
| **Goal Throwing** | **+2** Throws a ball (overhand), goal 5m away | +2 Analyses alone the technique in order to throw the ball in the goal | +2 Competitive and encouraging | | | +2 Confident/ motivated to improve challenging parts of activity |
|  | **+1** Throws a ball (overhand), goal 3m away | +1 Applies feedback from the therapist to throw the ball in the goal | +1 Developing positive team connections | | | +1 Confident/ motivated to attempt challenging parts of activity |
|  | **0** Throws a ball (overhand), goal 1.5m away | 0 Understands when to throw the ball in the goal | 0 Interacts positively | | | 0 Confident to try most parts of activity |
|  | **-1** Throws a ball (underhand), goal 5m away | -1 Remembers the rule to throw the ball in the goal | -1 Neutral interaction | | | -1 Needs encouragement to try some parts of activity |
|  | **-2** Throws a ball (underhand), goal 3m away | -2 Learning about throwing the ball in the goal | -2 Inappropriate peer interactions | | | **-**2 Needs encouragement to try any part of activity |
| **Ball Bouncing** | **+2** Runs and bounces a ball ~3m in diagonal | +2 Analyses alone the technique in order to run and bounce changing direction | +2 Competitive and encouraging | | | +2 Confident/ motivated to improve challenging parts of activity |
|  | **+1** Runs and bounces a ball ~3m straight | +1 Applies feedback from the therapist to run and bounce changing direction | +1 Developing positive team connections | | | +1 Confident/ motivated to attempt challenging parts of activity |
|  | **0** Walks and bounces a ball ~3m in diagonal | 0 Understands when to run and bounce | 0 Interacts positively | | | 0 Confident to try most parts of activity |
|  | **-1** Walks and bounces a ball ~3m straight | -1 Remembers the rule to run and bounce | -1 Neutral interaction | | | -1 Needs encouragement to try some parts of activity |
|  | **-2** Walks and bounces a ball ~1.5m straight | -2 Learning about running and bouncing | -2 Inappropriate peer interactions | | | **-**2 Needs encouragement to try any part of activity |
| **Goal Protection- Catching the ball** | **+2** Catches a small ball with both hands, fast throw | +2 Analyses alone the technique in order to catch the ball with both hands | +2 Competitive and encouraging | | | +2 Confident/ motivated to improve challenging parts of activity |
|  | +1 Hits a small ball with any part of the body, fast throw | +1 Applies feedback from the therapist to catch the ball with both hands | +1 Developing positive team connections | | | +1 Confident/ motivated to attempt challenging parts of activity |
|  | **0** Catches a small ball with both hands, slow throw | 0 Understands when to catch the ball | 0 Interacts positively | | | 0 Confident to try most parts of activity |
|  | **-1** Catches a big ball with both hands, slow throw | -1 Remembers the rule to catch the ball with both hands | -1 Neutral interaction | | | -1 Needs encouragement to try some parts of activity |
|  | **-2** Hits a big ball with any part of the body, slow throw | -2 Learning about catching the ball | -2 Inappropriate peer interactions | | | **-**2 Needs encouragement to try any part of activity |
| **Modified Sports** | | **Involvement** | | **1 2 3 4** | | |
| **Activity** | **Physical** | **Cognitive** | **Social** | | | **Psychological** |
| **Handball** | **+2** Different teams, goal protection included | 2 Analyses alone the technique in order to develop tactics for a better performance | 2 Competitive and encouraging | | | 2 Confident/ motivated to improve challenging parts of the sport |
|  | **+1** Different teams, goal protection not included | 1 Applies feedback from the therapist to develop tactics for a better performance | 1 Developing positive team connections | | | 1 Confident/ motivated to attempt challenging parts of the sport |
|  | **0** Same teams, goal protection included | 0 Understands how to participate in the sport | 0 Interacts positively | | | 0 Confident to try most parts of the sport |
|  | **-1** Same teams, goal protection not included | -1 Remembers the sport’s rules | -1 Neutral interaction | | | -1 Needs encouragement to try some parts of the sport |
|  | **-2** Same team, passing the ball between teammates | -2 Learning about the sport’s rules | -2 Inappropriate peer interactions | | | **-**2 Needs encouragement to try any part of the sport |
| **Cool Down** | | **Involvement** | | **1 2 3 4** | | |
| **"Animal Yoga” or “Simon Says”: dynamic stretching, weight bearing and balance**   - Downward dog (Hamstring and calf stretch) - Happy cat, angry cat (trunk ROM) - Flamingo (SLS) - Giraffe (side lunge adductor & lateral trunk stretch) - Butterfly (short adductor stretch) | | | | | | |
| **Notes** | | | | | | |

**S4. Table.**
