# Supplementary material for: Sports Stars Brazil in children with autism spectrum disorder: A feasibility randomized controlled trial protocol
Source: PLoS One. 2023 Nov 8;18(11):e0291488. doi: 10.1371/journal.pone.0291488 (PMC10631688; doi:10.1371/journal.pone.0291488)
Supplement: S3 File — (DOCX) [file pone.0291488.s005.docx]

**Daily activity – S5**

| **SPORTS STARS Brazil** | | | |  |
| --- | --- | --- | --- | --- |
| **DAILY ACTIVITY LOG** | | | |  |
| Full name: | | | |  |
| Responsible Name: | | | |  |
| Weeks | Did you have any therapy? | Which one? | How many times? |  |
| 1 |  |  |  |  |
| 2 |  |  |  |  |
| 3 |  |  |  |  |
| 4 |  |  |  |  |
| 5 |  |  |  |  |
| 6 |  |  |  |  |
| 7 |  |  |  |  |
| 8 |  |  |  |  |

**S5. Table.**
