# Supplementary material for: Sports Stars Brazil in children with autism spectrum disorder: A feasibility randomized controlled trial protocol
Source: PLoS One. 2023 Nov 8;18(11):e0291488. doi: 10.1371/journal.pone.0291488 (PMC10631688; doi:10.1371/journal.pone.0291488)
Supplement: S4 File — (PDF) [file pone.0291488.s006.pdf]

EFFECTIVENESS OF SPORTS STARS BRASIL ON OUTCOMES OF ACTIVITY, PARTICIPATION AND PHYSICAL LITERACY IN CHILDREN AND ADOLESCENTS WITH AUTISM SPECTRUM DISORDER: A RANDOMIZED CONTROLLED TRIAL

Responsible teacher:

Prof. doctor Hercules Ribeiro Leite

Belo Horizonte
2022

SUMMARY

The Austic Spectrum Disorder (ASD) is a neurodevelopmental disorder characterized by difficulties in communication, social interaction and repetitive and/or restricted behaviors and/or interests. Children and adolescents with ASD may have impairments in generalized motor development, such as difficulties with fine/gross motor coordination and balance. In addition, this public is at risk of physical inactivity, obesity and overweight. Physical activities and sports are necessary to increase the participation of these individuals, and one way to achieve this is to promote interventions that develop "physical literacy". In this context, sport-focused interventions prepare the child or adolescent for the transition from individually performed interventions to participation in sports and recreational activities in the community, and with that in mind, Sports Stars would be a bridge to promote this transition. Despite the benefits reported in the literature of Sports Stars for children with cerebral palsy, the effects of Sports Stars on the population with ASD are still unknown. In this perspective, the objective of this study is to verify the effectiveness of the Sports Stars Brasil intervention program in children and adolescents with ASD in improving activity outcomes (mobility and activities of daily living), participation (frequency, involvement and responsibility) and physical literacy (physical, social, cognitive and psychological) compared to the control group (conventional therapy/waiting list). It is also an objective to explore the experiences of the participants and their families who participated in the Sports Stars program and to identify the results perceived by them regarding the domains of physical literacy. This study will be a randomized controlled trial involving approximately 38 children aged 6 to 11 years, 11 months and 29 days. A feasibility study will also be conducted involving 18 adolescents between 12 and 18 years old. The sample will be recruited for convenience through disclosure on social networks by Instagram of the Sports Stars Brasil extension project. Participants will be eligible if they are between 6 and 18 years old at the start of the intervention, diagnosed with ASD, classified as mild to moderate on the Childhood Autism Rating Scale (CARS), and at levels I and II on the Autism Classification System of Functioning: Social Communication (ACSF: SC). Participants will be excluded if they have cognitive, behavioral or clinical limitations (cardio-respiratory diseases) that prevent them from following instructions and safely participating in the Sports

Stars program. The measurement instruments that will be used to evaluate the outcomes and characterize the sample will be: CARS, ACSF: SC, Abbreviated Sensory Profile 2, Participation and Environment Measure for children and young people (PEM-CY), Goal Attainment Scaling (GAS), Gross Motor Development Test-2 (TGMD-2), Ignite Challenge, Physical Literacy Questionnaire and Pediatric Evaluation of Disability Inventory - computer adaptive test (PEDI-CAT+ASD). The Gross Motor Development Test-2 (TGMD-2), Ignite Challenge, Muscle Power Sprint Test (MPST) and 10×5 Sprint Test (10×5ST) instruments will have their reliability evaluated for the population of children with ASD. Sports Stars is expected to be effective in improving the outcomes reported above for the ASD population, as well as being proven to be positive for children and adolescents with Cerebral Palsy, by providing improvement in the domain of physical literacy, such as locomotion skills and manipulation, participation in sport and recreational activities compared to the usual intervention group.

INTRODUCTION

Autism Spectrum Disorder (ASD) is a neurodevelopmental disorder characterized by difficulties in communication, social interaction and repetitive and/or restricted behaviors and/or interests (APA, 2014; WHO, 1992) and these symptoms constitute the core of the disorder, but the severity of its presentation is variable, being caused by a combination of genetic factors and environmental factors (SBG, 2019). According to the Developmental Disabilities and Autism Monitoring Network (ADDM), ASD prevalence estimates are increasing compared to previous ones. Studies that monitored the prevalence of ASD point to considerable variability among different populations, with a rate of 18.5 per 1,000, that is, 1 in 54 children have ASD (MAENNER et al., 2020; CARLON et al., 2013).

Children with ASDgenerallyexhibit atypical sensory processing, such as perceptual distortions, hypo- and hyper-responses, and concerns about the sensory characteristics of objects (ROBERTSON & BARON-COHEN,2017). The performance of motor skills depends on an intact sensory system, thus deficits in sensory processing may be associated with difficulties in interacting with other people and the environment, as well as in the performance of motor skills (LIU, 2013).

Children and adolescents with ASD may present impairments in generalized motor development, such as difficulties in fine/gross motor coordination and balance (CRAIG et al., 2018;OHARA, R et al., 2020BR Impaired motor skills are known to prevent children and

adolescents from participating in group physical activity programs (STINS, JF, & EMCK, C. 2018). Studies report that physical activity promotes greater independence, social interaction, communication and better motor skills (HUANG, J ET AL, 2020). In addition, an effective motor condition contributes to success in social participation in exercise programs (SOWA, M., & MEULENBROEK, R. 2012). Despite the reported benefits, children and adolescents with ASD have lower levels of physical activity when compared to their typically developing peers (MACDONALD ET AL., 2011BRSRINIVASAN ET AL., 2014BRSTANISH ET AL., 2017BRHILLIER ET AL., 2020BR Therefore, it is necessary to establish interventions that can increase physical activity levels in this population. One way to promote participation in recreational physical activities and sports is through interventions that can develop “physical literacy”.(CARLON et al., 2013)BR

“Physical literacy” describes the skills a person needs to engage in lifelong enjoyable physical activity, such as: 1) physical competence (e.g., gross motor ability); 2) psychological competence (eg motivation, engagement and self-regulation); 3) social competence (eg relationships and collaboration); and 4) cognitive competence (eg, content knowledge, knowledge and reasoning) (EDWARDS et al., 2017). The participation of children and adolescents in physical activities of recreation and sports requires skills in all these domains (EDWARDS et al., 2017; PATEL; SOARES; WELLS, 2017). The abilities of children and adolescents in all these domains of physical literacy encourage their participation in sports and recreational activities (EDWARDS et al., 2017; PATEL; SOARES; WELLS, 2017). In this context, sport-focused interventions that prepare the child or adolescent for the transition from the usual individual interventions to participation in community sports are necessary (CLUTTERBUCK; AULD; JOHNSTON, 2019a).

Recently, Clutterbuck, Auld & Johnston (2018) proposed a Sport Participation Model for children and adolescents with disabilities. The model includes 8 training sessions of group gross motor activities, lasting 1 hour, with activities focused on sport and carried out in the community, considering all domains of physical literacy 14 (figure 1). This model helps health professionals to identify different possibilities and opportunities that can increase participation in physical and sports activities (CLUTTERBUCK; AULD; JOHNSTON, 2018). Thus, the model has six distinct phases that represent the progression that children can achieve in terms of participation in physical activities throughout life. Interventions in the letter "P", are transitional interventions from individual health care to participation in conventional sports of

the “ORTS” phases. Designed to fill the “P” phase of Sports Stars, it is a modified sports intervention led by health professionals, which aims to prepare participants for the transition from usual care physical therapy to community sports participation (CLUTTERBUCK; AULD; JOHNSTON, 2018). The Sports Stars protocol includes training in culturally relevant sport-specific gross motor activities in a context designed to improve the confidence, motivation, and social skills needed for continued sport participation. The effects of Sports Stars were investigated in ambulatory Australian children with Cerebral Palsy (CP) and showed positive results in participation goals, gross motor activities and physical fitness components (CLUTTERBUCK; AULD; JOHNSTON, 2020a). In addition, in the perception of parents and therapists, Sports Stars improved the physical, social, psychological and cognitive components of children's physical literacy (CLUTTERBUCK; AULD; JOHNSTON, 2020b).

Currently, the effectiveness of Sports Stars Brasil is being investigated in Brazilian children and adolescents with CP (Sports Stars Brasil) by our research group (DE SOUSA JUNIOR, R. R et al., 2021). Despite the reported benefits of Sports Stars Australia, the effects of Sports Stars on the ASD population and its clinical application are still unknown. Thus, this project describes the investigation of the Sports Stars Brasil intervention in children and adolescents with ASD and aims to: 1) investigate the effectiveness of Sports Stars Brasil in comparison with the usual physiotherapeutic care; 2) explore the experiences of participants and their families who participated in the Sports Stars program and identify the results perceived by them regarding the domains of physical literacy;

Methods

This project will consist of three distinct phases:

1) Investigation of the effectiveness of Sports Stars Brasil in the population with ASD.
2) Evaluation of experiences of participants and their families.
3) Investigation of the measurement properties of the Ignite Challenge, Muscle Power, Gross Motor Development Test – second edition – TGMD-2, 10x5 Sprint test and Sprint instruments in children and adolescents with ASD.

Phase 1: Investigation of the effectiveness of Sports Stars Brasil in TEA

GOALS

Main goal

To evaluate the effectiveness of Sports Stars Brasil for children and adolescents with ASD, in terms of activity, participation and physical literacy, compared to the control group.

Specific objectives:

THEevaluate the benefits of*Sports Stars*Brazil in children and adolescents with ASDon activity outcomes (mobility and activity of daily living) and participation (frequency, involvement and responsibility).

investigate thebenefits of*Sports Stars*Brazil in children and adolescents with ASDon physical literacy outcomes (ie, physical, cognitive, psychological, and social domains).

Assumptions:

1. Children and adolescents who receive the Sports Stars Brasil program will demonstrate better levels of activity (mobility [eg, locomotion and manipulative skills] and activities of daily living); and participation (frequency, involvement [eg, sports and recreational activities], and responsibility) compared to those in the control group.

2. Children and adolescents who receive Sports Stars Brasil will demonstrate greater competences in physical literacy domains (ie, physical, cognitive, social and psychological domains) compared to the control group.

METHODS

design

Children will participate in a randomized controlled trial developed in accordance with the Items of the Standard Protocol for Randomized Interventional Trials (SPIRIT) (CHAN; TETZLAFF; ALTMAN, 2016) and will be registered on the Brazilian Registry platform of Clinical Trials (ReBEC). In addition, it will also be submitted to the Research Ethics Committee of the Federal University of Minas Gerais (UFMG). given difficulties in recruiting adolescents in this type of study, adolescents will participate in a feasibility study.

Randomized Controlled Trial

Approximately 38 children aged 6 years to 11 years, 11 months and 29 days will be recruited for convenience through disclosure on social networks through Instagram of the Sports Stars Brasil extension project. The sample size definition was based on the original Sports Stars results (CLUTTERBUCK; AULD; JOHNSTON, 2020a), in which an effect size of 1.05 was found on the activity and participation outcome (Modified Canadian Measure of Occupational Performance - COPM) in the post-treatment analysis between the Sports Stars group and the control group. A power of 80% and $\alpha = 95\%$ were considered, as well as a loss of 20% over time, using the GPower 3.1 software.

Viability study

For the study with adolescents, the sample size was calculated according to the equation shown below,which is based on criteria of unacceptable feasibility (red zone - 'STOP') versus acceptable feasibility (green zone - 'GO') [Lewis et al., 2021].

$$n = \left(\frac{Z_{1-\alpha}\sqrt{R_{UL}(1-R_{UL})}+Z_{1-\beta}\sqrt{G_{LL}(1-G_{LL})}}{(G_{LL}-R_{UL})}\right)^2 + \frac{1}{|G_{LL}-R_{UL}|}$$

Where: RUL= upper limit of the red zone; RUL= lower limit of the green zone; since they are insufficient for that. In that case, they should investigate uncertainties about Z1-α= probability of type I error; Z1-α= probability of type II error.

Therefore, determining that the adherence rate to the study is 65% (green zone), failure rate 35% (red zone), alpha of 5% and power of 80%, the sample for study feasibility would be 18 individuals in total .

eligibility criteria

Participants will be eligible for this study if they are between 6 and 18 years old at the start of the intervention, diagnosed with ASD, classified as mild to moderate on the Childhood Autism Rating Scale (CARS) (PEREIRA; RIESGO; WAGNER, 2008) and classified as by level I and II Autism Classification System of Functioning: Social Communication (ACSF): SC (Annex 2) (ELOI, et al., 2019). Participants will be excluded if they have cognitive, behavioral or clinical limitations (cardiorespiratory diseases) that prevent them from following the instructions and participating.Ther safely engage in physical activity in a group setting.

INTERVENTION PROCEDURES

Randomization and blinding

Children (n: 36) will be randomized into two groups: Sports Stars Brasil intervention and control group, which will receive usual therapy (physiotherapy and/or occupational therapy) or waiting list. Randomization will occur in blocks, with each randomization occurring when two subgroups of 4-5 participants in the child or adolescent age group are recruited. A random number generator will be used to create a random sequence of numbers. These numbers will be concealed in individually numbered opaque sealed envelopes. This sequence will be used to randomize children into the Sports Stars Brasil group or the control group. A new sequence will be used for each subgroup randomization until all 38 children are allocated or other participants

cannot be recruited. The same procedures will be performed with adolescents. Evaluators will be blinded to group allocation. Due to the intervention characteristics of this study, it is not possible toensure blinding ofinterventional individuals and therapists.

Sports Stars Brasil intervention group:This group intervention willthe participationfrom four to fiveindividualsin each group, which will be led by a physiotherapist, but will have the assistance of occupational therapy professionals andeducationphysicalTheBR The intervention will take place over eight weeks, with sessions once a week, lasting 1 hour. In each session they will be trained in this order: training of gross motor skills related to sports (running, jumping, ball activities). After that, participants in this group will be introduced to popular sports in Brazil: football, handball, basketball and athletics (Table 1). This intervention will be carried out in sports environments.

			**Skills**	**gross motor**		
	**Start**	**Heating**	**Locomotor**	**object control**	**Cooldown**	**cooling**
**Time**	10 min	5 min	15 min	15 min	10 min	5 min
**Activities**	Arrival of participants and family	active handling	Advanced skills (running, heels). Agility and coordination	catching and Playing kicking and receiving manipulation with balls	Experience in modified sports (football, handball, basketball and athletics)	Balance Stretching Decreased heart rate

Table 1. Characterization of Sports Stars sessions.

Since the ASD population may exhibit repetitive and stereotyped behaviors, irritability, aggression, hyperactivity, inattention and social impairment (DOYLE, CA & MCDOUGLE, CJ 2012), strategiesbased nthe Applied Behavior Analysis ABA will be used as facilitators to carry out the training of motor skills. Strategies based on ABA have shown benefits in mitigating these ASD behaviors and promoting a variety of social skills, communication and adaptive behaviors (CAMARGO; RISPOLI, 2013). To this end, children will be evaluated for their sensory profile, where possible sensory integration deficits will be identified, both hypo- and hyper-activity. Children and adolescents with ASD with a sensory hyporeactive profile may have a high neurological threshold of arousal and, therefore, need intense sensory inputs to be registered and provoke motivation for action. These individuals are usually passive, well behaved, silent, and may not be easily involved in activities. Intervention strategies, in cases of

hyperreactivity, consist of maintaining the appropriate level of alertness, so that the child does not adopt an attitude of flight, struggle or fear, in the face of stimuli that are beyond their capacity to tolerate (ANDRADE, MMAD 2020). Through the sensorial profile, the ABA strategies will be provided to the participants, through trained professionals.

Control Group - usual care or waiting list: Participants in this group will maintain their occupational therapy and/or conventional physical therapy intervention, or remain on the waiting list. It is believed that, in Brazil, children and adolescents with ASD receive, on average, one to two weekly individual therapy sessions in public or private clinics. All therapeutic activities undertaken by waiting group participants will be recorded in an activity diary.

PROCEDURES AND DATA COLLECTION

Characteristics of the participants

The age, sex and classification of the instruments of the Childhood Autism Rating Scale (CARS-BR) (Annex 1), Autism Classification System of Functioning: Social Communication (ACSF: SC) (Annex 2) and Abbreviated Sensory Profile 2 will be collected after signing of the terms of informed free consent (TCLE) (appendix A and B) signed by the parents or guardians and free informed consent (TALE) (appendix C and D) signed by the participating children or adolescents.

Childhood Autism Rating Scale(CARS-BR)

The CARS-BR scale is an instrument translated into Portuguese-Brazil, reliable and validated for the Brazilian population with ASD aged 3 to 17 years, with good internal consistency, discriminative validity, convergent validity and test-retest reliability of 0.90 (PEREIRA; RIESGO; WAGNER, 2008). CARS-BR is used in diagnoses and research related to ASD, in addition to helping to distinguish children and adolescents with other developmental delays. It is divided into 15 items, which assess behavior in 14 domains generally characteristic of ASD and a general severity diagnostic score domain, based on a four-point severity scale (absent disorder, mild to moderate or severe), the score ranges from 15 to 60, and the cutoff point for autism is 30 points (RAPIN; GOLDMAN, 2008; PEREIRA; RIESGO; WAGNER, 2008).

Autism Classification System of Functioning: Social Communication(ACSF: SC)

ACSF:SC is a classification system that aims to collect information, in a simplified and standardized, valid and reliable way, to characterize the communication skills of children with ASD in levels, for children aged 3 to 6 years (DI REZZE et al . 2016). The instrument was adapted for children with ASD and guaranteed equivalence between the original and translated versions, obtaining agreement rates above 90%. The classification is divided into 5 levels that distinguish the child's social communication skills according to social needs and goals (level V corresponds to when the child is reacting to other people's communication and the goal is known only by their parents or main caregivers; level IV is when the child is trying to initiate out of necessity and trying to respond to people he knows; level III the child tries to initiate and respond with social goals about his interests; level II the child communicates with other people, but they have difficulties if changes occur; level I the child sustains interaction and adapts to changes), the final classification is obtained based on information reported by parents and professionals familiar with the child's social communication skills, the higher the level, the better the performance of social communication and skills of communication (ELOI, et al., 2019). The instrument for the ASD population over 6 years of age is in the process of being translated by a researcher from this research group and will be used after this step is completed. but present difficulties if changes occur; level I the child sustains interaction and adapts to changes), the final classification is obtained based on information reported by parents and professionals familiar with the child's social communication skills, the higher the level, the better the performance of social communication and skills of communication (ELOI, et al., 2019). The instrument for the ASD population over 6 years of age is in the process of being translated by a researcher from this research group and will be used after this step is completed. but present difficulties if changes occur; level I the child sustains interaction and adapts to changes), the final classification is obtained based on information reported by parents and professionals familiar with the child's social communication skills, the higher the level, the better the performance of social communication and skills of communication (ELOI, et al., 2019). The instrument for the ASD population over 6 years of age is in the process of being translated by a researcher from this research group and will be used after this step is completed. better performance of social communication and communication skills (ELOI, et al., 2019). The instrument for the ASD population over 6 years of age is in the process of being translated by a researcher from this research group and will be used after this step is completed. better performance of social communication and communication skills (ELOI, et al., 2019). The instrument for the ASD population over 6 years of age is in the process of being translated by a researcher from this research group and will be used after this step is completed.

Sensory Profile 2 Abbreviated

The Abbreviated Sensory Profile 2 is a questionnaire designed to contribute to the assessment of the sensory performance of children and adolescents aged 3 to 14 years and 11 months, consisting of 34 items, which are answered by the caregiver about the frequency with which the behaviors occur in daily life. It is divided into a combination of scores from the sensory system (hearing, vision, touch, movement and attention), behavioral (behavioral, conduct, socio-emotional and attention) and sensory pattern (exploration, avoidance, sensitivity and observation) (DUNN, 2014) . The Abbreviated Sensory Profile 2 corresponds to the second edition of the Sensory Profile instrument (DUNN, 1999), it is composed of five questionnaires (Baby Sensory Profile 2, Young Child Sensory Profile 2, Child Sensory Profile 2, Abbreviated Sensory Profile 2, Sensory Profile 2 of School Monitoring), being selected, for this work, the sensory profile of the child (3 years to 14 years and 11 months) (DUNN, 2017). It is a judgment-based questionnaire and should be applied to those who have daily contact with the child/adolescent and are responsible for them. Each question describes children's responses to various sensory experiences. Responses should consider how often (always, often, occasionally, rarely, never) the behaviors occur, and scores are given from 1 to 5 (1 for "always" and 5 for "never"). The lower the score, the more evidence of sensory difficulties, that is, lower scores indicate greater severity of sensory problems, and the higher the score, the less evidence of sensory difficulties. being selected, for this work, the sensory profile of the child (3 years to 14 years and eleven months) (DUNN, 2017). It is a judgment-based questionnaire and should be applied to those who have daily contact with the child/adolescent and are responsible for them. Each question describes children's responses to various sensory experiences. Responses should consider how often (always, often, occasionally, rarely, never) the behaviors occur, and scores are given from 1 to 5 (1 for "always" and 5 for "never"). The lower the score, the more evidence of sensory difficulties, that is, lower scores indicate greater severity of sensory problems, and the higher the score, the less evidence of sensory difficulties. being selected, for this work, the sensory profile of the child (3 years to 14 years and eleven months) (DUNN, 2017). It is a judgment-based questionnaire and should be applied to those who have daily contact with the child/adolescent and are responsible for them. Each question describes children's responses to various sensory experiences. Responses should consider how often (always, often, occasionally, rarely, never) the behaviors occur, and scores are given from 1 to 5 (1 for "always" and 5 for "never"). The lower the score, the more evidence of sensory difficulties, that is, lower scores indicate greater severity of sensory problems, and the higher the score, the less evidence of

sensory difficulties. the sensory profile of the child (3 years to 14 years and eleven months) (DUNN, 2017). It is a judgment-based questionnaire and should be applied to those who have daily contact with the child/adolescent and are responsible for them. Each question describes children's responses to various sensory experiences. Responses should consider how often (always, often, occasionally, rarely, never) the behaviors occur, and scores are given from 1 to 5 (1 for "always" and 5 for "never"). The lower the score, the more evidence of sensory difficulties, that is, lower scores indicate greater severity of sensory problems, and the higher the score, the less evidence of sensory difficulties. the sensory profile of the child (3 years to 14 years and eleven months) (DUNN, 2017). It is a judgment-based questionnaire and should be applied to those who have daily contact with the child/adolescent and are responsible for them. Each question describes children's responses to various sensory experiences. Responses should consider how often (always, often, occasionally, rarely, never) the behaviors occur, and scores are given from 1 to 5 (1 for "always" and 5 for "never"). The lower the score, the more evidence of sensory difficulties, that is, lower scores indicate greater severity of sensory problems, and the higher the score, the less evidence of sensory difficulties. It is a judgment-based questionnaire and should be applied to those who have daily contact with the child/adolescent and are responsible for them. Each question describes children's responses to various sensory experiences. Responses should consider how often (always, often, occasionally, rarely, never) the behaviors occur, and scores are given from 1 to 5 (1 for "always" and 5 for "never"). The lower the score, the more evidence of sensory difficulties, that is, lower scores indicate greater severity of sensory problems, and the higher the score, the less evidence of sensory difficulties. It is a judgment-based questionnaire and should be applied to those who have daily contact with the child/adolescent and are responsible for them. Each question describes children's responses to various sensory experiences. Responses should consider how often (always, often, occasionally, rarely, never) the behaviors occur, and scores are given from 1 to 5 (1 for "always" and 5 for "never"). The lower the score, the more evidence of sensory difficulties, that is, lower scores indicate greater severity of sensory problems, and the higher the score, the less evidence of sensory difficulties. Responses should consider how often (always, often, occasionally, rarely, never) the behaviors occur, and scores are given from 1 to 5 (1 for "always" and 5 for "never"). The lower the score, the more evidence of sensory difficulties, that is, lower scores indicate greater severity of sensory problems, and the higher the score, the less evidence of sensory difficulties. Responses should consider how often (always, often, occasionally, rarely, never) the behaviors occur, and scores are given from 1 to 5 (1 for "always" and 5 for "never"). The lower the score, the more evidence of sensory

difficulties, that is, lower scores indicate greater severity of sensory problems, and the higher the score, the less evidence of sensory difficulties.

outcome measures

The outcome measures will take place at the beginning of the study (phase 1 - baseline), after 8 weeks of intervention (phase 2 - follow-up) and after 12 weeks of the beginning of the intervention (phase 3 - follow-up) by two evaluators blinded. Before the initial assessment, the evaluators will participate in training with the study team, to train the instruments that will be used in this study. The reliability of the evaluators was carried out by applying the tests to a minimum of 10 typical children and adolescents or those with ASD, as well as their parents or caregivers, when necessary.

Primary outcomes:

***Goal Entertainment Scaling*(GAS)**

The GAS is a functional scale to quantify the achievement or fulfillment of previously defined goals in an intervention program. (KIRESUK AND SHERMAN, 1968; TURNER – STOKES, 2003). According to manuals prepared by Turner - Stokes (2003), the procedure for applying the GAS consists of five steps: definition of therapeutic objectives; gradation of objectives regarding importance and difficulty; definition of the expected results with the intervention; classification of expected results (in five levels: -2, -1, 0, +1 and +2); calculation of the GAS score (by means of a formula developed by Kiresuk & Sherman (1968), with a description available in Turner – Stokes, 2003). In this scale, the child and/or his family rank the goals and priorities on a scale with scores from 0 to 3. Zero means no importance or difficulty and 3, maximum importance or difficulty. If the patient classifies any objective as 0, this objective must be renegotiated and replaced by another one of higher priority. For this study, three objectives will be identified. The first relates to the performance of an advanced, sports-specific gross motor activity (e.g., running, kicking, throwing, or jumping), the second refers to attendance at a physical recreation or sporting activity (e.g., watching a game football with friends twice a week) and the third refers to involvement during a physical recreation or sports activity (for example, being motivated to participate in a football game with friends). These goals will be selected and ranked by parents or guardians together with the participant and responsible therapist. If the patient classifies any objective as 0, this objective must be

renegotiated and replaced by another one of higher priority. For this study, three objectives will be identified. The first relates to the performance of an advanced, sports-specific gross motor activity (e.g., running, kicking, throwing, or jumping), the second refers to attendance at a physical recreation or sporting activity (e.g., watching a game football with friends twice a week) and the third refers to involvement during a physical recreation or sports activity (for example, being motivated to participate in a football game with friends). These goals will be selected and ranked by parents or guardians together with the participant and responsible therapist. If the patient classifies any objective as 0, this objective must be renegotiated and replaced by another one of higher priority. For this study, three objectives will be identified. The first relates to the performance of an advanced, sports-specific gross motor activity (e.g., running, kicking, throwing, or jumping), the second refers to attendance at a physical recreation or sporting activity (e.g., watching a game football with friends twice a week) and the third refers to involvement during a physical recreation or sports activity (for example, being motivated to participate in a football game with friends). These goals will be selected and ranked by parents or guardians together with the participant and responsible therapist. this objective should be renegotiated and replaced by another one of higher priority. For this study, three objectives will be identified. The first relates to the performance of an advanced, sports-specific gross motor activity (e.g., running, kicking, throwing, or jumping), the second refers to attendance at a physical recreation or sporting activity (e.g., watching a game football with friends twice a week) and the third refers to involvement during a physical recreation or sports activity (for example, being motivated to participate in a football game with friends). These goals will be selected and ranked by parents or guardians together with the participant and responsible therapist. this objective should be renegotiated and replaced by another one of higher priority. For this study, three objectives will be identified. The first relates to the performance of an advanced, sports-specific gross motor activity (e.g., running, kicking, throwing, or jumping), the second refers to attendance at a physical recreation or sporting activity (e.g., watching a game football with friends twice a week) and the third refers to involvement during a physical recreation or sports activity (for example, being motivated to participate in a football game with friends). These goals will be selected and ranked by parents or guardians together with the participant and responsible therapist. three objectives will be identified. The first relates to the performance of an advanced, sports-specific gross motor activity (e.g., running, kicking, throwing, or jumping), the second refers to attendance at a physical recreation or sporting activity (e.g., watching a game football with friends twice a week) and the third refers to involvement during a physical recreation or sports activity (for

example, being motivated to participate in a football game with friends). These goals will be selected and ranked by parents or guardians together with the participant and responsible therapist. three objectives will be identified. The first relates to the performance of an advanced, sports-specific gross motor activity (e.g., running, kicking, throwing, or jumping), the second refers to attendance at a physical recreation or sporting activity (e.g., watching a game football with friends twice a week) and the third refers to involvement during a physical recreation or sports activity (for example, being motivated to participate in a football game with friends). These goals will be selected and ranked by parents or guardians together with the participant and responsible therapist. the second refers to attending a physical recreation or sporting activity (for example, watching a soccer game with friends twice a week) and the third refers to being involved during a physical recreation or sporting activity (for example, being motivated when participating in a soccer game with friends). These goals will be selected and ranked by parents or guardians together with the participant and responsible therapist. the second refers to attending a physical recreation or sporting activity (for example, watching a soccer game with friends twice a week) and the third refers to being involved during a physical recreation or sporting activity (for example, being motivated when participating in a soccer game with friends). These goals will be selected and ranked by parents or guardians together with the participant and responsible therapist.

Participation and Environment Measure for children and young people (PEM-CY)

The PEM-CY (Annex 3) is an instrument that assesses the participation and environment of children and adolescents with disabilities, in their home, school and community environments (COSTER et al., 2011, 2012; GALVÃO et al., 2018) . It is one of the few assessments that combines a measure of participation and environment for children and young people. It is a tool that assesses the participation and environment of children and young people aged between 5 and 17 years, according to the perception of parents or caregivers. The measure identifies the involvement of children and young people in activities carried out at home, at school and in the community, as well as the characteristics of these environments that influence participation (BEDELL, GM et al). PEM-CYwas translated and validated for the Brazilian population in order to objectively measure the domain of participation in children (GALVÃO et al., 2018).This questionnaire comprises six different parts: frequency of participation, participation involvement, desire to change participation, support for the environment, support for the environment and environmental resources, and each subscale is scored at different intervals and converted into percentages (0% -100 %). A total score can be calculated by summing the

percentages of all dimensions. (COSTER et al., 2011, 2012; GALVÃO et al., 2018). It presents moderate to good internal consistency and test-retest reliability indices (COSTER et al., 2011) and was translated and culturally adapted for the Brazilian population by Galvão et al. (2018). The clinically significant difference for PEM-CY has yet to be determined,

Gross Motor Development Test-2 - TGMD-2

The TGMD-2 (Appendix 4) is a test for evaluating the gross motor development of boys and girls aged between three and 10 years. The test assesses 12 fundamental motor skills, of which six are locomotion skills (running, galloping, hopping, stepping, horizontally jumping and sideways running) and six are object control skills (batting, bouncing, catching, kicking, throwing over shoulder and roll a ball). For each skill, 3 to 5 specific motor criteria are observed. The TGMD-2 allows a separate evaluation of each subtest (locomotion and object control) and also in the object control subtest, a differentiation by gender. The total scores of each subtest are summed and represented as raw scores, which can be converted into motor quotients (ULRICH, 2000). The TGMD-2 was validated and reliable for Brazilian children in the study by Valentini et al., (2012) and has been used in children with ASD (KRUGER, SILVEIRA; MARQUES, 2019; MOHD NORDIN, A; ISMAIL, J & KAMAL NOR , N, 2021). The TGMD-2 has excellent reliability and validity rates (CAPIO; SIT; ABERNETHY, 2011a, 2011b). However, it does not show reliability values for the TEA population.

Ignite Challenge

The Ignite Challenge was created as an offshoot of the advanced 25-item Challenge test for independent outpatients with CP (ARBOR-NICITOPOULOS ET AL., 2018; 2021). The Ignite Challenge has two main components: 1) assessment of the quality and aspects of locomotor and object control skills and 2) it presents a dynamic assessment approach that may increase the child's desire to remain involved in the test, we believe that the test will have strong potential for children with ASD. A later adaptation of the Ignite Challenge refined its administration process for use with children with ASD. Ignite Challenge included a new introduction process that could include a social story, item 'picture cards' (ALLEN ET AL., 2017; LIU & BRESLIN, 2013) to accompany the evaluator's imitative demonstrations and allow the child to choose which item to do next (BRESLIN & RUDISILL, 2011), reducing the duration to two (instead of three) attempts for each item and greater emphasis on the use of cues direct and simplified verbal. The 'Ignite Challenge is a 13-item performance-based measure designed for use with children with autism spectrum disorder (ASD) who are 6 years and older, who are able to take

a 45- 60 minutes (including breaks) under the direction of the evaluator, without the guidance of parents or guardians. However, it is still not a test that has been validated for the Brazilian population. reducing the duration to two (instead of three) attempts for each item and greater emphasis on the use of direct and simplified verbal cues. The 'Ignite Challenge is a 13-item performance-based measure designed for use with children with autism spectrum disorder (ASD) who are 6 years and older, who are able to take a 45- 60 minutes (including breaks) under the direction of the evaluator, without the guidance of parents or guardians. However, it is still not a test that has been validated for the Brazilian population. reducing the duration to two (instead of three) attempts for each item and greater emphasis on the use of direct and simplified verbal cues. The 'Ignite Challenge is a 13-item performance-based measure designed for use with children with autism spectrum disorder (ASD) who are 6 years and older, who are able to take a 45- 60 minutes (including breaks) under the direction of the evaluator, without the guidance of parents or guardians. However, it is still not a test that has been validated for the Brazilian population. who are able to complete a 45-60 minute movement skills test (including intervals) under the evaluator's guidance, without parental or guardian guidance. However, it is still not a test that has been validated for the Brazilian population. who are able to complete a 45-60 minute movement skills test (including intervals) under the evaluator's guidance, without parental or guardian guidance. However, it is still not a test that has been validated for the Brazilian population.

Physical Literacy Profile Questionnaire(Annex 5)

The questionnaire measures the level of physical literacy of children, adolescents and young adults (6-21 years old). It was developed to identify goals and objectives for interventions related to sport and recreational activities. It consists of two parts and must be answered by caregivers, but children, adolescents and young adults can be present at the time of application and help clarify doubts. The questions are related to personal experience in sports activities (physical activities with competition and formal rules) and recreational activities (free physical activities aimed at health, well-being and fun, such as, for example, tag, dodgeball, hide and seek, among others). and performance (how the activity is performed) and satisfaction in the physical, social, psychological and cognitive domains. Each item rates performance on a 0-2 scale, and each item rates satisfaction with the child/adolescent/young adult's performance on a 1-10 point scale. The physical literacy questionnaire is being validated by this research group and may be excluded from this study if the psychometric properties do not show consistency for application.

***Pediatric Evaluation of Disability Inventory - computer adaptive test*(PEDI-CAT)**

THE PEDI-CAT was developed to measure performance in daily activities, mobility, cognitive-social and responsibility in children and adolescents up to 21 years of age (HALEY, SM et al 2011). Its application requires a computer with the instrument's software installed and can be self-administered (that is, completed by the child's parents), or with the presence of a trained professional to ensure the correct understanding of the information of each item (HALEY, SM et al 2011; HALEY, SM et al 2011). The PEDI-CAT includes domains thatalign with the International Classification of Functioning, Disability and Health for Children and Youth (ICF-CY) 'activity' domains (i.e. how the child performs activities in their normal daily environment) and 'participation' (is one's involvement in tasks of daily living). In the domains of daily activities, mobility and social cognitive, the four-point scores are based on different levels of difficulty. The responsibility domain rates items on a five-point scale, describing the division of responsibility between the caregiver and the child or adolescent in managing complex, multi-step life tasks. The overall score is transformed into a normative score (based on age) and a rolling score that will be used in the analyses. The PEDI-CAT+ASD includes additional instructions to help parents select an appropriate classification given the unique characteristics of children with autism, it includes new or revised items in the domains of daily activities, social/cognitive, and responsibility. The scale of items in the social/cognitive domain has been adjusted for the unique patterns of children and youth with autism, but scores are expressed on the same metric as the original PEDI-CAT. The other domains do not require any score adjustments for comparisons between versions. The PEDI-CAT was adapted and is reliable for use by children and young people with autism (KRAMER, JM et al 2012). It was translated and culturally adapted for the Brazilian population aged 0-21 years (MANCINI, MC et al. 2016). The PEDI-CAT was adapted and is reliable for use by children and young people with autism (KRAMER, JM et al 2012). It was translated and culturally adapted for the Brazilian population aged 0-21 years (MANCINI, MC et al. 2016). The PEDI-CAT was adapted and is reliable for use by children and young people with autism (KRAMER, JM et al 2012). It was translated and culturally adapted for the Brazilian population aged 0-21 years (MANCINI, MC et al. 2016).

The Muscle Power Sprint Test (MPST) is a simple field test to assess anaerobic performance in children and adolescents. The participant is asked to run as fast as possible for 15 m (marked by lines and cones), 6 times, with a 10-second interval between each sprint. It takes a few minutes to complete and only requires an open space, a timer and two cones. The MPST has

high interobserver and test-retest reliability (r = 0.97–0.99) for walking children with CP (VERSCHUREN O, TAKKEN T, KETELAAR M, GORTER JW, HOLDERS PJM, 2007). The properties in children and adolescents with ASD will be studied in this study.

The 10×5 Sprint Test (10×5ST) assesses agility and anaerobic capacity in ambulant children with CP. In this test, participants need to run 5 m separated by 2 cones, 10 times continuously, going around the cones that mark the end of the five meters (VERSCHUREN, TAKKEN, KETELAAR, GORTER, HELDERS, 2007). The 10 × 5 Meter Sprint Test has excellent interobserver (ICC > 0.97) and test-retest (r = 1) reliability, in addition to good reported construct validity. An increase in exercise time of 3.2 seconds is considered a real change (VERSCHUREN, TAKKEN, KETELAAR, GORTER, HELDERS, 2007). However, the psychometric properties have not yet been studied in the ASD population, so they will be investigated in this study.

For the participating adolescents and their guardians, in addition to the aforementioned instruments, information will be collected regarding the feasibility of the study: adherence to the intervention, satisfaction with the intervention, difficulty in understanding the offered intervention. These measures are collected in a semi-structured questionnaire (link:https://docs.google.com/forms/d/13ltHY0YXWBJQ5o8pey5K129P_qeTA4oRy_cXdG-QQJo/edit?usp=sharingBR

DATA ANALYSIS

Descriptive statistical analysis will be performed for participant characteristics (age, sex, CARS and ACSF classification: SC). Data will be analyzed for groups and separately for children (6-11 years, 11 months and 29 days years) and adolescents (12-18 years). Data normality will be investigated using the Shapiro-Wilk test Levene's test to verify the homogeneity of variance. Continuous data will be reported using mean and standard deviation (or median and interquartile if not normally distributed) and categorical data will be reported using frequency distributions. Mixed linear models will be used to evaluate the effects of Sports Stars Brasil compared to standard treatment on all outcomes. The t test (parametric variables) or Mann-Whitney test (nonparametric variables) will be performed for independent samples for comparison between groups, as well as the magnitude of the effect and statistical power. A significance level of 5% ($\alpha < 0.05$) will be adopted. To determine the reliability of the examiners, the reliability coefficients (intraclass correlation coefficient [ICC] or kappa [k]),

those with values greater than 0.70 were considered satisfactory. Statistical analyzes will be performed using the Statistical Package for the Social Sciences (SPSS) software.

RISKS AND BENEFITS

Scratchs:

1) Risk of tiredness or falls during sports activities, to minimize potential risks, there will always be maximum supervision by a trained professional next to the child or adolescent and in case of fatigue or tiredness, activities may be interrupted for a break.

2) Risk of embarrassment during the filming of the tests, to minimize the risk of embarrassment, the videos obtained during the filming will be kept in complete secrecy;

3) Risk of discomfort or embarrassment in answering the questionnaires. If this happens, any of the tests or questionnaires, as well as the program activities, may be interrupted at any time.

Benefits:

1) The information arising from the study may contribute to the elucidation of the possible benefits of a group physiotherapeutic treatment, centered on the performance of sports activities in children and adolescents with Autistic Spectrum Disorder (ASD);

2) The educational strategies that will be offered may favor the understanding of parents regarding the functional limitations of their children, optimizing health care and promoting functionality, social participation and physical literacy of children and adolescents with ASD, in different contexts .

TIMELINE

**Stage**	**Begin finish**
Writing of the project and approval of the departmental chamber	January 2022- February 2022
Submission to the ethics and research committee	March 2022-August 2022
Staff training and participant recruitment	August 2022- December 2022
Initial assessments and start of the Intervention (phase 1)	January 2023- March 2023
Reassessments (phase 1) and start of phase 2 and 3	March 2023- April 2023
Reassessments and follow-up (phase 1)	June 2023- July 2023
Data analysis	July 2023- November 2023
Scientific article writing	December 2023- April 2024

SAFETY MEASURES (COVID-19)

Due to the current period of the COVID-19 pandemic, we emphasize that the beginning of the project will only start, after prior authorization from the Graduate Program in Rehabilitation Sciences and Management of EEFFTO-UFMG, in line with the flexibility phases proposed by the University Federal of Minas Gerais. Possible changes to the schedule may occur and will be updated via Plataforma Brasil. All safety standards established by the competent bodies will be followed.

COLLECTION PLACE

The collections and interventions will be carried out in the open courts of the School of Physical Education, Physiotherapy and Occupational Therapy (EEFFTO) (Annex 6) and MultiLAB of the Federal University of Minas Gerais – UFMG, according to availability and scheduling.

BUDGET

Below are the main materials that will be used for this research and the estimated budget.

**Item**	**The amount**	**Material**	**Unitary value**	**Amount**
1	2 pct	A4 sheet	24.00	48.00
two	1000	impressions	0.50	500.00
3	4	basketballs	50.00	200.00
4	4	Soccer balls	40.00	160.00
5	4	futsal balls	45.00	180.00
6	4	baton relay	90.00	360.00
				**1,448.00**

Project costs such as sports and recreational materials will be borne by the researchers themselves.

Phase 2: Evaluating the experiences of Sports Stars intervention participants and their families

Study objectives

The objectives of this phase of the project are: (1) to explore the experiences of participants and their families who participated in the Sports Stars program and to identify

the results perceived by them regarding the domains of physical literacy, (2) to compare the perception of patients and parents/caregivers and (3) extract specific experiences about facilitators and barriers that helped and hindered participation in leisure activities for ASD children and adolescents, in order to inform practice and future research.

study design

Qualitative Study

Participants

Children and adolescents aged 6 to 18 years and their main caregiver, participants of the Sports Stars Brasil intervention (phase 1 intervention group) with ASD will be recruited for convenience.light to moderate inCARS and levelI and IIACSF: SCBR Participants who leave the Sports Stars program before its completion (8 meetings) will be excluded. Participants will be recruited until saturation which refers to the point during data collection at which links between qualitative data from focus groups have no more than 5% new categories of physical literacy and ICF compared to other groups focal.

Procedures and data collection:

After participating in the Sports Stars Brasil intervention, focus groups will be held to collect data with children, adolescents and one of the family members of each participant. For each focus group, data collection will be carried out only once and in person, with an expectation of duration for each interview of 1 hour, which can be extended if necessary. In order for homogeneity to occur in data collection, the responsible interviewers will undergo training.

Before each focus group, participants will be informed about the objectives of the study and if they agree to participate, they will sign the terms of consent and free and informed assent. The sample characterization data, considered important for the present study, which will be collected before the interviews: age, sex, classification and level of autism (in the case of children and adolescents), socioeconomic level and education. Data collection will take place at the School of Physical Education, Physiotherapy and Occupational Therapy at the Federal University of Minas Gerais. At the time of the interview, only the participants and interviewers will be present.

Focus groups:

Semi-structured questions will be asked with content related to physical literacy, in the context of the perception of performance in the domains of physical, social, psychological and cognitive competence and referring to barriers and facilitators in the participation of leisure activities within the ICF model that will be used as a reference for data analysis by linking qualitative data according to the rules proposed by Cieza. A et al. 2002. The focus groups will be carried out immediately after the intervention, the interview will be recorded and later transcribed.

After this process, the interviewee will have access to the transcript to confirm the data and information present before the content analysis by the researchers. In addition, during the interviews and conduction of the focus groups, if the evaluator deems it necessary, notes will be taken. With the consent of the participants, data analysis will be carried out.

Data analysis:

The testimonies of all participants will be transcribed verbatim. After this moment, the texts will be collected for qualitative analysis. The data will be analyzed through content analysis. Responses will be coded and categorized using NVivo software. The coding will be performed by two investigators to ensure agreement during coding, a third investigator will participate in this process in case of disagreement. Key citations around physical literacy themes will be identified and the codes will then be mapped to the category that most accurately corresponds to each code, using the criteria described by(EDWARDS et al., 2017).(table 1).

Regarding participation in leisure activities, barriers and facilitators, open questions will be used, adapted from (Longo, E, et al; 2020): “What leisure activities did you participate in in the last 2 months / 8 weeks, in addition to those you did at EEFFTO?”; “With whom and where did you do it? Did you enjoy doing the activities with them?"; "What other leisure activities would you like to do and didn't do?"; "What is an obstacle for you to do leisure activities? And what are the facilitators for you to do them? leisure activities?”; and “If it were possible, what would you change to improve your participation in leisure activities?” The moderator also made notes following each group.Quotations will be identified and coded within the five components of ICF functionality: (1) Body Functions;(2) Body Structures; (3) Activities and participation; (4) Environmental

factors; and (5) Personal factors, concepts that do not fall within the ICF components will be assigned the “not covered” category.

The components characterized in (Table 2) consist of chapters with ICF hierarchical categories as classification units. Where each ICF category is assigned an alphanumeric code and each classification is represented by a letter and followed by a second level specification this is a letter representing the classification component.

A preliminary coding summary will be shared with participants to assess the accuracy of the information collected.

**Table 1. Criteria for encoding Physical Literacy**	
1. **Physical**	Quotes related to physical ability, motor skills and fundamental movements.
2. **Social**	Quotations related to the child/adolescent's social relationships.
3. **psychological**	Quotes related to motivation, engagement, commitment and participation.
4. **Cognitive**	Quotes related to knowledge, reasoning, discernment, understanding of the child/adolescent

**Table 2. ICF coding criteria**	
1. **B**: Body Functions	Quotations referring to the physiological functions of body systems (including psychological functions)
2. **S:**Body Structures	Quotes referring to anatomical parts of the body such as organs, limbs and their components.
3. **D:**Activities and Participation	Quotes referring to the execution of a task or action by a child or adolescent with ASD and involvement in daily life situations.
4. **AND:**Environmental Factors	Citations referring to the physical, social and attitudinal environment in which a child or adolescent with ASD lives and leads their life.
5. **Facilitators and Barriers**	Quotes referring to facilitators and barriers that a child or adolescent with ASD may encounter in carrying out activities or problems that an individual may have when getting involved in life situations

Phase 3: measurement properties of the Ignite Challenge, 10x5 Sprint Test, Muscle Power Sprint Test andGross Motor Development Test – Second Edition – TGMD-2

Objective: To verify the reliability of the Ignite Challenge, 10x5 Sprint Test, Muscle Power Sprint Test and Gross Motor Development Test – second edition – TGMD-2

instruments, through the analysis of internal consistency and reproducibility (test-retest), with children and ASD teenagers.

study design

Cross-sectional observational study

Participants

Children and adolescents aged 6 to 18 years and their primary caregiver, participants of the Sports Stars Brasil intervention (phase 1 intervention group) with ASD classified by level of support will be recruited for convenience.levelI and IIthrough ACSF: SC.

sample calculation

The sample size will follow the recommendations of the Consensus-based Standards for the selection of health Measurement Instruments (COSMIN) (Terwee et al., 2012). According to Terwee et al. (2011) a sample of 50 individuals is suitable for analyzing the test-retest reliability and the measurement standard error.

Instruments

The Ignite Challenge is a 13-item ability-based measure that assesses the accuracy and speed of locomotor and object control skills for children with ASD from 6 years of age, classified in ACSF levels I and II. The Ignite Challenge was developed based on the 25-item Challenge test for children with CP (ARBOR-NICITOPOULOS ET AL., 2018; 2021). The Ignite Challenge uses “picture cards” for each test item to complement raters' demonstrations and improve understanding of each test item (ALLEN ET AL., 2017; LIU & BRESLIN, 2013) (BRESLIN & RUDISILL, 2011) . The Ignite Challenge demonstrated excellent inter-rater (ICC=0.91 (95%= 0.93, 0.99), intra-rater (ICC=0.96 (95% CI= 0.90, 0.98)) and rest test (ICC=0.91 (95% CI=0.84, 0,

The Muscle Power Sprint Test (MPST) is a simple field test to assess anaerobic performance in children and adolescents. The participant is asked to run as fast as possible for 15 m (marked by lines and cones), 6 times, with a 10-second interval between each sprint. It takes a few minutes to complete and only requires an open space, a timer and two cones. The MPST has high interobserver and test-retest reliability (r = 0.97–0.99) for walking children with CP (VERSCHUREN O, TAKKEN T, KETELAAR M, GORTER

JW, HOLDERS PJM, 2007). The properties in children and adolescents with ASD will be studied in this study.

The 10×5 Sprint Test (10×5ST) assesses agility and anaerobic capacity in ambulant children with CP. In this test, participants need to run 5 m separated by 2 cones, 10 times continuously, going around the cones that mark the end of the five meters (VERSCHUREN, TAKKEN, KETELAAR, GORTER, HELDERS, 2007). The 10 × 5 Meter Sprint Test has excellent interobserver (ICC > 0.97) and test-retest (r = 1) reliability, in addition to good reported construct validity. An increase in exercise time of 3.2 seconds is considered a real change (VERSCHUREN, TAKKEN, KETELAAR, GORTER, HELDERS, 2007). However, the psychometric properties have not yet been studied in the ASD population, so they will be investigated in this study.

The TGMD-2 (Appendix 4) is a test for evaluating the gross motor development of boys and girls aged between three and 10 years. The test assesses 12 fundamental motor skills, of which six are locomotion skills (running, galloping, hopping, stepping, horizontally jumping and sideways running) and six are object control skills (batting, bouncing, catching, kicking, throwing over shoulder and roll a ball). For each skill, 3 to 5 specific motor criteria are observed. The TGMD-2 allows a separate evaluation of each subtest (locomotion and object control) and also in the object control subtest, a differentiation by gender. The total scores of each subtest are summed and represented as raw scores, which can be converted into motor quotients (ULRICH, 2000). The TGMD-2 was validated and reliable for Brazilian children in the study by Valentini et al., (2012) and has been used in children with ASD (KRUGER, SILVEIRA; MARQUES, 2019; MOHD NORDIN; ISMAIL & KAMAL NOR, 2021). The TGMD-2 has excellent reliability and validity rates (CAPIO; SIT; ABERNETHY, 2011a, 2011b). However, it does not show reliability values for the TEA population.

Procedures

reliability

The test-retest reliability of the instruments will be performed at two different times, within an interval of seven to 10 days, by the same examiner who will assess the 50 children and adolescents. These assessments will be filmed with a video camera and scored later by the examiner. A second examiner will assist and score each participant's

first assessment for inter-rater reliability analysis. Both examiners are certified to administer the*Ignite Challenge*BR For the application of other instrumentsno certification is required, just prior training.

Responsiveness and Rates of Change

After the first test-retest reliability assessment, participants who have undergone intervention (weekly physical therapy treatment, individual or group, for 30 to 45 minutes) lasting three months, the instruments will be administered again and filmed on a video camera. An evaluator will watch the videos of the first evaluation of the test-retest reliability analysis and the reevaluation and score these evaluations, he will be blinded to the date. The anchor-based approach will be used for analysis of the minimal clinically important change index (MMCI). In this approach, the index is calculated based on an external criterion (Copay et al., 2007). When carrying out this approach, it must be ensured that the anchor used is capable of identifying the participants who achieved a clinically important change (Engel et al., 2018). A three-point Global Scoring Scale (worsened, unchanged, improved) will be used as an external criterion (Guyatt et al., 2002). After two months of treatment, participants' parents/guardians will be asked how they rate changes in their child's gross motor function. Participants classified as “improved” in the parents' perception will be considered as presenting a clinically important change in the instruments.

Statistical analysis

The Intraclass Correlation Coefficient (ICC) type will be used to assess the reliability of the total score for each of the instruments. Values below 0.74 represent moderate to poor reliability, between 0.75 and 0.89 indicate good reliability and values above 0.90, excellent (Portney & Walkins, 2009). The Weighted Kappa index (k) with the method of incremental weights will be used to assess the reliability of each test item (Portney & Walkins, 2009). Values below 0.20 represent poor reliability, values between 0.20 and 0.40 suggest fair reliability, between 0.41 and 0.60 moderate, values between 0.61 and 0.80 substantial and above 0.80 almost perfect (Landis & Koch et al., 2012). Confidence intervals at 95% (CI 95%) accompanied the indices. Furthermore, combined measurement standard error (SEM) and coefficient of variation (CV) will be used as

indices of variation. CV values will be considered adequate when below 10% (Bruton et al., 2000). In order to complement the results of the reliability indices, the independent t-test will be performed in the inter-examiner reliability analysis and the paired t-test, in the test-retest reliability. In addition, the BlandAltman analysis with its limits of agreement at 95% will be used to graphically illustrate the variation of instrument scores for each participant in relation to the mean of the two occasions (test-retest) or of the two raters (inter-rater). (Bruton et al., 2000). The instrument's responsiveness will be analyzed by the paired t-test. Measures effect size (d) of magnitude between 0.20 and 0. 50 revealed a small effect, between 0.50 and 0.80 a moderate effect and above 0.80 a large effect (COHEN, 1988). The MMD will be estimated with two confidence intervals: 90% (MMD90) for comparison with the value reported in the original version of the instrument, and 95% (MMD95) to aid in the interpretation of change values (Terwee et al., 2007), using the formulas below: $MMD90 = 1.65 \times \sqrt{2} \times EPM$ and $MMD95 = 1.96 \times \sqrt{2} \times EPM$ Where EPM = measurement standard error.

Prior to calculating the MMCI, the Spearman Rank Correlation Coefficient (rs) will estimate the correlation between changes in instrument scores after two months of treatment and parents/guardians' perception of change on the Global Score Scale; where r greater than or equal to 0.30 indicate that the anchor is considered adequate to estimate the MMCI (Revicki et al., 2008). Then, the MMCI will be estimated using the moving average method (Engel et al., 2018), where the index is considered the average test score value, among participants classified as those who improved according to the Global Score Scale . All analyzes were conducted using the Statistical Package for the Social Sciences (SPSS), version 19.0.

REFERENCES

American Psychiatric Association. Diagnostic and Statistical Manual of Mental Disorders: DSM-5. 5. ed. Porto Alegre: Artmed; 2014. 848 p.

ANDRADE, MMA Analysis of the influence of the Ayres® sensory integration approach on the school participation of students with autism spectrum disorder. 2020.

BARROS, SSH Pattern of practice of physical activities of children in preschool age. [Dissertation]. 2005. Graduate in Physical Education at the Federal University of Santa Catarina.

BEATON, DE et al. Guidelines for the process of cross-cultural adaptation of self-report measures. Spine, v. 25, no. 24, pg. 3186-3191, 2000.

BEDELL, GM et al. Parent perspectives to inform development of measures of children's participation and environment. Archives of Physical Medicine and Rehabilitation, v. 92, no. 5, p. 765-773, 2011.

BURDETTE, HL et al. parental report O fbillboard playtime as a measure of physical activity in preschool children. Archives of Pediatrics & Adolescent Medicine, 2004;158(4):353-357.

CAMARGO, SPH; RISPOLI, M. Applied behavior analysis as an intervention for autism: definition, characteristics and philosophical assumptions. Special Education Magazine, v. 26, no. 47, p. 639-650, Sep./Dec, 2013.

CAMARGO, SPH; RISPOLI, M. Applied behavior analysis as an intervention for autism: definition, characteristics and philosophical assumptions. Special Education Magazine, v. 26, no. 47, p. 639-650, Sept./Dec., 2013 apud SKINNER, BF Science and human behavior. New York: Free Press, 1953.

CAPIO, CM; SIT, CHP; ABERNETHY, B. Fundamental movement skills testing in children with cerebral palsy. Disability and Rehabilitation, v. 33, no. 25–26, p. 2519–2528, 2011b.

CARLON, SL et al. Differences in usual physical activity levels of young people with cerebral palsy and their typically developing peers: A systematic review. Disability and Rehabilitation, v. 35, no. 8, p. 647–655, 2013.

CHAN, A.; TETZLAFF, JM; ALTMAN, DG SPIRIT 2013 Statement : Defining Standard Protocol Items for Clinical Trials. Ann Intern Med, v. 158, no. 3, p. 200–207, 2016.

CIEZA, A. et al. Linking health-status measurements to the international classification of functioning, disability and health. Journal of rehabilitation medicine, v. 34, no. 5, p. 205-210, 2002.

CLUTTERBUCK, G.; AULD, M.; JOHNSTON, L. Active exercise interventions improve gross motor function of ambulant/semi-ambulant children with cerebral palsy: a systematic review. Disability and Rehabilitation, v. 41, no. 10, p. 1131–1151, 2019a.

CLUTTERBUCK, GL; AULD, ML; JOHNSTON, LM SPORTS STARS study protocol: A randomized, controlled trial of the effectiveness of a physiotherapist-led modified sport intervention for ambulant school-aged children with cerebral palsy. BMC Pediatrics, v. 18, no. 1, p. 1–10, 2018.

CLUTTERBUCK, GL; AULD, ML; JOHNSTON, LM Performance of school-aged children with cerebral palsy at GMFCS levels I and II on high-level, sports-focussed gross motor assessments. Disability and Rehabilitation, v. 0, no. 0, p. 1–9, 2019b.

CLUTTERBUCK, Georgina L.; AULD, Megan L.; JOHNSTON, Leanne M. SPORTS STARS: a practitioner-led, peer-group sports intervention for ambulant children with cerebral palsy. Activity and participation outcomes of a randomized controlled trial. Disability and Rehabilitation, p. 1-9, 2020a.

CLUTTERBUCK, Georgina L.; AULD, Megan L.; JOHNSTON, Leanne M. SPORTS STARS: a practitioner-led, peer-group sports intervention for ambulant, school-aged children with cerebral palsy. Parent and physiotherapist perspectives. Disability and Rehabilitation, p. 1-10, 2020b.

COLOMBO-DOUGOVITO, AM; BLOCK, ME; ZHANG, X.; STREHLI, I. A multiple-method review of accommodations to gross motor assessments commonly used with children and adolescents on the autism spectrum. Autism, 24, no. 3, p. 693-706, 2020.

COSTER, W. et al. Psychometric evaluation of the Participation and Environment Measure for Children and Youth. Developmental Medicine and Child Neurology, v. 53, no. 11, p. 1030–1037, 2011.

COSTER, W. et al. Development of the participation and environment measure for children and youth: Conceptual basis. Disability and Rehabilitation, v. 34, no. 3, p. 238–246, 2012.

CRAIG, F. et al. Motor Competency and Social Communication Skills in Preschool Children with Autism Spectrum Disorder. Autism Research, v. 11, p. 893–902, 2018.

DI REZZE, Briano et al. Developing a classification system of social communication functioning of preschool children with autism spectrum disorder. Developmental Medicine & Child Neurology, v. 58, no. 9, p. 942-948, 2016.

DOYLE, CA & MCDOUGLE, CJ Pharmacologic treatments for the behavioral symptoms associated with autism spectrum disorders across the lifespan. Dialogues in clinical neuroscience, v. 14, no. 3, p. 263, 2012.

DUNN, W. Caregiver Questionnaire–Sensory Profile. United States of America, 1999.

DUNN, W. Sensory profile 2: user's manual. San Antonio: NCS Pearson, 2014.

EDWARDS, LC et al. Definitions, Foundations and Associations of Physical Literacy: A Systematic Review. Sports Medicine, v. 47, no. 1, p. 113–126, 2017.

ELOI, DS et al. Cross-cultural adaptation of the Autism Classification System of Functioning: Social Communication (ACSF: SC) instrument for use in Brazil. Cad. Bras. To have. Occup., São Carlos, vol. 27, no. 2, p. 293-301, 2019.

GALVAO, yes. RVP et al. Participation and Environment Measure - Children and Youth (PEM-CY). Journal of Occupational Therapy of the University of São Paulo, v. 29, no. 3, p. 237–245, 2018.

HALEY, SM et al. PEDI-CAT Version 1.4. 0: development, standardization and administration manual. Boston: Trustees of Boston University, 2011.

HALEY, SM et al. Accuracy and precision of the Pediatric Evaluation of Disability Inventory computerBRadaptive tests (PEDIBRCAT). Developmental Medicine & Child Neurology, v. 53, no. 12, p. 1100-1106, 2011.

HILLIER, A; BUCKINGHAM, A; SCHENA, D. Physical activity among adults with autism: participation, attitudes, and barriers. Perceptual and Motor Skills, v. 127, no. 5, p. 874-890, 2020.

HOWELLS, K et al. Efficacy of group-based organized physical activity participation for social outcomes in children with autism spectrum disorder: a systematic review and meta-analysis. Journal of autism and developmental disorders, v. 49, no. 8, p. 3290-3308, 2019.

HUANG, J et al. "Meta-Analysis on Intervention Effects of Physical Activities on Children and Adolescents with Autism." International journal of environmental research and public health vol. 17.6 1950. 17 Mar. 2020

KHOURY, LP et al. Behavioral management of children with Autism Spectrum Disorders in condition of school inclusion: Guidance guide for teachers [electronic book]. São Paulo: Editora MEMNON, 2014.

KRAMER, JM et al. "A new approach to the measurement of adaptive behavior: development of the PEDI-CAT for children and youth with autism spectrum disorders." Physical & occupational therapy in pediatrics vol. 32.1 (2012).

KRIEGER, B et al. Cross-cultural adaptation of the Participation and Environment Measure for Children and Youth (PEM-CY) into German: a qualitative study in three countries. BMC pediatrics, v. 20, no. 1, p. 1-15, 2020.

KRUGER, GR; SILVEIRA, JR; MARQUES, AC Motor skills of children with autism spectrum disorder. Brazilian Journal of Kinanthropometry & Human Performance, 21, 2019.LAW, M. et al. Canadian Occupational Performance Measure (COPM). Organization and Translation Lívia de Castro Magalhães, Lílian Vieira Magalhães, Ana Amélia Cardoso. Belo Horizonte: Editora UFMG, 2009.

Lewis M, Bromley K, Sutton CJ, McCray G, Myers HL, Lancaster GA. Determining sample size for progression criteria for pragmatic pilot RCTs: the hypothesis test strikes back!. Pilot Feasibility Stud. 2021;7(1):40. Published 2021 Feb 3. doi:10.1186/s40814-021-00770-x.

LI, YONGBRJIANG et al. Global prevalence of obesity, overweight and underweight in children, adolescents and adults with autism spectrum disorder, attentionBRdeficit hyperactivity disorder: A systematic review and metaBRanalysis. Obesity Reviews, v. 21, no. 12, p. e13123, 2020.

LIU, T. Sensory Processing and Motor Skill Performance in Elementary School Children with Autism Spectrum Disorder. Perceptual & Motor Skills: Physical Development & Measurement, v. 116, no. 1, p. 197-209, 2013.

MAENNER, MJ et al. Prevalence of Autism Spectrum Disorder Among Children Aged 8 Years — Autism and Developmental Disabilities Monitoring Network, 11 Sites, United States, 2016. Morbidity and Mortality Weekly Report, v. 69, no. 4, March, 2020.

MANCINI, MC et al. New version of the Pediatric Evaluation of Disability Inventory (PEDI-CAT): translation, cultural adaptation to Brazil and analyzes of psychometric properties. Brazilian journal of physical therapy, v. 20, no. 6, p. 561-570, 2016.

MASSION, J. Sport et autism. Science & Sports, vol. 21, p. 243-248, 2006.

MACDONALD, M; ESPOSITO, P; ULRICH, D. The physical activity patterns of children with autism. BMC research notes, v. 4, no. 1, p. 1-5, 2011.

MATTOS, JC; D'ANTINO, MEF; CYSNEIROS, RM Translation into Brazilian Portuguese and cultural adaptation of the Sensory Profile. Psychology: theory and practice, vol. 17, no. 3, p. 104-120, 2015.

MOHD NORDIN, A; ISMAIL, J & KAMAL NOR, N. Motor development in children with autism spectrum disorder. Frontiers in pediatrics, p. 889, 2021.

OBRUSNIKOVA, I.; CAVALIER, AR Perceived Barriers and Facilitators of Participation in After-School Physical Activity by Children with Autism Spectrum Disorders. Journal of Developmental Physical Disabilities, vol. 23, p.195–211, 2011.

OHARA, Reiko et al. Association between social skills and motor skills in individuals with autism spectrum disorder: a systematic review. European Journal of Investigation in Health, Psychology and Education, v. 10, no. 1, p. 276-296, 2020.

PAN, CY; FREY, GC Identifying Physical Activity Determinants in Youth with Autistic Spectrum Disorders. Journal of Physical Activity and Health, v. 2, p. 412-422, 2005.

PATEL, DR; SOARES, N.; WELLS, K. Neurodevelopmental readiness of children for participation in sports. Translational Pediatrics, v. 6, no. 3, p. 167–173, 2017.

PEREIRA, A.; RIESGO, RS; WAGNER, MB Childhood autism: translation and validation of the Childhood Autism Rating Scale for use in Brazil. Journal of Pediatrics, v. 84, no. 6, p. 487-494, 2008.

Portney, L., & Watkins, M. (2000). Power and sample size. Foundations of ClinicalResearch. New Jersey: Prentice Hall Health, 705-30.

PORTNEY, Leslie Gross et al. Foundations of clinical research: applications to practice. Upper Saddle River, NJ: Pearson/Prentice Hall, 2009.

RAPIN, I.; GOLDMAN, S. The Brazilian CARS: a standardized screening tool for autism. Journal of Pediatrics, v. 84, no. 6, 2008.

ROBERTSON, CE; BARON-COHEN, S. Sensory perception in autism. Nature Reviews Neuroscience, v. 18, no. 11, p. 671-684, 2017.

ROSENBAUM, P.; GORTER, JW The “F-words” in childhood disability: I swear this is how we should think. Child: Care, Health and Development, v. 38, no. 4, p. 457–463, 2012.

SIMPSON, K et al. Investigating the participation of children on the autism spectrum across home, school, and community: A longitudinal study. Child: care, health and development, v. 45, no. 5, p. 681-687, 2019.

BRAZILIAN SOCIETY OF PEDIATRICS. Guidance Manual Scientific Department of Developmental and Behavioral Pediatrics Autism Spectrum Disorder, n. 5, Apr, 2019.

SOWA, M; MEULENBROEK, R. Effects of physical exercise on autism spectrum disorders: a meta-analysis. Research in autism spectrum disorders, v. 6, no. 1, p. 46-57, 2012.

SRINIVASAN, S M.; PESCATELLO, L S.; BHAT, A N. Current perspectives on physical activity and exercise recommendations for children and adolescents with autism spectrum disorders. physical therapy, v. 94, no. 6, p. 875-889, 2014.

STANISH, HI et al. Physical activity levels, frequency, and type among adolescents with and without autism spectrum disorder. Journal of autism and developmental disorders, v. 47, no. 3, p. 785-794, 2017.

STINS, J F.; EMCK, C. Balance performance in autism: A brief overview. Frontiers in psychology, v. 9, p. 901, 2018.

TERWEE C, BOT S, BOER M, WINDT D, KNOL D, DEKKER J, BOUTER L, VET H. Quality Criteria were Proposed for Measurement Properties of Health Status Questionnaires. Journal of Clinical Epidemiology. 2007; 60: 34-42.

TERWEE, CB et al. Rating the methodological quality in systematic reviews of studies on measurement properties: a scoring system for the COSMIN checklist. Quality of life research, v. 21, no. 4, p. 651-657, 2012.

TERWEE CB, MOKKINK LB, KNOL DL, OSTELO RWJG, BOUTER LM, DE VET HCW Rating the methodological quality in systematic reviews of studies on measurement properties: a scoring system for the COSMIN checklist. Quality of Life Research 2011, July 6

ULRICH, DA Test of Gross Motor Development, 2nd ed. n. June, 2000.

VARNI, JW; BURWINKLE, TM; SEID, M. The PedsQL TM 4.0 as a school population health measure: Feasibility, reliability, and validity. Quality of Life Research, v. 15, no. 2, p. 203–215, 2006.

VERSCHUREN, O. et al. Reliability for Running Tests for Measuring Agility and Anaerobic Muscle Power in Children and Adolescents with Cerebal Palsy. [sd].

VERSCHUREN, O. et al. Validity of the muscle power sprint test in ambulatory youth with cerebral palsy. Pediatric Physical Therapy, v. 25, no. 1, p. 25–28, 2013.7

VERSCHUREN, Olaf et al. Exercise and physical activity recommendations for people with cerebral palsy. Developmental Medicine & Child Neurology, v. 58, no. 8, p. 798-808, 2016.

WILLIAMS, K et al. Functioning, participation, and quality of life in children with intellectual disability: an observational study. Developmental Medicine & Child Neurology, v. 63, no. 1, p. 89-96, 2021.

World Health Organization, The ICD-10 Classification of Mental and Behavioral Disorders: Clinical Descriptions and Diagnostic Guidelines. Geneva, Switzerland World Health Organization 1992;

ANNEX 1 - CARS - CHILDHOOD AUTISM RATING SCALE

CARS-Childhood Autism Rating Scale PORTUGUESE VERSION

I. PERSONAL RELATIONS

**1**	No evidence of difficulty or abnormality in personal relationships: The behavior of the child is age-appropriate. Some shyness, nervousness or annoyance may be observed when the child is told what to do, but not to an unusual degree.
1.5	
**two**	Mildly Abnormal Relationships: The child may avoid looking the adult in the eye, avoid the adult or overreact if interaction is forced, be overly shy, not respond to the adult as expected, or cling to the parent a little more than most of the children of same age
2.5	
**3**	Moderately abnormal intercourse: Sometimes the child is indifferent (appears to ignore the adult). At other times, persistent and vigorous attempts are necessary to get the child's attention. Child-initiated contact is minimal.
3.5	
**4**	Severely Abnormal Relationships: Child is consistently unresponsive or unaware of what the adult is doing. She almost never responds or initiates contact with the adult. Only the most persistent attempt to attract attention has any effect.
	**Comments:**

	II. IMITATION
**1**	Appropriate imitation: The child can imitate sounds, words and movements, which are appropriate for his or her skill level.
1.5	
**two**	Slightly abnormal imitation: Most of the time, the child imitates simple behaviors such as clapping or isolated verbal sounds; occasionally imitates only after stimulation or delay.
2.5	
**3**	Moderately Abnormal Imitation: The child imitates only part of the time and requires a great deal of adult persistence or help; often imitates only after a while (with delay).
3.5	
**4**	Severely Abnormal Imitation: Does the child rarely or never imitate sounds, words, or movements even with stimulation and assistance.
	**Comments:**

III. EMOTIONAL RESPONSE

**1**	Emotional response appropriate to situation and age: Child demonstrates appropriate type and degree of of emotional response, indicated by a change in facial expression, posture, and demeanor.
1.5	
**two**	Mildly Abnormal Emotional Response: The child occasionally has an inappropriate type or degree of emotional response. Sometimes your reactions are not related to objects or to events around you.
2.5	
**3**	Moderately Abnormal Emotional Response: The child demonstrates clear signs of inappropriate emotional response (type or degree). Reactions may be quite inhibited or excessive and unrelated to the situation; may grimace, laugh, or become rigid even when not emotion-producing objects or events are present.
3.5	
**4**	Severely abnormal emotional response: Responses are rarely appropriate to the situation. Once a child reaches a certain mood, it is very difficult to change it. On the other hand, the child may show different emotions when nothing has changed.
	**Comments:**

IV. BODY USE

**1**	Age-appropriate body use: The child moves with the same ease, agility and coordination of a normal child of the same age.
1.5	
**two**	Slightly abnormal body use: Some peculiarities may be present, such as clumsiness, repetitive movements, poor coordination, or the rare presence of unusual movements
2.5	
**3**	Moderately Abnormal Body Use: Behaviors that are clearly odd or unusual for a child this age may include awkward finger movements, peculiar finger or body posture, staring, body pinching, self-harm, rocking, spin or walk on tiptoe.
3.5	
**4**	Severely Abnormal Body Use: Heavy or frequent movements of the type listed above are signs of severely abnormal body use. These behaviors may persist despite attempts to discourage children from doing them or to involve the child in other activities. activities.
	**Comments:**

V. USE OF OBJECTS

**1**	Appropriate Use of and Interest in Toys and Other Objects: Does the child show an interest normal for toys and other objects appropriate to his or her skill level and uses them appropriately.
1.5	
**two**	Mildly inappropriate use of and interest in toys and other objects: The child may show an unusual interest in a toy or play with it inappropriately, in a childish way (example: hitting or sucking on the toy)
2.5	
**3**	Moderately inappropriate use of and interest in toys and other objects: The child may show little interest in toys or other objects, or may be concerned about using them in strange ways. She can concentrate on some insignificant part of the

	toy, becoming fascinated with the light reflecting off it, repetitively moving something part of the object or exclusively play with it.
3.5	
**4**	Severely inappropriate use of and interest in toys and other objects: The child may engage in the same behaviors as above, but more frequently and intensity. It is difficult to distract the child when he is engaged in these inappropriate activities.
	**Comments:**

	SAW. RESPONSE TO CHANGES
**1**	Age-appropriate responses to change: Although the child may notice or comment on changes in routine, he is able to accept these changes without undue distress.
1.5	
**two**	Mildly abnormal age-appropriate responses to change: When an adult tries to change tasks, the child can continue in the same activity or use the same materials.
2.5	
**3**	Moderately abnormal age-appropriate responses to change: The child actively resists changes in routine, tries to continue his old activity, and is difficult to distract. she can becoming unhappy and angry when an established routine is changed.
3.5	
**4**	Severely abnormal age-appropriate responses to change: Child demonstrates serious to the changes. If a change is forced, she may become extremely angry or unwilling to help and respond with tantrums.
	**Comments:**

	VII. VISUAL RESPONSE
**1**	Adequate visual response: The child's visual behavior is normal and appropriate for his or her age. Vision is used in conjunction with other senses as a way to explore a new object.
1.5	
**two**	Slightly abnormal visual response: The child occasionally needs to be reminded to look at objects. The child may be more interested in looking at mirrors or lights than peers, may occasionally stare into space, or may avoid looking at people in the eyes.
2.5	
**3**	Moderately Abnormal Visual Response: Child must be reminded frequently to look at what he/she is doing, may stare into space, avoid looking at people in eyes, looking at objects from an unusual angle, or holding objects too close to the eyes.
3.5	
**4**	Severely abnormal visual response: The child constantly avoids looking at people or certain objects and may demonstrate extreme forms of other visual peculiarities described above.
	**Comments:**

	VIII. AUDITORY RESPONSE
**1**	Age-appropriate auditory responses: The child's auditory behavior is normal and age-appropriate. Hearing is used along with other senses.
1.5	
**two**	Mildly abnormal auditory responses: There may be no response or a slightly exaggerated response to certain sounds. Responses to sounds can be delayed and sounds can need repetition to hold the child's attention. The child can be distracted by external sounds.
2.5	
**3**	Moderately abnormal auditory responses: At The child's responses to the sounds vary. Often ignores the sound the first few times it is made. You can startle or cover up ears when listening to some everyday sounds.
3.5	
**4**	Severely abnormal auditory responses: Child overreacts and/or dismisses sounds to an extremely significant degree, regardless of the type of sound.
	**Comments:**

	IX. RESPONSE AND USE OF TASTE, SMELL AND TOUCH
**1**	Normal use and response of taste, smell, and touch: The child explores new objects in an age-appropriate way, usually by feeling or looking. Taste or smell can be used when appropriate. When reacting to small everyday pains, the child expresses discomfort but do not overreact.
1.5	
**two**	Slightly abnormal use and response of taste, smell, and touch: Child may persist in putting objects in mouth; can smell or taste/taste inedible objects. May ignore or slightly overreact to minor pain, to which a normal child would express just discomfort.
2.5	
**3**	Moderately abnormal use and response of taste, smell, and touch: The child may be moderately preoccupied with touching, smelling, or tasting objects or people. The child may react too much or too little.
3.5	
**4**	Severely abnormal use and response of taste, smell, and touch: The child is preoccupied with smelling, tasting, and feeling objects, more for sensation than for normal exploration or use of objects. The child may completely ignore the pain or react very strongly to discomfort. light.
	**Comments:**

	X. FEAR OR NERVOUSNESS
**1**	Normal Fear or Nervousness: The child's behavior is appropriate to both the situation and the age
1.5	
**two**	Mildly abnormal fear or nervousness: The child occasionally shows too much or too little fear or nervousness compared to the reactions of a normal child. age and similar situation.
2.5	

3 Moderately abnormal fear or nervousness: Does the child show significantly more or significantly less fear than would be typical for a younger or older child in a situation similar.

3.5

4 Severely abnormal fear or nervousness: Fears persist even after repeated experiences with harmless events or objects. It is extremely difficult to calm or comfort the child. The child may, on the other hand, fail to show adequate regard for risks that other children of the same age avoid.

Comments:

XI. VERBAL COMMUNICATION

1 Normal verbal communication, appropriate for age and situation.

1.5

two Slightly abnormal verbal communication: Speech demonstrates global delay. most of the speech has meaning; however, some echolalia or pronominal inversion may occur. Some peculiar words or jargon may be used occasionally.

2.5

3 Moderately abnormal verbal communication: Speech may be absent. When present, verbal communication may be a mixture of some meaningful speech and some peculiar language, such as jargon, echolalia, or pronominal inversion. Peculiarities in meaningful speech may include excessive questioning or preoccupation with some topic at hand.
particular.

3.5

4 Severely abnormal verbal communication: Meaningful speech is not used. The child may emit high-pitched, childlike screams, animal or bizarre sounds, complex noises similar to speech, or may display bizarre and persistent use of a few recognizable words or phrases.

Comments:

XII. NON VERBAL COMUNICATION

1 Normal use of non-verbal communication appropriate for age and situation

1.5

two Slightly abnormal use of non-verbal communication: Immature use of non-verbal communication; the child can only vaguely point or reach for what he wants, in the same situations in which a child of the same age may point or gesture more specifically to indicate what you want.

2.5

3 Use of moderately abnormal non-verbal communication: The child is usually unable to express needs or wants non-verbally, and cannot understand the non-verbal communication from others.

3.5

4 Severely abnormal non-verbal communication use: Child uses only bizarre gestures or peculiar, without apparent meaning, and demonstrates no knowledge of the meanings associated with the gestures or facial expressions of others.

Comments:

XIII. ACTIVITY LEVEL

1 Normal activity level for age and circumstances: The child is neither more nor less active than a normal child of the same age in a similar situation.

1.5

two Mildly abnormal activity level: The child may either be a little restless or a little "lazy", showing, sometimes, slow movements. The child's activity level interferes only slightly with his performance.

2.5

3 Moderately Abnormal Activity Level: The child may be quite active and difficult to contain. She may have boundless energy, or she may not readily go to bed at night. On the other hand, the child may be quite lethargic and need a lot of stimulation to move.

3.5

4 Severely abnormal activity level: Does the child exhibit extremes of activity or inactivity and it can even switch from one extreme to the other.

Comments:

XIV. LEVEL AND CONSISTENCY OF INTELLECTUAL RESPONSE

1 Intelligence is normal and reasonably consistent in several areas: The child is as intelligent as typical children of the same age and does not have any intellectual abilities or problems. unusual.

1.5

two Slightly abnormal intellectual functioning: The child is not as smart as children typical of the same age; skills are fairly even across all areas.

2.5

3 Moderately abnormal intellectual functioning: In general, the child is not as intelligent as a typical child of the same age, however, the child may function close to normal in one or more intellectual areas.

3.5

4 Severely abnormal intellectual functioning: Although the child is usually not as intelligent as a typical child of the same age, he or she may function even better than a normal child of the same age in one or more areas.

Comments:

	XV. GENERAL IMPRESSIONS
**1**	No autism: The child does not have any of the characteristic symptoms of autism.
1.5	
**two**	Mild autism: The child has only a small number of symptoms or only one mild degree of autism.
2.5	
**3**	Moderate autism: The child has many symptoms or a moderate degree of autism.
3.5	
**4**	Severe autism: the child has numerous symptoms or an extreme degree of autism
	**Comments:**

Score by category

**I**	**II**	**III**	**IV**	**V**	**SAW**	**VII**	**VIII**	**IX**	**X**	**XI**	**XII**	**XIII**	**XIV**	**XV**	**Total**

Result:

15-30: no autism

30-36: mild-to-moderate autism

36-60: severe autism

ANNEX 2 - ACSF-SC

Instruções do instrumento ASCF:SC

PASSO 1
Por favor, leia o **Guia do Usuário do Instrumento ACSF:SC** antes de começar.

PASSO 2
Por favor, revise as descrições dos 5 níveis e suas distinções no **Instrumento ACSF:SC**.

PASSO 3
Pensando sobre o último mês, quais são as melhores habilidades de comunicação social que você observou essa criança fazer (mesmo que tenha sido observada apenas uma vez). Isso é chamado Capacidade.

O **Nível de Capacidade** da criança é________________________.

PASSO 4
A seguir revise o instrumento e, novamente, pensando sobre o último mês, quais foram as habilidades de comunicação social que você observou a criança fazendo mais consistentemente? Isso é chamado de Desempenho Típico.

O **Nível de Desempenho Típico** da criança é__________________.

- Os comportamentos de comunicação social da criança no último mês **devem corresponder ou se parecer com a caracterização geral** que é descrita no nível. Se a criança não corresponde à caracterização descrita ela deve ser classificada no nível de habilidade mais baixo.
- Se as habilidades de funcionalidade de comunicação social são **inferiores ao Nível V, classifique-a como V. Da mesma forma, se elas são superiores ao Nível I, classifique-a como I.**

Não se esqueça

Existe uma variedade de maneiras pelas quais as as crianças iniciam comunicação ou respondem à comunicação de outras pessoas, tais como:

expressões faciais, movimentos corporais ou gestos, linguagem de sinais, contato visual e uso de fixação visual para direcionar a atenção de outras pessoas, tecnologia, equipamentos ou ferramentas de CAA (exemplos: PECS, iPad, álbum de fotos, scrapbooks, dispositivos geradores de fala), e fala.

ACSF:SC
INSTRUMENTO

Tradução Português Brasileiro

Sistema de Classificação de Funcionalidade no Autismo: Comunicação Social

Version 2016

Tradução português brasileiro por:
Ana Amélia Cardoso*
Samara Costa, Adriana Queiroz,
Carla Ribeiro Lage, Cecília Pletschette Galvão
*Departamento de Terapia Ocupacional, Universidade Federal de Minas Gerais, (anaameliato@eeffto.ufmg.br)

Por favor, consulte o Guia do Usuário do **ACSF:SC** e as Instruções do **ACSF:SC** antes de revisar os 5 níveis descritos dentro deste folheto.

Autism Classification System of Functioning: Social Communication
ACSF:SC Tool© 2016

Briano Di Rezze, Lonnie Zwaigenbaum, Mary Jo Cooley Hidecker, Martha Cousins, Peter Szatmari, Mary Law, Paul Stratford, Peter Rosenbaum

CanChild Centre for Childhood Disability Research,
McMaster University, Hamilton, ON
www.canchild.ca

Nível V – No último mês, uma criança no nível V pode ter sido observada ...

Brincando com objetos ou falando consigo mesma.

Tentando iniciar ou reagir a palavras ou ações físicas específicas de outra pessoa. O objetivo de sua comunicação pode ser entendido apenas pelo seu cuidador primário ou professor/ terapeuta altamente experimente.

Nível IV – No último mês, uma criança no nível IV tem sido observada ...

Tentando iniciar comunicação com seu(s) cuidador(es) primário(s), solicitando ter suas necessidades atendidas.

Tentando responder a comunicação iniciada por pessoas que ela conhece (pode ser algo tão simples quanto o uso de uma expressão facial), mas pode não estar respondendo a pessoas que ela não conhece.

Nível III - No último mês, uma criança no nível III tem sido observada ...

Iniciando comunicação com pessoas que ela conhece, principalmente para solicitar que tenha suas necessidades atendidas.

Tentando iniciar comunicação com objetivos sociais usando solicitações simples, praticadas ou roteirizadas (verbalmente ou não verbalmente) sobre seus interesses/atividades preferidos.

Respondendo a comunicação de outras pessoas (como quando perguntado sobre questões simples como "O que é isso?"), mas a comunicação não é sustentada.

Nível II - No último mês, uma criança no nível II tem sido observada ...

Iniciando ou respondendo para se comunicar com objetivos sociais sobre seus interesses/ atividades preferidos, com a maioria das pessoas.

Sustentando comunicação até a outra pessoa mudar o assunto/ atividade ou até não estar sendo compreendido.

Nível I - No último mês, uma criança no nível I tem sido observada ...

Iniciando e respondendo para se comunicar com objetivos sociais sobre mais do que somente seus interesses/atividades preferidos, com a maioria das pessoas.

Sustentando comunicação com a maioria das pessoas. Apesar de poder ter alguma dificuldade, ela vai tentar responder à mudança no assunto/ atividade ou usar estratégias efetivas de comunicação para ser compreendida.

Distinções entre os Níveis

Distinção entre nível V e nível IV

Uma criança no Nível V está simplesmente REAGINDO à comunicação de outras pessoas & o objetivo de sua comunicação na melhor das hipóteses é conhecido apenas pelo cuidador primário ou professor/terapeuta altamente experiente,

***enquanto** uma criança no **Nível IV** está TENTANDO iniciar por necessidade própria e TENTANDO responder a pessoas que ela conhece.*

Distinção entre nível IV e nível III

Uma criança no Nível IV pode estar TENTANDO iniciar e responder às pessoas que ela conhece, para ter suas necessidades atendidas,

***equanto** uma criança no **Nível III** está fazendo essas coisas por sua própria necessidade, bem como TENTANDO iniciar a comunicação com objetivos sociais sobre seus interesses preferidos. Ela pode responder à solicitação de outras pessoas, mas a comunicação é roteirizada e não é facilmente sustentada.*

Distinção entre nível III e nível II

Uma criança no nível III está TENTANDO iniciar e responder com objetivos sociais sobre seus interesses/atividades preferidos,

***enquanto** uma criança no **Nível II** está iniciando e respondendo, com objetivos sociais, a maioria das pessoas, que podem continuar a interação. Entretanto, se alguma coisa muda, ou ela não é compreendida, a comunicação não dura.*

Distinção entre nível II e nível I

Uma criança no Nível II está se comunicando com outras pessoas com objetivos sociais, mas tem problemas para sustentar a interação se existirem mudanças,

***enquanto**, embora a interação possa não parecer perfeita, uma criança no **Nível I** tenta sustentar a interação usando estratégias efetivas de comunicação para ser compreendida, e se adaptar a mudanças*

ACSF:SCF Di Rezze, Zwaigenbaum, Cooley Hidecker, et al., 2016
www.canchild.ca

ANNEX 3 -PEM-CY

FOR OFFICE USE ONLY: ____________ (ID)

Participation and Environment Measure – Children and Youth©
(Medida da Participação e do Contexto – Crianças e Jovens)

Wendy Coster, Mary Law, Gary Bedell

Permissão concedida para reproduzir a Medida da Participação e do Contexto—Crianças e Jovens (Participation and Environment Measure—Children and Youth, PEM-CY) em páginas inteiras com informação de copyright, para investigação e prática clínica e não para revenda. Modificações aos itens ou estrutura da PEM-CY, assim como traduções para outros idiomas, não podem ser realizadas sem permissão escrita dos autores.

© Copyright 2010 Trustees of Boston University
Tradução Portuguesa (2012), Susana Martins (susanaisabelmartins@gmail.com) e Manuela Sanches Ferreira, Escola Superior de Educação do Porto, Portugal

INSTRUÇÕES PARA PESQUISA

Participação refere-se ao envolvimento da criança em atividades importantes do quotidiano, em casa, na escola e na comunidade. O significado de participação inclui <u>com que frequência</u> a criança faz as atividades, E o <u>quão envolvida</u> está quando faz essas atividades.

O inquérito coloca uma série de questões acerca da participação da criança em 25 tipos de atividades que ocorrem em três contextos: casa, escola e comunidade. Apresentamos alguns exemplos para ilustrar cada tipo de atividade. No entanto, deve pensar em <u>todas</u> as atividades que pertencem a essa categoria quando responde a essas questões.

Para cada tipo de atividade perguntamos:

1. <u>com que frequência</u> a sua criança participou ao <u>longo dos últimos 4 meses</u>
2. <u>quão envolvida</u> está a sua criança quando participa em <u>1 ou 2 atividades deste tipo que, ele ou ela, faça com mais frequência</u>
3. se gostaria que a participação da sua criança **<u>mudasse</u>** (ou não), se sim, como gostaria que mudasse

IMPORTANTE

Este inquérito não pergunta acerca do nível de independência da sua criança quando participa nas atividades. "Envolvimento" refere-se ao quão empenhada a sua criança está na atividade, usando que apoios, ajudas, adaptações, ou métodos que use regularmente ou que tenha disponível.

Quando selecionar a sua resposta, por favor pense acerca do nível de atenção, concentração, empenho emocional, ou satisfação da sua criança (considerando o uso de suportes ou ajudas que estão geralmente disponíveis).

<u>Muito envolvida</u> = De forma geral, a criança está empenhada durante a atividade. Mostra muita iniciativa e/ou interesse e atenção ao que ele ou ela e outros estão a fazer durante a atividade.

<u>Algo envolvido</u> = A criança está empenhada na atividade durante algum tempo. Mostra alguma iniciativa e/ou interesse e atenção ao que ele ou ela e outros estão a fazer durante a atividade.

<u>Minimamente envolvido</u> = A criança está empenhada uma pequena parte do tempo da atividade. Mostra pouca iniciativa e/ou interesse e atenção ao que ele ou ela e outros estão a fazer durante a atividade.

Se existem aspetos que ajudam ou tornam a participação da sua criança mais difícil, tais como equipamentos ou apoio de outros, pode dizer-nos acerca do seu impacto nas secções de contexto doméstico, contexto escolar e contexto comunitário deste inquérito.

© Copyright 2010 Trustees of Boston University
Tradução Portuguesa (2012), Susana Martins (susanaisabelmartins@gmail.com) e Manuela Sanches Ferreira, Escola Superior de Educação do Porto, Portugal

Participação em CASA

A) Tipicamente, com que <u>frequência</u> a sua criança participa nas <u>atividades apresentadas ou parecidas</u>, dentro de cada uma das categorias abaixo indicadas?

MARQUE UMA RESPOSTA ☑

B) Pense em <u>cada uma das atividades ou parecidas</u>, dentro de cada uma das categorias abaixo indicadas, em que a sua criança participe com mais frequência. Tipicamente, <u>quão envolvida</u> esta a sua criança quando faz essas atividades?

MARQUE UMA RESPOSTA ☑

C) Gostaria que a participação da sua criança <u>mudasse</u> neste tipo de atividade?

SE SIM, MARQUE TODAS AS QUE SE APLICAM ☑

	Diariamente	Algumas vezes por semana	Uma vez por semana	Algumas vezes por mês	Uma vez por mês	Algumas vezes nos últimos 4 meses	Uma vez nos últimos 4 meses	Nunca (saltar para a questão C)	5 Muito Envolvido	4	3 Algo Envolvido	2	1 Minimamente Envolvido	Não desejo mudança	Sim, fazer mais vezes	Sim, fazer menos vezes	Sim, estar mais Envolvido	Sim, estar menos Envolvido	Sim, estar Envolvido numa maior variedade de atividades
1) Jogos de computador e consolas																			
2) Jogos e brincadeiras no interior (ex. brincar com brinquedos, puzzles, jogos de tabuleiro, brincar às cozinhas ou jogos de faz de conta)																			
3) Artes, trabalhos manuais, música e passatempos (ex. fazer trabalhos manuais e de arte, ouvir música, tocar um instrumento, colecionar, ler por prazer, cozinhar por gosto)																			
4) Ver TV, vídeos e DVDs																			
5) Estar com outras pessoas (ex. interagir com pares, familiares, hóspedes)																			
6) Socializar usando tecnologias (ex. telefone, computador)																			
7) Tarefas domésticas (ex. pôr/tirar louça da máquina de lavar louça, limpar o quarto ou outras áreas da casa, cozinhar, levar o lixo, pôr a mesa, cuidar de animal doméstico)																			
8) Cuidados pessoais (ex. vestir-se, escolher a roupa, escovar o cabelo e dentes, colocar maquiagem)																			
9) Preparação para a escola (não trabalho de casa) (ex. reunir materiais, preparar a mochila, colocar lanche na mochila, rever horário)																			
10) Trabalhos de casa (ex. leituras diárias, trabalhos para casa, projectos escolares)																			

© Copyright 2010 Trustees of Boston University
Tradução Portuguesa (2012), Susana Martins (susanaisabelmartins@gmail.com) e Manuela Sanches Ferreira, Escola Superior de Educação do Porto, Portugal

Contexto de CASA

Os seguintes aspetos ajudam ou tornam mais difícil a participação da sua criança em atividades em casa? SELECIONE UMA RESPOSTA ☑	Não é um problema	Geralmente ajuda	Às vezes ajuda; às vezes dificulta	Geralmente torna mais difícil
1. A disposição física ou a quantidade de espaço e mobília em sua casa				
2. As qualidades sensoriais do contexto doméstico (ex. quantidade e/ou tipo de som, luz, temperatura, textura dos objetos)				
3. As exigências físicas das atividades típicas do contexto doméstico (ex. força, resistência, coordenação)				
4. As exigências cognitivas das atividades típicas do contexto doméstico (ex. concentração, atenção, resolução de problemas)				
5. As exigências sociais das atividades típicas do contexto doméstico (ex. comunicação, interação com outros)				
6. O relacionamento da sua criança com os elementos da família em casa (ex. irmãos mais novos, pais, avós)				
7. As atitudes e ações das babysitters, terapeutas e outros profissionais que cuidam da sua criança em contexto doméstico				

SELECIONE UMA RESPOSTA ☑	Não é necessário	Geralmente, sim	Às vezes sim; às vezes não	Geralmente, não
8. Há em sua casa serviços disponíveis e/ou adequados para apoiar a participação da sua criança?				

© Copyright 2010 Trustees of Boston University
Tradução Portuguesa (2012), Susana Martins (susanaisabelmartins@gmail.com) e Manuela Sanches Ferreira, Escola Superior de Educação do Porto, Portugal

Contexto de CASA

Os seguintes estão disponíveis/ ou adequados para apoiar a participação da sua criança em casa? SELECIONE UMA RESPOSTA ☑	Geralmente, sim	Às vezes sim; às vezes não	Geralmente, não
9. Materiais em casa (ex. equipamento desportivo, material de trabalhos manuais, material de leitura, dispositivos de auxílio e tecnologias, horários de imagens ou palavras)			
10. Informação (ex. acerca de atividades, serviços, programas)			
11. Tem (ou a sua família) tempo suficiente para apoiar a participação da criança em casa?			
12. Tem (ou a sua família) dinheiro suficiente para apoiar a participação da criança em casa?			

Quais algumas das coisas que faz, ou outros elementos da família, que ajudam a sua criança a participar com sucesso nas atividades em casa? POR FAVOR LISTE ATÉ 3 ESTRATÉGIAS
1.
2.
3.

© Copyright 2010 Trustees of Boston University
Tradução Portuguesa (2012), Susana Martins (susanaisabelmartins@gmail.com) e Manuela Sanches Ferreira, Escola Superior de Educação do Porto, Portugal

Participação na ESCOLA

A) Tipicamente, com que frequência a sua criança participa nas atividades apresentadas ou parecidas, dentro de cada uma das categorias abaixo indicadas?

MARQUE UMA RESPOSTA ☑

B) Pense em cada uma das atividades ou parecidas, dentro de cada uma das categorias abaixo indicadas, em que a sua criança participe com mais frequência. Tipicamente, quão envolvida está a sua criança quando faz essas atividades?

MARQUE UMA RESPOSTA ☑

C) Gostaria que a participação da sua criança mudasse neste tipo de atividade?

SE SIM, MARQUE TODAS AS QUE SE APLICAM ☑

	A) Diariamente	A) Algumas vezes por semana	A) Uma vez por semana	A) Algumas vezes por mês	A) Uma vez por mês	A) Algumas vezes nos últimos 4 meses	A) Uma vez nos últimos 4 meses	A) Nunca (ir para a questão C)	B) 5 Muito Envolvido	B) 4	B) 3 Algo Envolvido	B) 2	B) 1 Minimamente Envolvido	C) Mudança não desejada	C) Sim, fazer mais vezes	C) Sim, fazer menos vezes	C) Sim, estar mais Envolvido	C) Sim, estar menos Envolvido	C) Sim, estar Envolvido numa maior variedade de atividades
**1) Atividades em sala de aula** (ex. trabalho de grupo, discussões na turma, testes, trabalhos na sala)																			
**2) Excursões e eventos na escola** (ex. ir ao museu, feira escolar, concerto ou peça, danças, angariação de fundos)																			
**3) Equipas dinamizadas pela escola, clubes e organizações** (ex. grupos, clubes, equipas, associação de estudantes)																			
**4) Estar com os colegas fora da sala** (ex. estar com os colegas no almoço, no intervalo ou em outras pausas do dia escolar)																			
**5) Papéis específicos na escola** (ex. tutor de outro aluno, delegado de turma)																			

© Copyright 2010 Trustees of Boston University
Tradução Portuguesa (2012), Susana Martins (susanaisabelmartins@gmail.com) e Manuela Sanches Ferreira, Escola Superior de Educação do Porto, Portugal

Contexto ESCOLAR

Os seguintes aspetos ajudam ou tornam mais difícil a participação da sua criança em atividades na escola? SELECIONE UMA RESPOSTA ☑	Não é um problema	Geralmente ajuda	Às vezes ajuda; às vezes dificulta	Geralmente torna mais difícil
1. A disposição física ou a quantidade de espaço na sala de aula, recreio, ou em outras áreas do edifício escolar (ex. presença de passeios, disponibilidade de rampas ou elevadores no edifício escolar)				
2. As qualidades sensoriais do contexto escolar (ex. barulho, multidões, iluminação, etc.)				
3. Condições atmosféricas exteriores (ex. temperatura, clima)				
4. As exigências físicas das atividades escolares (ex. força, resistência, coordenação)				
5. As exigências cognitivas das atividades típicas do contexto escolar (ex. concentração, atenção, resolução de problemas)				
6. As exigências sociais das atividades típicas do contexto escolar (ex. comunicação, interação com outros)				
7. Atitudes e ações de professores, treinadores ou pessoal da escola face à sua criança				
8. O relacionamento da sua criança com os colegas				
9. A segurança da escola (ex. supervisão, crime, violência)				

Os seguintes estão disponíveis/ ou adequados para apoiar a participação da sua criança na escola? SELECIONE UMA RESPOSTA ☑	Não é necessário	Geralmente, sim	Às vezes sim; às vezes não	Geralmente, não
10. Acesso a transporte pessoal para ir para a escola (ex. carro familiar ou bicicleta)				
11. Acesso a transportes públicos para ir para a escola (ex. autocarro, comboio, metro)				
12. Programas e serviços (ex. depois da escola, recreativos, recursos especiais, ajudas/assistentes educacionais)				
13. Políticas e procedimentos relacionados com a escola (ex. critérios de elegibilidade para serviços, regras de comportamento)				

© Copyright 2010 Trustees of Boston University
Tradução Portuguesa (2012), Susana Martins (susanaisabelmartins@gmail.com) e Manuela Sanches Ferreira, Escola Superior de Educação do Porto, Portugal

Contexto ESCOLAR

Os seguintes estão disponíveis/ ou adequados para apoiar a participação da sua criança na escola? SELECIONE UMA RESPOSTA ☑	Geralmente, sim	Às vezes sim; às vezes não	Geralmente, não
14. Materiais (ex. dispositivos de auxílio ou tecnologias, material de leitura, equipamento desportivo, material de trabalhos manuais)			
15. Informação (ex. acerca de atividades, serviços, programas)			
16. Tem (ou a sua família) tempo suficiente para apoiar a participação da criança na escola?			
17. Tem (ou a sua família) dinheiro suficiente para apoiar a participação da criança na escola?			

Quais algumas das coisas que faz, ou outros elementos da família, que ajudam a sua criança a participar com sucesso nas atividades na escola? POR FAVOR LISTE ATÉ 3 ESTRATÉGIAS
1.
2.
3.

© Copyright 2010 Trustees of Boston University
Tradução Portuguesa (2012), Susana Martins (susanaisabelmartins@gmail.com) e Manuela Sanches Ferreira, Escola Superior de Educação do Porto, Portugal

Participação na COMUNIDADE

A) Tipicamente, com que frequência a sua criança participa nas atividades apresentadas ou parecidas, dentro de cada uma das categorias abaixo indicadas?

MARQUE UMA RESPOSTA ☑

B) Pense em cada uma das atividades ou parecidas, dentro de cada uma das categorias abaixo indicadas, em que a sua criança participe com mais frequência. Tipicamente, quão envolvida está a sua criança quando faz essas atividades?

MARQUE UMA RESPOSTA ☑

C) Gostaria que a participação da sua criança mudasse neste tipo de atividade?

SE SIM, MARQUE TODAS AS QUE SE APLICAM ☑

	A: Diariamente	A: Algumas vezes por semana	A: Uma vez por semana	A: Algumas vezes por mês	A: Uma vez por mês	A: Algumas vezes nos últimos 4 meses	A: Uma vez nos últimos 4 meses	A: Nunca (ir para a questão C)	B: 5 Muito Envolvido	B: 4	B: 3 Algo Envolvido	B: 2	B: 1 Minimamente Envolvido	C: Mudança não desejada	C: Sim, fazer mais vezes	C: Sim, fazer menos vezes	C: Sim, estar mais Envolvido	C: Sim, estar menos Envolvido	C: Sim, estar Envolvido numa maior variedade de atividades
**1) Saídas na vizinhança** (ex. fazer compras na loja/ centro comercial, ir ao cinema, ir ao restaurante, ir à livraria ou biblioteca local)																			
**2) Eventos na comunidade** (ex. ir a uma peça, concerto, eventos desportivos, desfile)																			
**3) Atividades físicas organizadas** (ex. desportos em equipa ou treinos tais como futebol, hóquei, artes marciais, dança, equitação, natação, ginástica)																			
**4) Atividades físicas não estruturadas** (ex. caminhadas na natureza, andar de bicicleta, patins em linha, andar de skate, brincar às escondidas ou apanha, "dar uns toques" com bola)																			
**5) Aulas e cursos (não escolares)** (ex. música, arte, outras línguas, computadores)																			

© Copyright 2010 Trustees of Boston University
Tradução Portuguesa (2012), Susana Martins (susanaisabelmartins@gmail.com) e Manuela Sanches Ferreira, Escola Superior de Educação do Porto, Portugal

Participação na COMUNIDADE

A) Tipicamente, com que frequência a sua criança participa nas atividades apresentadas ou parecidas, dentro de cada uma das categorias abaixo indicadas?
MARQUE UMA RESPOSTA ☑

B) Pense em cada uma das atividades ou parecidas, dentro de cada uma das categorias abaixo indicadas, em que a sua criança participe com mais frequência. Tipicamente, quão envolvida está a sua criança quando faz essas atividades?
MARQUE UMA RESPOSTA ☑

C) Gostaria que a participação da sua criança mudasse neste tipo de atividade?
SE SIM, MARQUE TODAS AS QUE SE APLICAM ☑

	A) Diariamente	A) Algumas vezes por semana	A) Uma vez por semana	A) Algumas vezes por mês	A) Uma vez por mês	A) Algumas vezes nos últimos 4 meses	A) Uma vez nos últimos 4 meses	A) Nunca (ir para a questão C)	B) 5 Muito Envolvido	B) 4	B) 3 Algo Envolvido	B) 2	B) 1 Minimamente Envolvido	C) Mudança não desejada	C) Sim, fazer mais vezes	C) Sim, fazer menos vezes	C) Sim, estar mais Envolvido	C) Sim, estar menos Envolvido	C) Sim, estar Envolvido numa maior variedade de atividades
**6) Organizações, grupos, clubes e atividades de voluntariado ou liderança** (ex. escuteiros, grupos de jovens, associações)																			
**7) Encontros e atividades religiosas ou espirituais** (ex. ir à igreja ou templo, aulas de religião-catequese, grupos)																			
**8) Estar com outras crianças da comunidade** (ex. sair com amigos, encontros informais fora do contexto de casa ou escola)																			
**9) Trabalho remunerado** (ex. babysitting, trabalhar numa loja, fazer tarefas ou recados a troco de dinheiro/ pagamento)																			
**10) Viagens ou visitas em que passa noite fora** (ex. dormir em casa de familiares ou amigos, férias, acampamentos)																			

© Copyright 2010 Trustees of Boston University
Tradução Portuguesa (2012), Susana Martins (susanaisabelmartins@gmail.com) e Manuela Sanches Ferreira, Escola Superior de Educação do Porto, Portugal

Contexto da COMUNIDADE

Os seguintes aspectos ajudam ou tornam mais difícil a participação da sua criança em atividades na comunidade? SELECIONE UMA RESPOSTA ☑	Não é um problema	Geralmente ajuda	Às vezes ajuda; às vezes dificulta	Geralmente torna mais difícil
1. A disposição física ou a quantidade de espaço no exterior e interior dos edifícios (ex. distância até às lojas, existência de passeios, existência de rampas ou elevadores)				
2. As qualidades sensoriais dos contextos comunitários (ex. barulho, multidões, iluminação)				
3. As exigências físicas de atividades típicas (ex. força, resistência, coordenação)				
4. As exigências cognitivas das atividades típicas (ex. concentração, atenção, resolução de problemas)				
5. As exigências sociais das atividades típicas (ex. comunicação, interação com outros)				
6. O relacionamento da sua criança com os colegas				
7. Atitudes e ações de outros membros da comunidade face à sua criança (ex. lojistas, instrutores, treinadores, outros familiares)				
8. Condições atmosféricas exteriores (ex. temperatura, clima)				
9. A segurança da comunidade (ex. tráfico, crime, violência)				

Os seguintes estão disponíveis/ ou adequados para apoiar a participação da sua criança na comunidade? SELECIONE UMA RESPOSTA ☑	Não é necessário	Geralmente, sim	Às vezes sim; às vezes não	Geralmente, não
10. Acesso a transporte pessoal para ir a atividades na comunidade (ex. carro familiar ou bicicleta)				
11. Acesso a transportes públicos para ir a atividades na comunidade (ex. autocarro, comboio, metro)				
12. Programas e serviços (ex. programas de desporto inclusivo, assistentes de apoio pessoal)				

© Copyright 2010 Trustees of Boston University
Tradução Portuguesa (2012), Susana Martins (susanaisabelmartins@gmail.com) e Manuela Sanches Ferreira, Escola Superior de Educação do Porto, Portugal

Contexto da COMUNIDADE

Os seguintes estão disponíveis/ ou adequados para apoiar a participação da sua criança na comunidade? SELECIONE UMA RESPOSTA ☑	Geralmente, sim	Às vezes sim; às vezes não	Geralmente, não
13. Informação (ex. acerca de atividades, serviços, programas)			
14. Equipamentos ou material (ex. equipamento desportivo, material de trabalhos manuais, material de leitura, dispositivos de auxílio ou tecnologias)			
15. Tem (ou a sua família) tempo suficiente para apoiar a participação da criança na comunidade?			
16. Tem (ou a sua família) dinheiro suficiente para apoiar a participação da criança na comunidade?			

Quais algumas das coisas que faz, ou outros elementos da família, que ajudam a sua criança a participar com sucesso nas atividades na comunidade? POR FAVOR LISTE ATÉ 3 ESTRATÉGIAS
1.
2.
3.

© Copyright 2010 Trustees of Boston University
Tradução Portuguesa (2012), Susana Martins (susanaisabelmartins@gmail.com) e Manuela Sanches Ferreira, Escola Superior de Educação do Porto, Portugal

ANNEX 4 - GROSS MOTOR DEVELOPMENT TEST - SECOND EDITION

TGMD-2: Sub teste: Habilidades de locomoção

Habilidade Motora	Materiais	Descrição	Critérios de êxito	1ª	2ª	Escore
Corrida<br>"Vamos correr o mais rápido possível"	18 metros de espaço livre, 2 cones e fita adesiva.	Dois cones (duas linhas) distanciados 15 metros entre si. Dizer à criança para correr o mais rápido possível entre os cones após o sinal.	1. Os braços movem-se em oposição às pernas e com os braços fletidos.			
			2. Existe uma breve fase aérea em que ambos os pés não contactam o solo.			
			3. Não apoia todo o pé simultaneamente. Contacta primeiro no chão com o calcanhar ou a ponta do pé.			
			4. A perna livre flete aproximadamente 90° (perto das nádegas).			
			Escore da habilidade			
Galope<br>"Vamos andar de cavalinho"	8 metros de espaço livre, fita adesiva ou dois cones.	Marcar uma distância de 8 metros com os cones ou a fita adesiva. Dizer à criança para galopar de um cone ao outro. Na segunda tentativa galopar em direção ao 1° cone.	1. Braços fletidos e ao nível da cintura durante a saída do solo.			
			2. Um passo em frente com o pé dominante seguido de um passo do pé não dominante até a uma posição adjacente ou atrás do pé dominante.			
			3. Existe um período de tempo aéreo em que os pés se encontram fora do chão.			
			4. Mantém um padrão rítmico em quatro galopes consecutivos.			
			Escore da habilidade			
Salto com um pé<br>"Vamos pular igual o saci quatro vezes com um perna" (Depois volta com a outra perna)	No mínimo 5 metros de espaço livre.	Dizer à criança para realizar três saltos com o seu pé dominante e depois com o outro pé. Repete duas vezes.	1. A perna livre oscila para frente num movimento pendular para produzir força.			
			2. O pé da perna livre permanece atrás do corpo.			
			3. Braços fletidos oscilando para frente para produzir força.			
			4. Executa três vezes consecutivas com o pé dominante.			
			5. Executa três vezes consecutivas com o pé não dominante.			
			Escore da habilidade			
Passada ou saltar por cima<br>"Vamos correr e saltar o saquinho de areia"	No mínimo 6 metros de espaço livre, um saquinho de areia e fita adesiva.	Colocar o saquinho de areia no chão. Colar uma fita adesiva no chão de modo a que fique paralela e afastada acerca de 3 m do saco de areia. A criança posiciona-se em cima da fita adesiva, corre e salta por cima do saco.	1. Salta num pé e cai com o pé oposto.			
			2. Período aéreo maior do que na corrida normal.			
			3. O braço do lado oposto ao pé de chamada vai à frente no salto.			
			Escore da habilidade			
Salto Horizontal<br>"Vamos saltar o mais longe possível"	No mínimo 3 metros de espaço livre e fita adesiva.	Colocar uma marca de partida no chão. A criança terá de partir atrás da linha. Dizer à criança para saltar o mais longe possível. Repete duas vezes.	1. Movimento preparatório inclui a flexão dos joelhos com os braços estendidos atrás do corpo.			
			2. Braços balançam para frente e para cima atingindo a máxima extensão acima da cabeça.			
			3. Saída do solo e recepção ao solo com ambos os pés simultaneamente.			
			4. Os braços são trazidos para baixo durante a queda.			
			Escore da habilidade			
Deslocamento lateral ou corrida lateral<br>"Vamos correr de lado em cima da linha"	No mínimo 3 metros de espaço livre, uma linha estreita e dois cones.	Colocar os dois cones separados a 7, 5 metros. Dizer à criança para deslocar-se ao longo da linha de um cone ao outro e voltar para trás. Repetir novamente.	1. O corpo permanece lateral de modo que os ombros estão alinhados com a linha do chão.			
			2. Um passo lateral com o pé de apoio do lado do deslocamento seguido de um deslocamento do outro para um ponto próximo do pé.			
			3. No mínimo realiza quatro passos consecutivos para a direita.			
			4. No mínimo realiza quatro passos consecutivos para a esquerda.			
			Escore da habilidade			

TGMD-2: Sub teste: Controle de objetos

Habilidade Motora	Materiais	Descrição	Critérios de êxito	1ª	2ª	Escore
Rebater uma bola "Vamos bater forte na bola" (Deixe a criança escolher qual o lado ela quer fazer)	Bola pequena e leve, bastão de plástico e o suporte da bola	Colocar a bola no suporte ao nível da cintura da criança. Dizer à criança para bater na bola com força. Repetir uma segunda vez	1. A mão dominante segura o bastão acima da mão não dominante.			
			2. O lado não dominante do corpo enfrenta o lançador imaginário com os pés paralelos			
			3. Rotação do tronco (cintura e ombros) durante o movimento			
			4. Transfere o peso do corpo para o pé da frente.			
			5. O bastão contacta a bola			
			Escore da habilidade			
Quicar a bola "Vamos quicar a bola quatro vezes e depois segurar"	Bola com 20 a 25 cm de diâmetro para crianças com 3-5 anos; uma bola de basquetebol para crianças com 6-10 anos	Dizer à criança para quicar a bola quatro vezes consecutivas no mesmo local, usando uma mão e termina agarrando a bola.	1. Contacta a bola com uma mão ao nível da cintura dominante			
			2. Empurra a bola com os dedos (sem bater)			
			3. A bola contacta o solo à frente ou ao lado do pé dominante			
			4. Mantém o controle da bola durante quatro dribles consecutivos sem necessitar de mexer os pés para alcançá-lo.			
			Escore da habilidade			
Receber a bola "Agora você tem que pegar a bola que eu andar para você"	Uma bola de plástico com 10 cm de diâmetro; 5 metros de espaço livre e fita adesiva	Marcar duas linhas com uma distância entre si de 5 metros. A criança fica numa linha e o lançador fica na outra linha. Lançar a bola por baixo diretamente para a criança com um ligeiro arco orientando a para o seu peito. Dizer à criança para agarrar a bola com as duas mãos. A bola deve ser recebida entre os ombros e a cintura da criança.	1. Na fase inicial, as mãos encontram-se à frente do corpo e os cotovelos fletidos.			
			2. Os braços estendem-se para a bola quando esta se aproxima			
			3. A bola é agarrada apenas com as mãos			
			Escore da habilidade			
Chutar a bola "Vamos correr e chutar a bola"	Bola de plástico ou de futebol com 20 a 25 centímetros de diâmetro, saquinho de areia, 10 m de espaço livre e fita adesiva	Marcar uma linha a 10 metros da parede e outra linha a 6 metros da parede. Colocar a bola em cima do saquinho de areia que se encontra na linha mais próxima da parede. Dizer à criança para se colocar na outra linha. Dizer à criança para correr até à bola e chutar com força contra a parede.	1. Aproximação contínua e rápida à bola			
			2. Dá um passo alongado ou pequeno salto antes do contato com a bola			
			3. O pé que não pontapeia deverá ficar ao lado da linha da bola ou ligeiramente atrás da bola.			
			4. Pontapeia a bola com a parte interna do pé dominante ou com os dedos.			
			Escore da habilidade			
Arremesso da bola por cima "Vamos jogar a bola na parede lá no alto"	Bola de tênis, uma parede, fita adesiva, e 6m de espaço livre	Colar uma fita adesiva no chão a 6 metros da parede. A criança deve ficar atrás da linha de frente para a parede. Dizer à criança para lançar a bola com força contra a parede.	1. O giro é iniciado com um movimento da mão/braço para baixo			
			2. Rotação da cintura e dos ombros até o momento em que o lado não lançador se volta para direção do arremesso.			
			3. O peso é transferido como um passo para o pé oposto à mão lançadora			
			4. Movimento contínuo da mão lançadora que cruza diagonalmente para o lado oposto após lançamento.			
			Escore da habilidade			
Arremesso da bola por baixo "Vamos jogar a bola por baixo pra fazer o gol"	Bola de tênis para crianças com 3-6 anos; e uma bola pequena para crianças com 7-10 anos; dois cones; fita adesiva; 8 m de espaço livre	Colocar dois cones contra parede a uma distância entre si de 1,21 m. Colar uma fita adesiva no chão a 6 m da parede. Dizer à criança para rolar a bola com força de modo passar entre os cones.	1. A mão que lança balança para baixo e para trás do tronco enquanto o mesmo está orientado para os cones.			
			2. Passo à frente dado pelo pé oposto à mão que lança			
			3. Dobra os joelhos para se baixar.			
			4. Liberta a bola perto do chão de modo a que a bola não quique mais de 10 cm de altura.			
			Escore da habilidade			

ANNEX 5 - PHYSICAL LITERACY PROFILE QUESTIONNAIRE

QUESTIONÁRIO DO PERFIL DE ALFABETIZAÇÃO FÍSICA

Nome do respondente: ____________ Relação com a criança, adolescente ou adulto jovem: ____________

Nome da criança, adolescente ou adulto jovem: ____________ Idade: ________ Data: ________

▸ PARTE I

▸ Ele (a) utiliza algum dispositivo de tecnologia assistiva (ex.: órtese, andador, muleta, cadeira de rodas, bengalas) ou ajuda de terceiros nas seguintes situações:

Para se locomover dentro de casa? ☐ Sim ☐ Não Tipo de tecnologia ou ajuda de terceiros: ____________

Para se locomover na escola? ☐ Sim ☐ Não Tipo de tecnologia ou ajuda de terceiros: ____________

Para se locomover na comunidade? ☐ Sim ☐ Não Tipo de tecnologia ou ajuda de terceiros: ____________

Para participar de algum esporte/atividade recreativa? ☐ Sim ☐ Não Tipo de tecnologia ou ajuda de terceiros: ____________

Tipo de atividade: ____________ Local: ____________

▸ Ele (a) participa de algum esporte ou atividade recreativa? ☐ Sim ☐ Não Se sim, complete a tabela a seguir:

Esporte/atividade recreativa	Frequência e duração	Local	Características	Tipo de atividade
Exemplo: futebol/queimada/pega-pega	3 vezes por semana, 60 minutos	Clube do bairro	Grama, terreno irregular...	Em grupo, individual

▸ Ele (a) tem interesse em participar de algum esporte ou atividade recreativa? ☐ Sim ☐ Não Qual(is)? ____________

▸ Observações: ____________

QUESTIONÁRIO DO PERFIL DE ALFABETIZAÇÃO FÍSICA - QPAF

▸ PARTE II

▸ Lembre-se que nessa parte do questionário estamos avaliando o desempenho das habilidades e o grau de satisfação. Indique o nível de desempenho (de 0 a 2) e, na última coluna, marque com um X a satisfação com o desempenho dessa habilidade.

Físico					
**Habilidades**<br>No último mês, a criança, adolescente ou adulto jovem realizou atividades esportivas e recreativas:	**0**<br>não realiza	**1**<br>realiza parcialmente	**2**<br>realiza	**A** não sei<br>**B** não teve oportunidade	**Quão satisfeito você está com o desempenho da sua criança/adolescente/adulto jovem nessa habilidade?**
1. Usando habilidades de locomoção (ex.: correr, saltar, propulsão de cadeira de rodas ou qualquer equipamento que permita locomoção)?					1 2 3 4 5 6 7 8 9 10
2. Usando habilidades de manipulação de objetos utilizados na prática esportiva ou recreativa (ex.: arremessar, agarrar e quicar uma bola)?					1 2 3 4 5 6 7 8 9 10
3. Com coordenação (ex.: consegue realizar movimentos dos braços e pernas juntos, como quicar uma bola enquanto se locomove)?					1 2 3 4 5 6 7 8 9 10
4. Com força (ex.: usa o próprio corpo para se puxar, empurrar ou levantar do chão e/ou consegue levantar um objeto pesado, como uma bola pesada)?					1 2 3 4 5 6 7 8 9 10
5. Com agilidade e rapidez (ex.: se movimenta rapidamente para passar uma bola, alcança os colegas em brincadeiras de correr, etc.)?					1 2 3 4 5 6 7 8 9 10
6. Com resistência física (ex.:, consegue participar da brincadeira ou jogo sem ser interrompido pelo cansaço)?					1 2 3 4 5 6 7 8 9 10
7. Com equilíbrio (ex.: participa de brincadeiras ou jogos sem cair com frequência)?					1 2 3 4 5 6 7 8 9 10

▸ Observações: ____________

QUESTIONÁRIO DO PERFIL DE ALFABETIZAÇÃO FÍSICA - QPAF

Social					
**Habilidades**<br>No último mês, a criança, adolescente ou adulto jovem realizou atividades esportivas e recreativas:	**0**<br>não realiza	**1**<br>realiza parcialmente	**2**<br>realiza	**A** não sei<br>**B** não teve oportunidade	**Quão satisfeito você está com o desempenho da sua criança/adolescente/adulto jovem nessa habilidade?**
8. Socializando com os colegas (ex.: interage de forma adequada com os companheiros de equipe)?					1 2 3 4 5 6 7 8 9 10
9. Socializando com adultos (ex.: interage de forma adequada com os profissionais, técnicos, outros pais)?					1 2 3 4 5 6 7 8 9 10
10. Colaborando com os demais (ex.: comunica, coopera e interage com seus colegas e treinador)?					1 2 3 4 5 6 7 8 9 10
11. Com empatia (ex.: demonstra justiça, inclusão e respeito com a equipe)?					1 2 3 4 5 6 7 8 9 10
12. Jogando bem em equipe (ex.: é um 'bom perdedor/ganhador', inclui os outros participantes, respeita a dinâmica da equipe)?					1 2 3 4 5 6 7 8 9 10
**Cognitivo**					
13. Conhecendo diferentes jogos e brincadeiras (ex.: queimada, futebol, etc.) e suas regras gerais (ex.: objetivo principal, forma de pontuação, etc.)?					1 2 3 4 5 6 7 8 9 10
14. Conhecendo estratégias (ex.: possíveis táticas esportivas e maneiras de jogar)?					1 2 3 4 5 6 7 8 9 10
15. Sabendo como se manter segura (ex.: usa capacete em esportes de risco, comporta-se com responsabilidade para não se machucar ou machucar o colega)?					1 2 3 4 5 6 7 8 9 10
16. Sabendo da sua importância (ex.: entende a importância de realizar uma atividade física e/ou sabe o que fazer para permanecer ativo)?					1 2 3 4 5 6 7 8 9 10
17. Reconhecendo e se ajustando a diferentes situações sem instruções (ex.: corre devagar em locais escorregadios ou é gentil com um jogador menor)?					1 2 3 4 5 6 7 8 9 10

Psicológico					
**Habilidades**<br>No último mês, a criança, adolescente ou adulto jovem realizou atividades esportivas e recreativas:	**0**<br>não realiza	**1**<br>realiza parcialmente	**2**<br>realiza	**A** não sei<br>**B** não teve oportunidade	**Quão satisfeito você está com o desempenho da sua criança/adolescente/adulto jovem nessa habilidade?**
18. Com motivação (ex.:demonstrando empolgação para se envolver)?					1 2 3 4 5 6 7 8 9 10
19. Com engajamento (ex.:permanecendo envolvido na atividade)?					1 2 3 4 5 6 7 8 9 10
20. Com confiança (ex.:demonstrando segurança e acreditando que é possível realizar determinada atividade)?					1 2 3 4 5 6 7 8 9 10
21. Com autocontrole (ex.:controla a raiva ou angústia e supera o nervosismo sozinho)?					1 2 3 4 5 6 7 8 9 10
22. Sabendo reconhecer e gerenciar as necessidades físicas (ex.: quando precisa descansar, quando deve parar para tomar água, hora de ir ao banheiro)?					1 2 3 4 5 6 7 8 9 10
23. Conhecendo seus pontos fortes e desafios pessoais (ex.: sabe quais habilidades desempenha muito bem e em quais precisa melhorar)?					1 2 3 4 5 6 7 8 9 10
24. Explorando ambientes para praticar diferentes atividades (ex.: faz atividades diferentes em um ambiente favorito ou pratica uma atividade favorita em diferentes ambientes)?					1 2 3 4 5 6 7 8 9 10

▸PONTUAÇÃO (Espaço reservado aos profissionais de saúde)

POR DOMÍNIO		GERAL
**Domínio Físico**<br>[ ] ÷ 14 = [ ] X 100 [%]<br>Soma dos itens 1-7 — Pontuação total do domínio físico	**Domínio Cognitivo**<br>[ ] ÷ 10 = [ ] X 100 = [ ]<br>Soma dos itens 13-17 — Pontuação total do domínio cognitivo	[ ] ÷ 48 = [ ] X 100 = [ ]<br>Soma dos itens 18-24 — Pontuação geral do QPAF
**Domínio Social**<br>[ ] ÷ 10 = [ ] X 100 = [ ]<br>Soma dos itens 8-12 — Pontuação total do domínio social	**Domínio Psicológico**<br>[ ] ÷ 14 = [ ] X 100 = [ ]<br>Soma dos itens 18-24 — Pontuação total do domínio psicológico	▸ Observações:

ANNEX 6- LETTER OF CONSENT

CARTA DE ANUÊNCIA

Autorizo a utilização das Quadras Abertas de Voleibol e Basquete, de acordo a disponibilidade de agendamento, para a realização do Projeto de Pesquisa ***"Efetividade do Sports Stars Brasil sobre desfechos de atividade, participação e alfabetização física em crianças e adolescentes com transtorno do espectro do autismo: um ensaio controlado randomizado"*** a ser realizado sob orientação do Prof. Dr. Hércules Ribeiro Leite, do Programa de Pós-graduação em Ciências da Reabilitação dos Departamentos de Fisioterapia e Terapia Ocupacional da UFMG, em colaboração com a Profa. Dra. Andressa da Silva de Mello, do Departamentode Esportes.

Belo Horizonte, 26 de abril de 2022.

Profa. Kátia Lúcia Moreira Lemos

Assinado de forma digital por Profa. Kátia Lúcia Moreira Lemos
DN: cn=Profa. Kátia Lúcia Moreira Lemos, o, ou=Universidade Federal de Minas Gerais, email=[illegible], c=BR
Dados: 2022.04.28 09:10:15 -03'00'

APPENDIX A - TERMS OF FREE AND INFORMED CONSENT

TERM OF FREE AND INFORMED CONSENT
(FATHERS, MOTHERS OR GUARDIANS)

Study Title: **Effectiveness of Sports Stars Brasil on activity, participation, and physical literacy outcomes in adolescents with autism spectrum disorder: a randomized controlled trial**

Dear Parents or Guardians,

You and your son are invited to participate in this research coordinated by Professor Dr. Hércules Ribeiro Leite, which aims to investigate the effectiveness of*Sports Stars Brazil,*group physiotherapeutic treatment, centered on carrying out sports activities in children and adolescents with Autism Spectrum Disorder (ASD); and to investigate whether this intervention combined with physical therapy in the context of sports is more effective in facilitating the participation of individuals in sports activities. In order to carry out this research, we need your consent for your child to participate in the study. Your participation in this study will help us investigate whether Sports Stars is an effective treatment for introducing children and adolescents with ASD to sports activities.

After obtaining your consent to participate in this intervention, there will be a draw to see if your child will participate in the Sports Stars program immediately or after the four months of physical therapy treatment that he or she normally undergoes. The Sports Stars program will take place in groups of 3-4 participants of similar ages, once a week, one hour each, for eight consecutive weeks, on the sports courts of the School of Physical Education, Physiotherapy and Occupational Therapy at the Federal University of Minas Gerais. During each weekly session, your child's motor skills will be worked on, through running, jumping, ball activities, as well as introducing the practice of sports: soccer, handball, athletics and basketball.

After your child completes the program*Sports Stars*your child will be sent to perform sports activities, once a week, around an hour, in some program and sport modality of your preference.

After your consent, we will carry out a physiotherapy evaluation with you and your child. You will be asked through a series of questionnaires about your child's characteristics; about how your child participates in everyday activities (at home, at school and in the community); about the main difficulties he/she has in running, jumping and ball activities; and about what you would like your child to do differently to be able to engage in physical and recreational activities. These questionnaires will take around 30 minutes to be answered. Your child will have a physical assessment guided by a trained physical therapist. In this evaluation we will do tests that will evaluate muscle strength, balance and how your child performs running activities, jumping and ball activities. This series of assessments aims to see what your child is capable of doing. Your child's assessments will last approximately one hour with rest periods if needed. If you do not want your child to carry out any of the proposed activities, the test will be interrupted at any time. These assessments and questionnaires will be repeated after repeated after 7 days, 8 weeks and 12 weeks from the first assessment at any time that you and your child are available. If you do not want your child to carry out any of the proposed activities, the test will be interrupted at any time. These assessments and questionnaires will be repeated after repeated after 7 days, 8 weeks and 12 weeks from the first assessment at any time that you and your child are available. If you do not want your child to carry out any of the proposed activities, the test will be interrupted at any time. These assessments and questionnaires will be repeated after repeated after 7 days, 8 weeks and 12 weeks from the first assessment at any time that you and your child are available.

As this is a group dynamic and sports activity training, this intervention, as well as the assessment process, offer a small risk that your child will get tired, fall or be injured during sports activities. . Thus, we will do all activities and exercises and all evaluations with the maximum supervision of a trained professional. In the event of any complications, activities will be interrupted and our team will provide full assistance to you and your child and provide first aid if necessary. All assessments performed with your child will be filmed for test scoring.

You or your child may feel self-conscious during filming. To prevent this from happening, all the details of the procedures for confidentiality of the filming will be previously explained and discussed with you and your child. We emphasize that the videos will only be used to score the test. The videos obtained by filming will be kept in complete secrecy. When answering the questionnaires, during the tests and program activities, you and/or your child may feel uncomfortable or embarrassed with any question or procedure. If this happens, we may interrupt any of the tests or questionnaires, as well as the program's activities, at any time, and your wishes will be respected without any prejudice to you. When answering the questionnaires, during the tests and program activities, you and/or your child may feel uncomfortable or embarrassed with any question or procedure. If this happens, we may interrupt any of the tests or questionnaires, as well as the program's activities, at any time, and your wishes will be respected without any prejudice to you. When answering the questionnaires, during the tests and program activities, you and/or your child may feel uncomfortable or embarrassed with any question or procedure. If this happens, we may interrupt any of the tests or questionnaires, as well as the program's activities, at any time, and your wishes will be respected without any prejudice to you.

To ensure that the information in this study is confidential, information obtained from you and your child will be assigned an identification code upon entry into the study and your child's name will not be disclosed under any circumstances. The data and videos generated in this research will be stored at the School of Physical Education, Physiotherapy and Occupational Therapy at UFMG for 5 years in office 3125 of Professor Dr. Hércules Ribeiro Leite (contact telephone numbers at the end of this document) and will be under his responsibility. If the information originating from the study is published in a journal or scientific event, you and your child will not be identified, always being represented by abbreviations or fictitious names.

For this research, some benefits are expected, among them: the information obtained from the study may contribute to explain the possible benefits of a group physiotherapeutic treatment, centered on the performance of sports activities in children and adolescents with Autistic Spectrum Disorder and the educational strategies that will be offered may favor understanding regarding the functional limitations of the participating children and adolescents, favoring health care and promoting functionality, social participation and physical literacy of children and adolescents with ASD, in different contexts.

Please note that your participation in this research is entirely voluntary and you will not receive any payment or financial compensation for participating. Furthermore, you will not incur any additional expenses for this study. If you have travel expenses for evaluations or to carry out program interventions, our team will be responsible for reimbursing you with the cost of driving in cash (round trip) at each meeting or evaluation moment. It is also important to highlight that you and your child are free to consent to participate in or withdraw from the study at any time. There will be two copies of this document and one copy is intended for you in case of doubt. You can obtain any information about this study from the researchers, and ethical information from the Research Ethics Committee of the Federal University of Minas Gerais (UFMG). Phones are listed below. We will be at your disposal to answer questions or provide clarification on the progress of the work.

If you agree to participate in the study, please sign in the space provided below.

We appreciate your cooperation.

Yours sincerely,

BR

Prof. Hercules Ribeiro Leite
Research Coordinator
Adjunct Professor, Department of Physiotherapy, UFMG

BR

Amanda Cristina Fernandes

Physiotherapist

CONSENT

I,____, responsible for ______________________________________, declare that I have read and understood all the information on the “Effectiveness of Sports Stars Brasil on activity, participation and physical literacy outcomes in children and adolescents with autism spectrum disorder: a randomized controlled trial”, being the clearly explained objectives and procedures. I had enough time to think and choose to participate in the study and I had the opportunity to clarify all my doubts. I am signing this term voluntarily and I have the right, now or later, to discuss any doubts regarding the project.

BR

Parent/guardian signature

Belo Horizonte,_____of_________________of 20___.

Telephone for contact/information:
Professor Dr. Hercules Ribeiro Leite
Department of Physiotherapy, UFMG
Phone:(31) 3409-7404 email: herculesdtnaa@gmail.com

Amanda Cristina Fernandes
Physiotherapist
Phone: (38) 99912-7785 email: amandacristina40@gmail.com

In case of doubts related to ethical issues:
Research Ethics Committee - COEP/UFMG: Av. Pres. Antônio Carlos, 6627 – Administrative Unit II 2nd. Floor – Room 2005 – CEP 31270-901 Belo Horizonte – MG Telephone: (31) 3409-4592. E-mail: coep@prpq @ufmg.br

APPENDIX B - TERMS OF FREE AND INFORMED CONSENT

TERM OF FREE AND INFORMED CONSENT

(FATHERS, MOTHERS OR GUARDIANS)

Study Title: Feasibility and effect of Sports Stars Brasil in adolescents with autism spectrum disorder

Dear Parents or Guardians,

You and your son are invited to participate in this research coordinated by Professor Dr. Hércules Ribeiro Leite, which aims to investigate the effectiveness of*Sports Stars Brazil,*group physiotherapeutic treatment, centered on carrying out sports activities in adolescents with Autism Spectrum Disorder (ASD); and to investigate whether this intervention combined with physical therapy in the context of sports is more effective in facilitating the participation of individuals in sports activities. In order to carry out this research, we need your consent for your child to participate in the study. Your participation in this study will help us investigate whether Sports Stars is an effective treatment for introducing adolescents with ASD to sports activities.

The program*Sports Stars*will take place in groups of 4 participants with similar ages, once a week, one hour each, for eight consecutive weeks, on the sports courts of the School of Physical Education, Physiotherapy and Occupational Therapy of the Federal University of Minas Gerais. During each weekly session, your child's motor skills will be worked on, through running, jumping, ball activities, as well as introducing the practice of sports: soccer, handball, athletics and basketball.

After your child completes the program*Sports Stars*your child will be sent to perform sports activities, once a week, around an hour, in some program and sport modality of your preference.

After your consent, we will carry out a physiotherapy evaluation with you and your child. You will be asked through a series of questionnaires about your child's characteristics; about how your child participates in everyday activities (at home, at school and in the community); about the main difficulties he/she has in running, jumping and ball activities; and about what you would like your child to do differently to be able to engage in physical and recreational activities. These questionnaires will take around 30 minutes to be answered. Your child will have a physical assessment guided by a trained physical therapist. In this evaluation we will do tests that will evaluate muscle strength, balance and how your child performs running activities, jumping and ball activities. This series of assessments aims to see what your child is capable of doing. Your child's assessments will last approximately one hour with rest periods if necessary. If you do not want your child to carry out any of the proposed activities, the test will be interrupted at any time. These assessments and questionnaires will be repeated after 8 and 12 weeks of the first assessment at any time that you and your child are available. the test will stop at any time. These assessments and questionnaires will be repeated after 8 and 12 weeks of the first assessment at any time that you and your child are available. the test will stop at any time. These assessments and questionnaires will be repeated after 8 and 12 weeks of the first assessment at any time that you and your child are available.

As this is group dynamic and sports activity training, this intervention, as well as the assessment process, poses a small risk that your child will tire, fall or be injured during sports activities. Thus, we will do all activities and exercises and all evaluations with the maximum supervision of a trained professional. In the event of any complications, activities will be interrupted and our team will provide full assistance to you and your child and provide first aid if necessary. All assessments performed with your child will be filmed for test scoring. You or your child may feel self-conscious during filming. To prevent this from happening, all the details of the procedures for confidentiality of the filming will be previously explained and discussed with you and your child. We emphasize that the videos will only be used to score the test. The videos obtained by filming will be kept in complete secrecy. When answering the questionnaires, during the tests and program activities, you and/or your child may feel uncomfortable

or embarrassed with any question or procedure. If this happens, we may interrupt any of the tests or questionnaires, as well as the program's activities, at any time, and your wishes will be respected without any prejudice to you. When answering the questionnaires, during the tests and program activities, you and/or your child may feel uncomfortable or embarrassed with any question or procedure. If this happens, we may interrupt any of the tests or questionnaires, as well as the program's activities, at any time, and your wishes will be respected without any prejudice to you. When answering the questionnaires, during the tests and program activities, you and/or your child may feel uncomfortable or embarrassed with any question or procedure. If this happens, we may interrupt any of the tests or questionnaires, as well as the program's activities, at any time, and your wishes will be respected without any prejudice to you.

To ensure that the information in this study is confidential, information obtained from you and your child will be assigned an identification code upon entry into the study and your child's name will not be disclosed under any circumstances.The data and videos generated in this research will be stored at the School of Physical Education, Physiotherapy and Occupational Therapy at UFMG for 5 years in office 3125 of Professor Dr. Hércules Ribeiro Leite (contact telephone numbers at the end of this document) and will be under his responsibility. If the information originating from the study is published in a journal or scientific event, you and your child will not be identified, always being represented by abbreviations or fictitious names.

For this research, some benefits are expected, among them: the information obtained from the study may contribute to explain the possible benefits of a group physiotherapeutic treatment, centered on the performance of sports activities in children and adolescents with Autistic Spectrum Disorder and the educational strategies that will be offered may favor the understanding of the functional limitations of the participating adolescents, favoring health care and promoting functionality, social participation and physical literacy of adolescents with ASD, in different contexts.

Please note that your participation in this research is entirely voluntary and you will not receive any payment or financial compensation for participating. Furthermore, you will not incur any additional expenses for this study. If you have travel expenses for evaluations or to carry out program interventions, our team will be responsible for reimbursing you with the cost of driving in cash (round trip) at each meeting or evaluation moment. It is also important to highlight that you and your child are free to consent to participate in or withdraw from the study at any time. There will be two copies of this document and one copy is intended for you in case of doubt. You can obtain any information about this study from the researchers, and ethical information from the Research Ethics Committee of the Federal University of Minas Gerais (UFMG). Phones are listed below. We will be at your disposal to answer questions or provide clarification on the progress of the work.

If you agree to participate in the study, please sign in the space provided below.

We appreciate your cooperation.

Yours sincerely,

BR

Prof. Hercules Ribeiro Leite

Research Coordinator

Adjunct Professor, Department of Physiotherapy, UFMG

Lidiane Francisca Borges

Physiotherapist

BR

Amanda Cristina Fernandes

Physiotherapist

CONSENT

I, ____, responsible for _____________________________________, declare that I have read and understood all the information about the "Viability and effect of Sports Stars in adolescents with autism spectrum disorder", with the objectives and procedures clearly explained. I had enough time to think and choose to participate in the study and I had the opportunity to clarify all my doubts. I am signing this term voluntarily and I have the right, now or later, to discuss any doubts regarding the project.

BR

Parent/guardian signature

Belo Horizonte,_____of________________of 20___.

Telephone for contact/information:

Professor Dr. Hercules Ribeiro Leite

Department of Physiotherapy, UFMG

Phone: (31) 3409-7404 - E-mail: herculesdtna@gmail.com

Lidiane Francisca Borges Ferreira

Physiotherapist

Phone: (31) 98824-8035 - Email:lidiborges.fisio@gmail.com

Amanda Cristina Fernandes

Physiotherapist

Phone: (38) 99912-7785- E-mail: amandacristina40@gmail.com

In case of doubts related to ethical issues:

Research Ethics Committee - COEP/UFMG: Av. Pres. Antônio Carlos, 6627 –Administrative Unit II 2nd. Floor – Room 2005 – CEP 31270-901 Belo Horizonte – MG Phone: (31) 3409-4592 -E-mail: coep@prpq @ufmg.br

APPENDIX C - TERM OF FREE AND CLEAR ASSENT

TERM OF FREE AND CLARIFIED ASSENT

(CHILDREN 6 YEARS TO 11 YEARS AND 29 DAYS)

Search Title:Effectiveness of Sports Stars Brasil on the participation and physical literacy of children and adolescents with Autism Spectrum Disorder: A randomized controlled trialBR

Hello,

You are invited to participate in this research coordinated by Professor Dr. Hércules Ribeiro Leite who will study about a group physiotherapy treatment called*Sports Stars*BR This treatment focuses on carrying out sports activities for children and adolescents with Autistic Spectrum Disorder (ASD). Our research also wants to find out if the Sports Stars program also works when it is combined with a physiotherapist follow-up while performing sports. To carry out this survey, we need your authorization. Your participation will help us understand whether Sports Stars is a good treatment to encourage children and adolescents with ASD to play sports. The details of this survey are explained below:

ADVENTURE WITH SPORT STARS BRAZIL

HI GUYS, MY NAME IS ANDRÉ AND I HAVE AUTISM, LET'S TALK ABOUT SPORT STARS BRAZIL? THE PROJECT IS A GROUP PHYSIOTHERAPY, WITH UP TO 6 CHILDREN LIKE US.	WELL REMEMBERED ANDRÉ AND THE COOLEST THING IS THAT IT WILL HAPPEN ONCE A WEEK, FOR 1 HOUR, DURING 2 MONTHS. IT WILL BE IN THE QUADRAS OF UFMG. AND DO YOU WANT TO KNOW WHAT OUR PLAYS WILL BE?	LET'S PLAY RUNNING, JUMPING, HANDBALL, FOOTBALL, BASKETBALL AND TRACK AND TRACKBR
NOW LET'S UNDERSTAND HOW THE DYNAMICS WILL BE. TO PARTICIPATE IN THE PROJECT, YOU AND THE DAD OR MOM WILL HAVE TO AGREE AND SIGN.	THERE WILL BE A GIVEAWAY TO DEFINE IF YOU WILL PARTICIPATE NOW OR IN 4 MONTHS.<br>IF YOU ARE DRAWN TO PARTICIPATE 4 MONTHS FROM HERE, YOU WILL CONTINUE CARRYING OUT YOUR DAY TO DAY ACTIVITIES.	THE FATHER OR THE MOM OR THEIR GUARDIAN WILL ANSWER QUESTIONS ABOUT YOUR CHARACTERISTICS, WHAT IS YOUR PARTICIPATION IN DAY TO DAY ACTIVITIES AND WHAT ARE YOUR DIFFICULTIES IN PERFORMING RUNNING, JUMPING AND ACTIVITIES WITH THE BALL. IT WILL TAKE ABOUT 30 MINUTES. AND DURING THESE EVALUATIONS, WE WILL NEED TO MAKE FILMING, RECORD AUDIOS AND TAKE PHOTOS, SO WE WILL FIND OUT WHICH ACTIVITY YOU COULD GAIN MORE POINTS.

WELL, OUR GAMES LAST ABOUT 60 MINUTES. THEREFORE, IF YOU WANT, YOU CAN REST BETWEEN THEM.

AS YOU ALREADY KNOW, WE WILL PLAY RUNNING, JUMPING, KICKING AND OTHER PLAYS. YOU CAN HAPPEN TO GET TIRED, FALL OR INJURE YOURSELF.

BUT DON'T WORRY, ALL YOUR ACTIVITIES AND ASSESSMENTS WILL BE CARRIED OUT WITH A PHYSIOTHERAPIST CLOSE TO YOU, SO YOU CAN PREVENT THIS FROM OCCURING.

BUT IF IT OCCURS, WE HAVE A TEAM OF SUPERHEROES READY TO HELP YOU IN EVERYTHING YOU NEED

A VERY IMPORTANT DETAIL, EVERYONE MUST WEAR THE MASK TO PROTECT THEMSELVES FROM THE CORONA VIRUS

LIKED? THEN COME JOIN WITH US. AND IF YOU ARE LUCKY TO PLAY IN A FEW MONTHS, DON'T WORRY, TIME PASSES FAST AND SOON WE WILL BE TOGETHER.

Source: own author

For this research, some benefits are expected, among them: the information obtained from the study may contribute to explain the possible benefits of a group physiotherapeutic treatment, centered on the performance of sports activities in children and adolescents with Autistic Spectrum Disorder and the educational strategies that will be offered may favor understanding regarding the functional limitations of the participating children and adolescents, favoring health care and promoting functionality, social participation and physical literacy of children and adolescents with autism, in different contexts.

To ensure that the information from this study is kept and that no one can access it, the information obtained from you will receive a code and your name will not be disclosed under any circumstances.The data and videos generated in this research will be stored at the School of Physical Education, Physiotherapy and Occupational Therapy at UFMG for 5 years in office 3125 of Professor Dr. Hércules Ribeiro Leite (contact telephone numbers at the end of this document) and will be under his responsibility. If the information from this study is published in a journal or congress, you will not be identified, and fake codes or names will always be used.

Your participation in this research is voluntary and your parents or guardians will not receive any payment for participating. In addition, you will not incur any expenses with this study. If your parents have expenses with transportation to the evaluations or to carry out the program sessions, our team will be responsible for returning the value of the ticket money (round trip) at each meeting or evaluation moment. It is also important to highlight that you are free to participate or withdraw from the study at any time. A copy of this document is for you in case of doubt. You may obtain any information from this study from the researchers and from the Research Ethics Committee of the Federal University of Minas Gerais (UFMG). Phones are below. If you need to, you can ask questions about the progress of the work.

If you agree to participate in the study, please sign in the space provided below.
We appreciate your cooperation.

Yours sincerely,

BR

Amanda Cristina Fernandes
Physiotherapist

Prof. Hercules Ribeiro Leite
Research Coordinator
Adjunct Professor, Department of Physiotherapy, UFMG

Consent

I,____, responsible for ____, declare that I have read and understood all the information about theBR**Effectiveness of Sports Stars Brasil on activity, participation and physical literacy outcomes in children and adolescents with autism spectrum disorder: a randomized controlled trial**BRobjectives and procedures being clearly explained. I had enough time to think and choose to participate in the study and I had the opportunity to clarify all my doubts. I am signing this term voluntarily and I have the right, now or later, to discuss any doubts regarding the project.

BR
Child's signature or initials

Belo Horizonte,_____of_________________of 20___.

Telephone for contact/information:
Professor Dr. Hercules Ribeiro Leite
Department of Physiotherapy, UFMG
Phone:(31) 3409-7404 email: herculesdtnaa@gmail.com

Amanda Cristina Fernandes
Physiotherapist
Phone: (38) 99912-7785 email: amandacristina40@gmail.com

In case of doubts related to ethical issues:
Research Ethics Committee - COEP/UFMG: Av. Pres. Antônio Carlos, 6627 – Administrative Unit II 2nd. Floor – Room 2005 – CEP 31270-901 Belo Horizonte – MG Telephone: (31) 3409-4592. E-mail: coep@prpq @ufmg.br

APPENDIX D - TERM OF FREE AND CLEAR ASSENT

TERM OF FREE AND CLARIFIED ASSENT

(ADOLESCENTS AGED 12 TO 17 YEARS AND 11 MONTHS)

Study Title: Feasibility and effect of Sports Stars Brasil in adolescents with autism spectrum disorder

Dear teenager,

You are invited to participate in this research coordinated by Professor Dr. Hércules Ribeiro Leite, which aims to investigate the results of a group physiotherapy treatment called*Sports Stars*BR This treatment focuses on carrying out sports activities for adolescents with Autistic Spectrum Disorder (ASD). Our research also wants to find out if this intervention also works when it is combined with a physiotherapist's follow-up during the sports practice of adolescents. To carry out this survey, we need your authorization. Your participation in this research will help us investigate whether Sports Stars is an effective treatment to encourage adolescents with ASD to engage in sports activities.

The program *Sports Stars*will take place in groups of 4-6 participants with similar ages (one group with only teenagers), once a week, one hour each, for eight weeks in the sports courts of the School of Physical Education, Physiotherapy and Occupational Therapy of the Federal University of Minas Gerais General. During each service, you will be taught running, jumping, ball activities and we will also teach you how to play some sports: football, handball, athletics and basketball.

After you and your parents/guardians agree to participate, we will conduct a physical therapist assessment with you and your parents. Your parents or guardians will be asked about your characteristics through an interview; about how you participate in day-to-day activities (at home, at school, and in your neighborhood); what are the main difficulties you have in running, jumping and ball activities; and about what they would like you to do differently to be able to engage in physical and leisure activities. These questionnaires will take around 30 minutes to be answered. You will carry out some tests with a trained physical therapist. In this evaluation, we will observe how your muscle strength is and how you perform running, jumping and ball activities. These tests are for us to see what you are capable of doing. These assessments will last around an hour with rest periods if you need it. If you do not want us to perform any of the activities, we will stop the test at any time. These assessments and interviews will be repeated after 8 and 12 weeks from the first assessment at whatever time you are available.

As we will be doing hectic activities and group sports, this treatment and tests may lead to a small risk of you tiring, falling or injuring yourself during sports activities. Thus, we will do all activities and exercises and all assessments with a physiotherapist very close to you. In case something happens, we will stop the activities and our team will take care of you and we will be responsible for any damage or risk. All assessments performed by you will be filmed for test scoring. You or your parents may feel embarrassed while filming. To prevent this from happening, all the details of what we are going to do with the footage will be explained beforehand. The videos will only be used to observe your tests closely. The videos obtained by filming will be kept saved. When answering the questionnaires, during the tests and program activities, you and/or your parents may feel embarrassed about any question or procedure. If this happens, we will be able to stop any of the tests or questionnaires as well as the program activities, at any time, and your wishes will be respected without any problem.

For this research, some benefits are expected, among them: the information obtained from the study may contribute to explain the possible benefits of a group physiotherapeutic treatment, centered on the performance of sports activities in children and adolescents with Autistic Spectrum Disorder and the educational strategies that will be offered may favor understanding regarding the functional limitations of the participating children and adolescents, favoring health care and promoting functionality, social participation and physical literacy of children and adolescents with autism, in different contexts.

To ensure that the information from this study is kept and that no one can access it, the information obtained from you will receive a code and your name will not be disclosed under any

circumstances.The data and videos generated in this research will be stored at the School of Physical Education, Physiotherapy and Occupational Therapy at UFMG for 5 years in office 3125 of Professor Dr. Hércules Ribeiro Leite (contact telephone numbers at the end of this document) and will be under his responsibility. If the information originating from the study is published in a journal or scientific event, you and your child will not be identified, always being represented by abbreviations or fictitious names.

Your participation in this research is entirely voluntary and you will not receive any payment for participating. In addition, you will not incur any expenses with this study. If your parents have expenses with transportation to the evaluations or to carry out the program sessions, our team will be responsible for returning the value of the transportation in cash (round trip) at each meeting or evaluation moment. It is also important to highlight that you are free to participate or leave the study at any time. A copy of this document is for you in case of doubt. You can obtain any information from this study from the researchers and from the Research Ethics Committee of the Federal University of Minas Gerais (UFMG). Phones are listed below.

If you agree to participate in the study, please sign in the space provided below.

We appreciate your cooperation.

Yours sincerely,

BR

Prof. Hercules Ribeiro Leite

Research Coordinator

Adjunct Professor, Department of Physiotherapy, UFMG

BR

Lidiane Francisca Borges Ferreira

Physiotherapist

BR

Amanda Cristina Fernandes

Physiotherapist

Consent

I,____declare that I have read and understood all the information about the “Feasibility and effect of Sports Stars in adolescents with autism spectrum disorder”, the objectives and procedures being clearly explained. I had enough time to think and choose to participate in the study and I had the opportunity to clarify all my doubts. I am signing this term voluntarily and I have the right, now or later, to discuss any doubts regarding the project.

BR

Adolescent Signature

Belo Horizonte,_____of________________of 20___.

Telephone for contact/information:

Professor Dr. Hercules Ribeiro Leite

Department of Physiotherapy, UFMG

Phone: (31) 3409-7404 - E-mail: herculesdtna@gmail.com

Lidiane Francisca Borges Ferreira

Physiotherapist

Phone: (31) 98824-8035 - Email:lidiborges.fisio@gmail.com

Amanda Cristina Fernandes

Physiotherapist

Phone: (38) 99912-7785- E-mail: amandacristina40@gmail.com

Laboratory for Research & Intervention in Childhood Development and Adolescence (IDEA)

Telephone:(31) 3409-4796

In case of doubts related to ethical issues:

Research Ethics Committee - COEP/UFMG: Av. Pres. Antônio Carlos, 6627 –Administrative Unit II 2nd. Floor – Room 2005 – CEP 31270-901 Belo Horizonte – MG Phone: (31) 3409-4592 -E-mail: coep@prpq @ufmg.br
