# Supplementary material for: Sports Stars Brazil in children with autism spectrum disorder: A feasibility randomized controlled trial protocol
Source: PLoS One. 2023 Nov 8;18(11):e0291488. doi: 10.1371/journal.pone.0291488 (PMC10631688; doi:10.1371/journal.pone.0291488)
Supplement: S5 File — (PDF) [file pone.0291488.s007.pdf]

EFETIVIDADE DO *SPORTS STARS* BRASIL SOBRE DESFECHOS DE ATIVIDADE, PARTICIPAÇÃO E ALFABETIZAÇÃO FÍSICA EM CRIANÇAS E ADOLESCENTES COM TRANSTORNO DO ESPECTRO DO AUTISMO: UM ENSAIO CONTROLADO RANDOMIZADO

Docente responsável:

Prof. Dr. Hércules Ribeiro Leite

Belo Horizonte

2022

RESUMO

O Transtorno do Espectro Austista (TEA) é um transtorno do neurodesenvolvimento caracterizado por dificuldades na comunicação, interação social e comportamentos e/ou interesses repetitivos e/ou restritos. As crianças e adolescentes com TEA podem apresentar comprometimentos no desenvolvimento motor generalizado, como dificuldades de coordenação motora fina/grossa e de equilíbrio. Além disso, este público apresenta risco de inatividade física, obesidade e sobrepeso. As atividades físicas e esportivas são necessárias para aumentar a participação desses indivíduos e, uma maneira de conseguir isso, é promover intervenções que desenvolvam a “alfabetização física” (*physical literacy*). Neste contexto, intervenções focadas no esporte preparam a criança ou o adolescente para a transição das intervenções realizadas individualmente para a participação em esportes e atividades recreacionais na comunidade e, pensando nisso, o *Sports Stars* seria uma ponte para promover esta transição. Apesar dos benefícios reportados na literatura do *Sports Stars* para crianças com paralisia cerebral, ainda não se tem conhecimento dos efeitos do Sports Stars na população com TEA. Nessa perspectiva, o objetivo deste estudo é verificar a efetividade do programa de intervenção *Sports Stars* Brasil em crianças e adolescentes com TEA na melhora dos desfechos de atividade (mobilidade e atividade de vida diária), participação (frequência, envolvimento e responsabilidade) e alfabetização física (competência física, social, cognitiva e psicológica) em comparação com o grupo controle (terapia convencional/lista de espera). Constitui objetivo ainda explorar as experiências dos participantes e de suas famílias que participaram do programa Sports Stars e identificar os resultados percebidos por eles acerca dos domínios da alfabetização física. Este estudo será um estudo controlado randomizado envolvendo aproximadamente 38 crianças com idade entre 6 a 11 anos, 11 meses e 29 dias. Será conduzido ainda um estudo de viabilidade envolvendo 18 adolescentes entre 12 a 18 anos. A amostra será recrutadoa por conveniência através de divulgação em redes sociais pelo Instagram do projeto de extensão *Sports Stars* Brasil. Os participantes serão elegíveis se possuírem idade entre 6 e 18 anos no início da intervenção, com diagnóstico de TEA, classificados de leve a moderado na *Childhood Autism Rating Scale (*CARS), e no nível I e II da *Autism Classification System of Functioning*: Social Communication (ACSF: SC). Os participantes serão excluídos se apresentarem limitações cognitivas, comportamentais ou clínicas (doenças cardiorrespiratórias)

que os impeçam de seguir as instruções e participarem com segurança do programa Sports Stars. Os instrumentos de medidas que serão utilizados para avaliação dos desfechos e caracterização da amostra serão: CARS, ACSF: SC, Perfil Sensorial 2 Abreviado, Medida de Participação e Ambiente para crianças e jovens (PEM-CY), *Goal Attainment Scaling* (GAS), Teste de Desenvolvimento Motor Grosso-2 (TGMD-2), Ignite Challenge, Questionário de Alfabetização Física e *Pediatric Evaluation of Disability Inventory - computer adaptive test* (PEDI-CAT+ASD). Os instrumentos Teste de Desenvolvimento Motor Grosso-2 (TGMD-2), Ignite Challenge, Muscle Power Sprint Test (MPST) e 10×5 Sprint Test (10×5ST) terão sua confiabilidade avaliada para a população de crianças com TEA. Espera-se que o *Sports Stars* apresente eficácia para a melhora dos desfechos reportados acima para a população com TEA, assim como se comprovou ser positiva para crianças e adolescentes com Paralisia Cerebral, ao proporcionar melhora no domínio da alfabetização física, tal como habilidades de locomoção e manipulação, participação em esporte e atividades recreacionais em comparação com o grupo de intervenções usuais.

INTRODUÇÃO

O Transtorno do Espectro do Autismo (TEA) é um transtorno do neurodesenvolvimento caracterizado por dificuldades na comunicação, interação social e comportamentos e/ou interesses repetitivos e/ou restritos (APA, 2014; OMS, 1992) e sintomas estes que configuram o núcleo do transtorno, mas a gravidade de sua apresentação é variável, tendo como causa uma combinação de fatores genéticos e fatores ambientais (SBG, 2019). Segundo a Rede de Monitoramento de Deficiências de Desenvolvimento e Autismo (ADDM), as estimativas de prevalência do TEA estão aumentando, se comparadas às anteriores. Estudos que monitoraram a prevalência do TEA apontam para uma variabilidade considerável entre as diversas populações, com taxa de 18,5 por 1.000, ou seja, 1 a cada 54 crianças possuem TEA (MAENNER et al., 2020; CARLON et al., 2013).

As crianças com TEA geralmente exibem processamento sensorial atípico, como distorções perceptivas, hipo e hiper respostas e preocupações com as características sensoriais dos objetos (ROBERTSON & BARON-COHEN, 2017). O desempenho das habilidades motoras depende de um sistema sensorial intacto, dessa forma, déficits no processamento sensorial podem estar associados a dificuldades na interação com outras pessoas e com o ambiente, bem como no desempenho de habilidades motoras (LIU, 2013).

As crianças e adolescentes com TEA podem apresentar, comprometimentos no desenvolvimento motor generalizado, como dificuldades de coordenação motora fina/grossa e de equilíbrio (CRAIG et al., 2018; OHARA, R et al., 2020). Sabe-se que habilidades motoras prejudicadas impedem a participação das crianças e adolescentes em programas de atividade física em grupo (STINS, J. F., & EMCK, C. 2018). Estudos relatam que a atividade física promove maior independência, interação social, comunicação e melhores habilidades motoras (HUANG, J ET AL, 2020). Além disso, uma condição motora eficaz contribui para o sucesso na participação social em programas de exercícios (SOWA, M., & MEULENBROEK, R. 2012). Apesar dos benefícios relados, crianças e adolescentes com TEA apresentam menores níveis de atividade física quando comparados com seus pares de desenvolvimento típico (MACDONALD ET AL., 2011 ; SRINIVASAN ET AL., 2014 ; STANISH ET AL., 2017; HILLIER ET AL., 2020). Portanto, é necessário estabelecer intervenções que possam aumentar os níveis de atividade física nessa população. Uma forma de promover a participação em atividades físicas recreativas e esportivas é por meio de intervenções que possam desenvolver a "alfabetização física" (do inglês, *physical literacy*) (CARLON et al., 2013).

A "alfabetização física" descreve as competências que uma pessoa precisa para se envolver em atividades físicas agradáveis ao longo da vida, tais como: 1) competência física (por exemplo, capacidade motora grossa); 2) competência psicológica (por exemplo, motivação, engajamento e autorregulação); 3) competência social (por exemplo, relacionamentos e colaboração); e 4) competência cognitiva (por exemplo, conhecimento de conteúdo, conhecimento e raciocínio) (EDWARDS et al., 2017). A participação de crianças e adolescentes em atividades físicas de recreação e esportes requer habilidades em todos esses domínios (EDWARDS et al., 2017; PATEL; SOARES; WELLS, 2017). As habilidades de crianças e adolescentes em todos esses domínios da alfabetização física estimulam sua participação em atividades esportivas e recreacionais (EDWARDS et al., 2017; PATEL; SOARES; WELLS, 2017). Neste contexto, intervenções focadas no esporte, que preparam a criança ou o adolescente para a transição das intervenções individuais usuais para a participação em esportes na comunidade são necessárias (CLUTTERBUCK; AULD; JOHNSTON, 2019a).

Recentemente, Clutterbuck, Auld & Johnston (2018) propuseram um Modelo de Participação Esportiva para crianças e adolescentes com deficiência. O modelo inclui 8 sessões de treinamento de atividades motoras grossas em grupo, com duração de 1 hora, com atividades focadas no esporte e realizadas na comunidade, tendo em vista todos os domínios de alfabetização física 14 (figura 1). Esse modelo auxilia os profissionais de saúde na identificação

de diferentes possibilidades e oportunidades que possam aumentar a participação em atividades físicas e esportivas (CLUTTERBUCK; AULD; JOHNSTON, 2018). Dessa forma, o modelo possui seis fases distintas que representam a progressão que as crianças podem alcançar em termos de participação em atividades físicas ao longo da vida. Intervenções na letra "P", são intervenções de transição de cuidados de saúde individuais para participação em esportes convencionais das fases "ORTS". Projetado para preencher a fase "P" do *Sports Stars* é uma intervenção de esportes modificados liderada por profissionais da saúde, que visa preparar os participantes para a transição dos cuidados usuais de fisioterapia para participação esportiva comunitária (CLUTTERBUCK; AULD; JOHNSTON, 2018). O protocolo *Sports Stars* inclui o treinamento de atividades motoras grossas específicas de esportes culturalmente relevantes, em um contexto projetado para melhorar a confiança, a motivação e as habilidades sociais necessárias para a participação esportiva contínua (CLUTTERBUCK; AULD; JOHNSTON, 2020a). Os efeitos do *Sports Stars* foram investigados em crianças australianas deambulantes com Paralisia Cerebral (PC) e mostraram resultados positivos em metas de participação, atividades motoras grossas e componentes de aptidão física (CLUTTERBUCK; AULD; JOHNSTON, 2020a). Além disso, na percepção dos pais e terapeutas, o Sports Stars melhorou os componentes físicos, sociais, psicológicos e cognitivos da alfabetização física das crianças (CLUTTERBUCK; AULD; JOHNSTON, 2020b).

Atualmente, a efetividade do *Sports Stars* Brasil está sendo investigada em crianças e adolescentes brasileiros com PC (*Sports Stars* Brasil) pelo nosso grupo de pesquisa (DE SOUSA JUNIOR, R. R et al., 2021). Apesar dos benefícios relatados do Sports Stars Austrália, ainda não se tem conhecimento dos efeitos do Sports Stars na população com TEA e de sua aplicação clínica. Dessa forma, este projeto descreve a investigação da intervenção Sports Stars Brasil em crianças e adolescentes com TEA e tem como objetivo: 1) investigar a eficácia do Sports Stars Brasil em comparação com os cuidados fisioterapêuticos usuais; 2) explorar as experiências dos participantes e de suas famílias que participaram do programa Sports Stars e identificar os resultados percebidos por eles acerca dos domínios da alfabetização física; e 3) Verificar as propriedades de medidas dos seguintes instrumentos: Ignite Challenge, Muscle Power, 10x5 Sprint Test e Teste do Desenvolvimento Motor Grosso – segunda edição – TGMD-2 em crianças e adolescentes TEA.

Métodos

O presente projeto será composto por três fases distintas:

1) Investigação da efetividade do *Sports Stars* Brasil na população com TEA.
2) Avaliação das experiências dos participantes e suas famílias.
3) Investigação das propriedades de medidas dos instrumentos Ignite Challenge, Muscle Power, Teste do Desenvolvimento Motor Grosso – segunda edição – TGMD-2, 10x5 Sprint test e Sprint em crianças e adolescentes TEA.

Fase 1: Investigação da efetividade do *Sports Stars* Brasil no TEA

OBJETIVOS

Objetivo Geral

Avaliar a efetividade eficácia do *Sports Stars* Brasil para crianças e adolescentes com TEA, em desfechos de atividade, participação e alfabetização física, em comparação ao grupo controle.

Objetivos específicos:

Avaliar os benefícios do *Sports Stars* Brasil em crianças e adolescentes com TEA sobre desfechos de atividade (mobilidade e atividade de vida diária) e participação (frequência, envolvimento e responsabilidade).

Investigar os benefícios do *Sports Stars* Brasil em crianças e adolescentes com TEA sobre desfechos da alfabetização física (i.e., domínios físicos, cognitivo, psicológico e social).

Hipóteses:

1. Crianças e adolescentes que receberem o programa *Sports Stars* Brasil demonstrarão melhores níveis atividade (mobilidade [e.g., habilidades de locomoção e manipulativas] e atividades de vida diária); e participação (frequência, envolvimento [e.g., atividades esportivas e recreacionais] e responsabilidade) em comparação com aqueles participantes do grupo controle.

2. Crianças e adolescentes que receberem o *Sports Stars* Brasil demonstrarão maiores competências nos domínios da alfabetização física (i.e., domínios físicos, cognitivo, sociais e psicológicos) em comparação com o grupo controle.

MÉTODOS

Design

As crianças participarão de um estudo controlado randomizado desenvolvido de acordo com os Itens do Protocolo Padrão para Ensaios Aleatorizados de Intervenção (*Standard Protocol Items for Randomized Interventional Trials- SPIRIT*) (CHAN; TETZLAFF; ALTMAN, 2016) e será registrado na plataforma de Registro Brasileiro de Ensaios Clínicos (ReBEC). Além disso, também será submetido ao Comitê de Ética em Pesquisa da Universidade Federal de Minas Gerais (UFMG). Dada a dificuldades de recrutamento dos adolescentes neste tipo de estudo, os adolescentes participarão em um estudo de viabilidade.

Ensaio Controlado Randomizado

Aproximadamente 38 crianças com idade entre 6 anos a 11 anos, 11 meses e 29 dias serão recrutados por conveniência através de divulgação em redes sociais pelo Instagram do projeto de extensão Sports Stars Brasil. A definição do tamanho da amostra foi baseada nos resultados originais do Sports Stars (CLUTTERBUCK; AULD; JOHNSTON, 2020a), no qual um tamanho de efeito de 1,05 foi encontrado no desfecho de atividade e participação (Medida Canadense de Desempenho Ocupacional Modificada - COPM) na análise pós-tratamento entre o grupo Sports Stars e o grupo controle. Foram considerados um poder de 80% e α = 95%, bem como uma perda de 20% ao longo do tempo, por meio do software GPower 3.1.

Estudo de viabilidade

Para o estudo com adolescentes, o tamanho da amostra foi calculado de acordo com a equação demonstrada abaixo, que se baseia em critérios de viabilidade inaceitável (zona vermelha - 'STOP') versus viabilidade aceitável (zona verde - 'GO') [Lewis et al., 2021].

$$n = \left(\frac{Z_{1-\alpha}\sqrt{R_{UL}\,(1-R_{UL})} + Z_{1-\beta}\sqrt{G_{LL}(1-G_{LL})}}{(G_{LL} - R_{UL})} \right)^2 + \frac{1}{|G_{LL} - R_{UL}|}$$

Onde: RUL= limite superior da zona vermelha; RUL= limite inferior da zona verde; uma vez que são insuficientes para tal. Nesse caso, eles devem investigar as incertezas sobre Z1- α = probabilidade de erro tipo I; Z1- α = probabilidade de erro tipo II.

Logo, determinando que a taxa de adesão ao estudo é de 65% (zona verde), taxa de falha 35% (zona vermelha), alfa de 5% e poder de 80%, a amostra para viabilidade do estudo seriam 18 indivíduos no total.

Critérios de elegibilidade

Os participantes serão elegíveis para este estudo se possuírem idade entre 6 e 18 anos no início da intervenção, com diagnóstico de TEA, classificados como leves a moderado na Escala *Childhood Autism Rating Scale* (CARS) (PEREIRA; RIESGO; WAGNER, 2008) e classificados por nível I e II *Autism Classification System of Functioning: Social Communication* (ACSF): SC (anexo 2) (ELOI, et al., 2019). Os participantes serão excluídos se apresentarem limitações cognitivas, comportamentais ou clínicas (doenças cardiorrespiratórias) que os impeçam de seguir as instruções e participar com segurança da atividade física em um ambiente de grupo.

PROCEDIMENTOS DE INTERVENÇÃO

Randomização e cegamento

Crianças (n: 36) serão randomizados em dois grupos: intervenção *Sports Stars* Brasil e grupo controle, que receberão terapia usual (fisioterapia e/ou terapia ocupacional) ou lista de espera. A randomização ocorrerá em blocos, com cada randomização ocorrendo quando forem recrutados dois subgrupos de 4-5 participantes na faixa etária da criança ou dos adolescentes. Um gerador de números aleatórios será usado para criar uma sequência aleatória de números. Esses números serão ocultados em envelopes selados opacos numerados individualmente. Essa sequência será usada para randomizar crianças no grupo *Sports Stars* Brasil ou no grupo controle. Uma nova sequência será usada para cada randomização de subgrupo até que as 38 crianças sejam alocados ou outros participantes não possam ser recrutados. Os mesmos procedimentos serão realizados com os adolescentes. Os avaliadores serão cegados quanto à alocação dos grupos. Devido às características da intervenção deste estudo, não é possível garantir cegamento dos indivíduos e terapeutas de intervenção.

<u>Grupo de intervenção *Sports Stars* Brasil:</u> Esta intervenção em grupo terá a participação de quatro a cinco indivíduos em cada grupo, que será liderado por um fisioterapeuta, mas contará com a assistência de profissionais da terapia ocupacional e educação física. A intervenção ocorrerá ao longo de oito semanas, com sessões de 1 vez na semana semanais, com duração de

1 hora. Em cada sessão serão treinadas nesta ordem: treino de habilidades motoras grossas relacionadas à prática esportiva (corrida, salto, atividades com bola). Depois disso, os participantes desse grupo serão apresentados a esportes populares no Brasil: futebol, handebol, basquete e atletismo (tabela 1). Essa intervenção será realizada em ambientes esportivos.

			**Habilidades**	**Motoras Grossas**		
	**Início**	**Aquecimento**	**Locomotor**	**Controle de objetos**	**Desaquecimento**	**Esfriamento**
**Tempo**	10 min	5 min	15 min	15 min	10 min	5 min
**Atividades**	Chegada dos participantes e familiares	Movimentação ativa	Habilidades avançadas (corrida, saltos). Agilidade e coordenação	Pegando e Jogando Chutando e recebendo Manipulação com bolas	Experiência em esportes modificados (futebol, handebol, basquete e atletismo)	Equilíbrio Alongamento Diminuição da frequência cardíaca

Tabela 1. Caracterização das sessões do Sports Stars.

Uma vez que a população TEA podem apresentar comportamentos repetitivos e estereotipados, irritabilidade, agressão, hiperatividade, desatenção e prejuízo social (DOYLE, C.A. & MCDOUGLE, C.J. 2012), estratégias baseadas na *Applied Behavior Analysis* ABA serão utilizadas como facilitadoras para a realização do treinamento das habilidades motoras. As estratégias baseadas na ABA têm demonstrado benefícios ao atenuar esses comportamentos do TEA e promover uma variedade de habilidades sociais, de comunicação e comportamentos adaptativos (CAMARGO; RISPOLI, 2013). Para tal, as crianças serão avaliadas quanto ao seu perfil sensorial, onde serão identificados possíveis déficits de integração sensorial, tanto hipo ou hiper-atividade. Crianças e adolescentes com TEA com perfil sensorial hiporreativo podem apresentar alto limiar neurológico de excitação e precisam, por conseguinte, de *inputs* sensoriais intensos, para que sejam registrados e provoquem motivação para a ação. Esses indivíduos normalmente são passivos, com bom comportamento, silenciosos, e podem não se envolver facilmente nas atividades. As estratégias de intervenção, nos casos de hiper-reatividade, consistem em manter o nível de alerta adequado, para que a criança não adote atitude de fuga, luta ou medo, diante de estímulos que estejam além da sua capacidade de tolerar (ANDRADE, M. M. A. D. 2020). Por meio do perfil sensorial, as estratégias da ABA serão fornecidas para aos participantes, através de profissionais capacitados.

Grupo Controle - cuidados usais ou lista de espera: Os participantes desse grupo manterão sua intervenção de terapia ocupacional e/ou fisioterapia convencional, ou permanecerão em lista de espera. Acredita-se que, no Brasil, crianças e adolescentes com TEA recebam, em média, de uma a duas sessões de terapia individual semanal em clínicas públicas ou privadas. Todas as atividades terapêuticas realizadas pelos participantes do grupo de espera serão registradas em um diário de atividades.

PROCEDIMENTOS E COLETA DE DADOS

Características dos participantes

A idade, sexo e classificação dos instrumentos da *Childhood Autism Rating Scale* (CARS-BR) (anexo 1), *Autism Classification System of Functioning: Social Communication* (ACSF: SC) (anexo 2) e Perfil Sensorial 2 Abreviado serão coletados após a assinatura dos termos de consentimento livre esclarecido (TCLE) (apêndice A e B) assinados pelos pais ou responsáveis e assentimento livre esclarecido (TALE) (apêndice C e D) assinado pelas crianças ou adolescentes participantes.

Childhood Autism Rating Scale (CARS-BR)

A escala CARS-BR é um instrumento traduzido para o Português-Brasil, confiável e validado para a população brasileira com TEA de 3 a 17 anos, apresentando boa consistência interna, validade discriminativa, validade convergente e confiabilidade teste-reteste de 0,90 (PEREIRA; RIESGO; WAGNER, 2008). A CARS-BR é utilizada em diagnósticos e pesquisas relacionadas ao TEA, além de contribuir para distinguir crianças e adolescentes com outros atrasos do desenvolvimento. É dividida em 15 itens, em que avaliam o comportamento em 14 domínios geralmente característicos do TEA e um domínio de escore diagnóstico de gravidade geral, baseados em uma escala de gravidade em quatro pontos (transtorno ausente, leve a moderado ou grave), a pontuação varia de 15 a 60, e o ponto de corte para autismo é 30 pontos (RAPIN; GOLDMAN, 2008; PEREIRA; RIESGO; WAGNER, 2008).

Autism Classification System of Functioning: Social Communication (ACSF: SC)

ACSF:SC é um sistema de classificação que tem como objetivo coletar informações, de modo simplificado e estandardizado, válido e confiável, para caracterizar em níveis as habilidades de comunicação de crianças com TEA, para crianças de 3 a 6 anos (DI REZZE et al. 2016). O instrumento foi adaptado para a crianças com TEA e garantiu equivalência entre a versão

original e traduzida, obtendo índices de concordância acima de 90%. A classificação é dividida em 5 níveis que distinguem as habilidades de comunicação social da criança segundo as necessidades e objetivos sociais (nível V corresponde a quando a criança está reagindo à comunicação de outras pessoas e o objetivo é conhecido apenas por seus pais ou cuidadores principais; nível IV é quando a criança está tentando iniciar por necessidade própria e tentando responder as pessoas que ela conhece; nível III a criança tenta iniciar e responder com objetivos sociais sobre seus interesses; nível II a criança se comunica com outras pessoas, mas apresentam dificuldades se ocorrerem mudanças; nível I a criança sustenta interação e se adapta às mudanças), A classificação final é obtida com base em informações relatadas por pais e profissionais familiarizados com as habilidades de comunicação social da criança, quanto maior o nível, melhor o desempenho da comunicação social e habilidades de comunicação (ELOI, et al., 2019). O instrumento para a população TEA acima de 6 anos de idade está em processo de tradução por uma pesquisadora deste grupo de pesquisa e será utilizado após finalizada esta etapa.

Perfil Sensorial 2 Abreviado

O Perfil Sensorial 2 Abreviado é um questionário destinado a contribuir para a avaliação do desempenho sensorial de crianças e adolescentes de 3 a 14 anos e 11 meses composto por 34 itens, que são respondidos pelo cuidador sobre a frequência com que os comportamentos ocorrem no cotidiano. É dividido em uma a combinação de pontuações do sistema sensorial (audição, visão, tato, movimento e atenção), comportamental (comportamental, conduta, socioemocional e atenção) e padrão sensorial (exploração, esquiva, sensibilidade e observação) (DUNN, 2014). O perfil sensorial 2 Abreviado corresponde à segunda edição do instrumento Perfil Sensorial (DUNN, 1999), é composto por cinco questionários (Perfil Sensorial 2 do Bebê, Perfil Sensorial 2 da Criança Pequena, Perfil Sensorial 2 da Criança, Perfil Sensorial 2 Abreviado, Perfil Sensorial 2 de Acompanhamento Escolar), sendo selecionados, para este trabalho, o perfil sensorial da criança (3 anos a 14 anos e onze meses) (DUNN, 2017). É um questionário baseado em julgamento e deve ser aplicado a quem tem um contato diário com a criança/adolescente e seja por ela responsável. Cada questão descreve as respostas das crianças às várias experiências sensoriais. As respostas deverão considerar com qual frequência (sempre, frequentemente, ocasionalmente, raramente, nunca) ocorrem os comportamentos, e as pontuações são dadas de 1 a 5 (1 para "sempre" e 5 para "nunca"). Quanto menor a pontuação, mais indícios de dificuldades sensoriais, ou seja, pontuações mais baixas indicam maior

gravidade de problemas sensoriais, e, quanto maior a pontuação, menos indícios de dificuldades sensoriais.

Medidas de desfecho

As medidas de desfecho ocorrerão no início do estudo (fase 1-linha de base), após 8 semanas de intervenção (fase 2 - follow-up) e após 12 semanas do início da intervenção (fase 3 – follow-up) por dois avaliadores cegados. Antes da avaliação inicial, os avaliadores participarão de capacitações com a equipe do estudo, para treinamento dos instrumentos que serão usados neste estudo. A confiabilidade dos avaliadores realizada por meio da aplicação dos testes em um mínimo de 10 crianças e adolescentes típicas ou com TEA, bem como com seus pais ou cuidadores, quando necessário.

Desfechos primários:

***Goal Attainment Scaling* (GAS)**

A GAS é uma escala funcional para quantificar o alcance ou cumprimento de metas previamente definidas em um programa de intervenção. (KIRESUK E SHERMAN, 1968; TURNER – STOKES, 2003). Segundo manuais elaborados por Turner - Stokes (2003), o procedimento para a aplicação da GAS consiste em cinco passos: definição dos objetivos terapêuticos; graduação dos objetivos referente a importância e dificuldade; definição dos resultados esperados com a intervenção; classificação dos resultados esperados (em cinco níveis: -2, -1, 0, +1 e +2); cálculo do escore da GAS (por meio de uma fórmula desenvolvida por Kiresuk & Sherman (1968), com descrição disponível em Turner – Stokes, 2003). Nessa escala, a criança e/ou sua família classificam as metas e prioridades em uma escala com escores de 0 a 3. Zero significa nenhuma importância ou dificuldade e 3, importância ou dificuldade máximas. Caso o paciente classifique algum objetivo como 0, esse objetivo deverá ser renegociado e substituído por outro de maior prioridade. Para este estudo, três objetivos serão identificados. O primeiro se relaciona ao desempenho de uma atividade motora grossa avançada, específica para esportes (por exemplo, correr, chutar, arremessar ou pular), o segundo se refere ao comparecimento a uma recreação física ou atividade esportiva (por exemplo, assistir a um jogo de futebol com amigos duas vezes por semana) e o terceiro se refere ao envolvimento durante uma recreação física ou atividade esportiva (por exemplo, estar motivado

ao participar de um jogo de futebol com os amigos). Esses objetivos serão selecionados e classificados pelos pais ou responsáveis juntamente com o participante e terapeuta responsável.

Medida de Participação e Ambiente para crianças e jovens (PEM-CY)

A PEM-CY (anexo 3) é um instrumento que avalia a participação e ambiente de crianças e adolescentes com deficiência, em seus ambientes doméstico, escolar e na comunidade (COSTER et al., 2011, 2012; GALVÃO et al., 2018). É uma das poucas avaliações que combina medida de participação e ambiente para crianças e jovens. É uma ferramenta que avalia a participação e o ambiente de crianças e jovens, com idade entre 5 e 17 anos, segundo a percepção dos pais ou cuidadores. A medida identifica o envolvimento das crianças e jovens em atividades realizadas em casa, na escola e na comunidade, bem como as característi cas destes ambientes que influenciam na participação (BEDELL, G.M. et al). A PEM-CY foi traduzida e validada para a população brasileira com a finalidade de mensurar de forma objetiva o domínio da participação em crianças (GALVÃO et al., 2018). Este questionário compreende seis partes diferentes: frequência de participação, envolvimento de participação, desejo de mudança de participação, apoio ao meio ambiente, apoio ao meio ambiente e recursos ambientais e cada subescala é pontuado em diferentes intervalos e convertido em porcentagens (0% -100%). Uma pontuação total pode ser calculada somando as porcentagens de todas as dimensões. (COSTER et al., 2011, 2012; GALVÃO et al., 2018). Apresenta índices de consistência interna e confiabilidade teste-reteste de moderado a boa (COSTER et al., 2011) e foi traduzido e adaptado culturalmente para a população brasileira por Galvão et al. (2018). A diferença clinicamente significativa para o PEM-CY ainda não foi determinada, mas tem boa confiabilidade intra e interexaminador e tem sido usado como uma medida de desfecho para avaliar os efeitos da participação intervenções em indivíduos com TEA (SIMPSON, K et al.2019; KRIEGER, B et al 2020; WILLIAMS, K et al. 2021).

Teste de Desenvolvimento Motor Grosso-2 – TGMD-2

O TGMD-2 (anexo 4) é um teste para avaliação do desenvolvimento motor grosso de meninos e meninas entre três e 10 anos. O teste avalia 12 habilidades motoras fundamentais, das quais seis são habilidades de locomoção (correr, galopar, saltitar, dar uma passada, saltar horizontalmente e correr lateralmente) e seis são habilidades de controle de objetos (rebater, quicar, receber, chutar, arremessar por cima do ombro e rolar uma bola). Para cada habilidade são observados de 3 a 5 critérios motores específicos. O TGMD-2 permite uma avaliação separada de cada subteste (locomoção e controle de objeto) e ainda no subteste de controle de

objeto, uma diferenciação por gênero. Os escores totais de cada subteste são somados e representados como escores brutos, que podem ser convertidos em quocientes motores (ULRICH, 2000). O TGMD-2 foi validado e confiável para crianças brasileiras no estudo de Valentini et al., (2012) e tem sido utilizado em crianças com TEA (KRUGER, SILVEIRA; MARQUES, 2019; MOHD NORDIN, A; ISMAIL, J & KAMAL NOR, N, 2021). O TGMD-2 possui excelentes índices de confiabilidade e validade (CAPIO; SIT; ABERNETHY, 2011a, 2011b). Entretanto, não apresenta valores de confiabilidade para população TEA.

Ignite Challenge

O *Ignite Challenge* foi criado como um desdobramento do teste avançado *Challenge* de 25 itens para crianças ambulatoriais independentes com PC (ARBOR-NICITOPOULOS ET AL., 2018; 2021). O *Ignite Challenge* tem dois componentes principais: 1) avaliação da qualidade e dos aspectos das habilidades locomotoras e de controle de objetos e 2) apresenta uma abordagem de avaliação dinâmica que poderá aumentar o desejo da criança de permanecer envolvida no teste, acreditamos que o teste terá um forte potencial para crianças com TEA. Uma adaptação posterior do *Ignite Challenge* refinou seu processo de administração para uso com crianças com ASD. *Ignite Challenge* incluiu um novo processo de introdução que poderia incluir uma história social, item 'cartões de imagem' (ALLEN ET AL., 2017; LIU & BRESLIN, 2013) para acompanhar as demonstrações imitativas do avaliador e permitir que a criança escolha qual item fazer a seguir (BRESLIN & RUDISILL, 2011), reduzindo a duração para duas (em vez de três) tentativas para cada item e maior ênfase no uso de dicas verbais diretas e simplificadas. O '*Ignite Challenge* é uma medida baseada no desempenho de 13 itens, sendo projetada para uso com crianças com transtorno do espectro do autismo (ASD) que têm 6 anos ou mais, que são capazes de fazer um teste de habilidades de movimento de 45-60 minutos (incluindo intervalos) sob a orientação do avaliador, sem a orientação dos pais ou responsáveis. Contudo, ainda não é um teste que foi validado para a população brasileira.

Questionário Perfil de Alfabetização Física (anexo 5)

O questionário mede o nível de alfabetização física das crianças, adolescentes e adultos jovens (6-21 anos de idade). Foi desenvolvido para identificar metas e objetivos para as intervenções relacionadas ao esporte e atividades recreativas. Sendo composto por duas partes e deve ser respondido pelos cuidadores, mas as crianças, adolescentes e adultos jovens podem estar presentes no momento da aplicação e auxiliarem no esclarecimento de dúvidas. As perguntas são relacionadas à experiência pessoal em atividades esportivas (atividades físicas com

competição e regras formais) e recreativas (atividades físicas livres objetivando saúde, bem estar e diversão como, por exemplo, pega-pega, queimada, esconde-esconde, entre outras) e ao desempenho (como realiza a atividade) e satisfação nos domínios: físico, social, psicológico e cognitivo. Cada item avalia o desempenho em uma escala de 0 a 2 e cada item avalia a satisfação quanto ao desempenho da criança/adolescente/adulto jovem em uma escala de 1-10 pontos. O questionário de alfabetização física está em processo de validação por este grupo de pesquisa e pode ser excluído deste estudo se for as propriedades psicométricas não apresentarem consistência para aplicação.

***Pediatric Evaluation of Disability Inventory - computer adaptive test* (PEDI-CAT)**

O PEDI-CAT foi desenvolvido para medir o desempenho em atividades diárias, mobilidade, cognitivo-social e responsabilidade em crianças e adolescentes até 21 anos de idade (HALEY, S.M. et al 2011). Sua aplicação exige um computador com o software do instrumento instalado e pode ser auto administrado (ou seja, preenchido pelos pais da criança), ou com a presença de um profissional treinado para garantir o entendimento correto das informações de cada item (HALEY, S.M. et al 2011; HALEY, S.M. et al 2011). O PEDI-CAT inclui domínios que se alinham com os domínios de 'atividade' da Classificação Internacional de Funcionalidade, Incapacidade e Saúde para Crianças e Jovens (CIF-CY) (ou seja, como a criança realiza atividades em seu ambiente diário normal) e 'participação' (é o envolvimento de um em tarefas da vida diária). Nos domínios das atividades diárias, mobilidade e social cognitiva, as pontuações de quatro pontos baseiam-se em diferentes níveis de dificuldade. O domínio responsabilidade classifica os itens em uma escala de cinco pontos, descrevendo a divisão da responsabilidade entre o cuidador e a criança ou adolescente no gerenciamento de tarefas de vida complexas e em várias etapas. A pontuação geral é transformada em uma pontuação normativa (com base na idade) e uma pontuação contínua que será usada nas análises. O PEDI-CAT+ASD inclui instruções adicionais para ajudar os pais a selecionar uma classificação apropriada, dadas as características únicas das crianças com autismo, inclui itens novos ou revisados nos domínios atividades diárias, sociais / cognitivos e responsabilidade. A escala de itens no domínio social / cognitivo foi ajustada de acordo com os padrões únicos de crianças e jovens com autismo, mas as pontuações são expressas na mesma métrica do PEDI-CAT original. Os outros domínios não requerem nenhum ajuste de pontuação para comparações entre as versões. O PEDI-CAT foi adaptado e apresenta confiabilidade para ser usado por crianças e jovens com autismo (KRAMER, J.M et al 2012). Foi traduzido e adaptado culturalmente para a população brasileira de 0-21 anos de idade (MANCINI, M. C. et al. 2016).

O Muscle Power Sprint Test (MPST) é um teste de campo simples para avaliar o desempenho anaeróbico em crianças e adolescentes. O participante é solicitado a correr o mais rápido possível por 15 m (marcados por linhas e cones), 6 vezes, com intervalo de 10 segundos entre cada sprint. Leva alguns minutos para ser concluído e requer apenas um espaço aberto, um cronômetro e dois cones. O MPST tem alta confiabilidade interobservador e teste-reteste (r = 0,97–0,99) para crianças com PC que andam (VERSCHUREN O, TAKKEN T, KETELAAR M, GORTER J.W, HOLDERS P.J.M, 2007). As propriedades em crianças e adolescentes com TEA serão estudadas neste estudo.

O 10×5 Sprint Test (10×5ST) avalia a agilidade e a capacidade anaeróbica em crianças ambulantes com PC. Neste teste, os participantes precisam correr 5 m separados por 2 cones, 10 vezes continuamente, dando voltas nos cones que marcam o final dos cinco metros (VERSCHUREN, TAKKEN, KETELAAR, GORTER, HELDERS, 2007). O Teste Sprint 10 × 5 Metros apresenta excelente confiabilidade interobservador (CCI > 0,97) e teste-reteste (r = 1), além de boa validade de construto relatada. Um aumento no tempo de exercício de 3,2 segundos é considerado uma mudança real (VERSCHUREN, TAKKEN, KETELAAR, GORTER, HELDERS, 2007). No entanto, as propriedades psicométricas ainda não foram estudadas na população de TEA, por isso serão investigadas neste estudo.

Para os adolescentes participantes e seus responsáveis, além dos instrumentos supracitados, serão coletadas informações quanto a viabilidade do estudo: aderência da intervenção, satisfação com a intervenção, dificuldade de entendimento quanto a intervenção ofertada. Estas medidas são coletadas em um questionário semiestruturado (link: https://docs.google.com/forms/d/13ltHY0YXWBJQ5o8pey5K129P_qeTA4oRy_cXdG-QQJo/edit?usp=sharing).

ANÁLISE DE DADOS

A análise estatística descritiva será realizada para as características dos participantes (idade, sexo, classificação CARS e ACSF: SC). Os dados serão analisados para os grupos e separadamente para as crianças (6-11 anos, 11 meses e 29 dias anos) e adolescentes (12-18 anos). A normalidade dos dados será investigada usando o teste Shapiro-Wilk o teste de Levene para verificar a homogeneidade da variância. Os dados contínuos serão relatados usando média e desvio padrão (ou mediana e interquartil, se não forem normalmente distribuídos) e os dados categóricos serão relatados usando distribuições de frequência. Modelos lineares mistos serão

utilizados para avaliar os efeitos do *Sports Stars* Brasil em comparação com o tratamento padrão em todos os resultados. Será realizado o teste t (variáveis paramétricas) ou Mann-Whitney (variáveis não paramétricas) para amostras independentes para comparação entre os grupos, bem como a magnitude do efeito e o poder estatístico. Será adotado um nível de significância de 5% ($\alpha < 0,05$). Para determinar a confiabilidade dos examinadores, serão avaliados os coeficientes de confiabilidade (coeficiente de correlação intraclasse [ICC] ou kappa [k]), sendo considerados satisfatórios aqueles com valores superiores a 0,70. As análises estatísticas serão realizadas com o software Statistical Package for the Social Sciences (SPSS).

RISCOS E BENÉFICIOS

Riscos:

1) Risco de cansaço ou quedas durante as atividades esportivas, para minimizar potenciais riscos, haverá sempre a supervisão máxima de um profissional treinado ao lado da criança ou adolescente e em caso de fadiga ou cansaço, as atividades poderão ser interrompidas para uma pausa.

2) Risco de constrangimento durante a filmagem dos testes, para minimizar os riscos de constrangimento, os vídeos obtidos pelas filmagens serão mantidos em completo sigilo;

3) Risco de desconforto ou constrangimento em responder os questionários. Caso isso aconteça qualquer um dos testes ou questionários bem como as atividades do programa, poderão ser interrompidas em qualquer momento.

Benefícios:

1) As informações advindas do estudo poderão contribuir para a elucidação dos possíveis benefícios de um tratamento fisioterapêutico em grupo, centrado na realização de atividades esportivas em crianças e adolescentes com Transtorno do Espectro Autista (TEA);

2) As estratégias educativas que serão oferecidas poderão favorecer o entendimento dos pais a respeito das limitações funcionais de seus filhos, otimizando os cuidados de saúde e promovendo a funcionalidade, a participação social e a alfabetização física das crianças e adolescentes com TEA, em diferentes contextos.

CRONOGRAMA

**Etapa**	**Início- Término**

Escrita do projeto e aprovação câmara departamental	Janeiro 2022- Fevereiro 2022
Submissão ao comitê de ética e pesquisa	Março 2022-Agosto 2022
Treinamento da equipe e recrutamento dos participantes	Agosto 2022- Dezembro 2022
Avaliações iniciais e início da Intervenção (fase 1)	Janeiro 2023- Março 2023
Reavaliações (fase 1) e início fase 2 e 3	Março 2023- Abril 2023
Reavaliações e follow-up (fase 1)	Junho 2023- Julho 2023
Análise dos dados	Julho 2023- Novembro 2023
Escrita do artigo científico	Dezembro 2023- Abril 2024

MEDIDAS DE SEGURANÇA (COVID-19)

Devido ao atual período de pandemia da COVID-19, ressaltamos que o início do projeto só iniciará, após autorização prévia do Programa de Pós-Graduação em Ciências da Reabilitação e Direção da EEFFTO-UFMG, em consonância com as fases de flexibilização propostas pela Universidade Federal de Minas Gerais. Possíveis alterações no cronograma poderão ocorrer e serão atualizadas via Plataforma Brasil. Serão seguidas todas as normas de segurança estabelecidas pelos órgãos competentes.

LOCAL DE COLETA

As coletas e intervenções serão realizadas nas quadras abertas da Escola de Educação Física, Fisioterapia e Terapia Ocupacional (EEFFTO) (anexo 6) e MultiLAB da Universidade Federal de Minas Gerais – UFMG, conforme disponibilidade e agendamento.

ORÇAMENTO

Segue abaixo os principais materiais que serão utilizados para essa pesquisa e o orçamento previsto.

**Item**	**Quantidade**	**Material**	**Valor unitário**	**Valor total**
1	2 pct	Folha A4	24,00	48,00
2	1000	Impressões	0,50	500,00
3	4	Bolas de basquete	50,00	200,00
4	4	Bolas de futebol	40,00	160,00
5	4	Bolas de futsal	45,00	180,00
6	4	Bastão de revezamento	90,00	360,00

	**1.448,00**

Os custos do projeto como materiais esportivos e recreativos serão arcados pelos próprios pesquisadores.

Fase 2: Avaliação das experiências dos participantes da intervenção *Sports Stars* e suas famílias

Objetivos do estudo

Os objetivos desta fase do projeto são: (1) explorar as experiências dos participantes e de suas famílias que participaram do programa Sports Stars e identificar os resultados percebidos por eles acerca dos domínios da alfabetização física, (2) comparar a percepção dos pacientes e dos pais/cuidadores e (3) extrair as experiências específicas sobre os facilitadores e barreiras que ajudaram e que impediram a participação em atividades de lazer para crianças e adolescentes TEA, a fim de informar a prática e pesquisas futuras.

Desenho do estudo

Estudo Qualitativo

Participantes

Serão recrutados por conveniência crianças e adolescentes com idades de 6 a 18 anos e seu cuidador principal, participantes da intervenção Sports Stars Brasil (grupo intervenção da fase 1) com TEA leve a moderado na CARS e nível I e II ACSF: SC. Serão excluídos os participantes que se desligarem do programa *Sports Stars* antes da sua finalização (8 encontros). Os participantes serão recrutados até que a saturação que refere-se ao ponto durante a coleta de dados em que as ligações entre os dados qualitativos dos grupos focais não têm mais do que 5% de novas categorias da alfabetização física e da CIF comparada aos demais grupos focais.

Procedimentos e coleta de dados:

Após participarem da intervenção *Sports Stars* Brasil, será realizado grupos focais para a coleta de dados com as crianças, com os adolescentes e com um dos familiares de cada participante. Para cada grupo focal será realizado a coleta dos dados apenas uma vez e presencialmente, tendo expectativa de duração para cada entrevista 1 hora, podendo ser

estendida se for necessário. Para que ocorra homogeneidade na coleta dos dados, os entrevistadores responsáveis passarão por um treinamento.

Antes de cada grupo focal os participantes serão esclarecidos sobre os objetivos do estudo e se concordarem participar, irão assinar os termos de consentimento e assentimento livre e esclarecido. Os dados de caracterização da amostra, considerados importantes para o presente estudo, que serão coletados antes das entrevistas: idade, sexo, classificação e nível do autismo (no caso das crianças e adolescentes), nível socioeconômico e escolaridade. A coleta de dados será realizada na Escola de Educação Física, Fisioterapia e Terapia Ocupacional da Universidade Federal de Minas Gerais. No momento da entrevista, apenas os participantes e entrevistadores estarão presentes.

Grupos Focais:

Serão realizadas perguntas semi-estruturadas com conteúdos relacionados a alfabetização física, no contexto da percepção do desempenho dos domínios da competência física, social, psicológica e cognitiva e referente as barreiras e facilitadores na participação das atividades de lazer dentro do modelo da CIF que será utilizada como referência para a análise dos dados por meio da vinculação dos dados qualitativos de acordo com as regras propostas por Cieza. A et al. 2002. Os grupos focais serão realizados imediatamente após a intervenção, a entrevista será gravada e posteriormente transcrita.

Após esse processo, o entrevistado terá acesso a transcrição para confirmação dos dados e informações presentes antes da análise do conteúdo pelos pesquisadores. Além disso, durante as entrevistas e condução dos grupos focais, caso o avaliador julgue necessário, serão feitas anotações. Com a anuência dos participantes, será realizado as análises dos dados.

Análise de dados:

Os depoimentos de todos os participantes serão transcritos de forma literal. Após esse momento, os textos serão coletados para análise qualitativa. Os dados serão analisados através de análise de conteúdo. As respostas serão codificadas e categorizadas, utilizando o *software* NVivo. A codificação do será realizada por dois investigadores para garantir a concordância durante a codificação, um terceiro investigador participará deste processo em caso de discordância. As principais citações acerca dos temas sobre alfabetização

física serão identificadas e os códigos serão então mapeados para a *categoria* que corresponder mais precisamente a cada código, utilizando os critérios descritos por (EDWARDS et al., 2017). (tabela 1).

Referente a participação em atividades de lazer, barreiras e facilitadores, será utilizada questões abertas, adaptadas de (Longo, E, *et al*; 2020): “De quais atividades de lazer você participou nos último 2 meses / 8 semanas, além das que você fez na EEFFTO?”; “Com quem e onde você fez? Você gostou de fazer as atividades com eles?"; “Que outras atividades de lazer você gostaria de fazer e não fez?”; “O que é um obstáculo para você realizar as atividades de lazer? E quais são os facilitadores para que você realize as atividades de lazer?”; e “Se fosse possível, o que você mudaria para melhorar sua participação nas atividades de lazer ?” O moderador também fez anotações seguindo cada grupo. As citações serão identificadas e codificadas dentre os cinco componentes de funcionalidade da CIF: (1) Funções do corpo; (2) Estruturas corporais; (3) Atividades e participação; (4) Fatores ambientais; e (5) Fatores pessoais, os conceitos que não estirem dentro dos componentes da CIF serão atribuído a categoria de “não coberto”.

Os componentes caracterizados na (tabela 2), consistem em capítulos com categorias hierárquicas da CIF como unidades de classificação. Onde cada categoria da CIF é atribuído um código alfanumérico e cada classificação é representada por um letra e seguida por uma especificação de segundo nível esta é uma letra que representa o componente de classificação.

Um sumário preliminar da codificação será compartilhado com os participantes para avaliar a precisão das informações coletadas.

**Tabela 1. Critérios para codificação da Alfabetização Física**	
**1. Física**	Citações relacionadas com a capacidade física, habilidades motoras e movimentos fundamentais.
**2. Social**	Citações relacionadas as relações sociais da criança/adolescente.
**3. Psicológica**	Citações relacionadas a motivação, engajamento, comprometimento e participação.
**4. Cognitiva**	Citações relacionadas ao conhecimento, raciocínio, discernimento, entendimento da criança/adolescente

**Tabela 2. Critérios para codificação da CIF**

**1. B**: Funções do Corpo	Citações referente as funções fisiológicas dos sistemas do corpo (inclusive funções psicológicas)
**2. S:** Estruturas do Corpo	Citações referente as partes anatômicas do corpo como órgãos, membros e seus componentes.
**3. D:** Atividades e Participação	Citações referente a execução de uma tarefa ou ação por uma criança ou adolescente com TEA e ao envolvimento em situações de vida diária.
**4. E:** Fatores Ambientais	Citações referente ao ambiente físico, social e de atitude no qual uma criança ou adolescente com TEA vive e conduz sua vida.
**5. Facilitadores e Barreiras**	Citações referente facilitadores e barreiras que uma criança ou adolescente com TEA pode encontrar na execução de atividades ou problemas que um indivíduo pode ter ao se envolver em situações de vida

Fase 3: propriedades de medidas dos instrumentos Ignite Challenge, 10x5 Sprint Test, Muscle Power Sprint Test e Teste do Desenvolvimento Motor Grosso – segunda edição – TGMD-2

Objetivo: Verificar a confiabilidade dos instrumentos Ignite Challenge, 10x5 Sprint Test, Muscle Power Sprint Test e Teste do Desenvolvimento Motor Grosso – segunda edição – TGMD-2, através da análise da consistência interna e da reprodutividade (teste-reteste), com crianças e adolescentes TEA.

Desenho do estudo

Estudo observacional transversal

Participantes

Serão recrutados por conveniência crianças e adolescentes com idades de 6 a 18 anos e seu cuidador principal, participantes da intervenção Sports Stars Brasil (grupo intervenção da fase 1) com TEA classificados por nível de suporte nível I e II através da ACSF: SC.

Cálculo amostral

O tamanho da amostra seguirá as recomendações do Consensus-based Standards for the selection of health Measurement Instruments (COSMIN) (Terwee et al., 2012). Segundo Terwee et *al.* (2011) uma amostra de 50 indivíduos é adequada para análise da confiabilidade teste-reteste e do erro padrão de medida.

Instrumentos

O *Ignite Challenge* é uma medida baseada em capacidade de 13 itens que avalia a precisão e velocidade das habilidades locomotoras e de controle de objetos para crianças com TEA a partir de 6 anos de idade, classificadas nos níveis ACSF I e II. O *Ignite Challenge* foi desenvolvido com base no teste Challenge de 25 itens para crianças com PC (ARBOR-NICITOPOULOS ET AL., 2018; 2021). O *Ignite Challenge* usa "cartões com figuras" para cada item do teste para complementar as demonstrações dos avaliadores e melhorar a compreensão de cada item do teste (ALLEN ET AL., 2017; LIU & BRESLIN, 2013) (BRESLIN & RUDISILL, 2011). O *Ignite Challenge* demonstrou excelente interexaminador (ICC=0,91 (95%= 0,93, 0,99), intraexaminador (ICC=0,96 (IC 95%= 0,90, 0,98)) e teste de repouso (ICC=0,91 (95% CI=0,84, 0,95) confiabilidade em crianças australianas (EVANS et al 2021) A confiabilidade para a população brasileira de TEA será avaliada por nossa equipe de pesquisa como parte deste projeto.

O Muscle Power Sprint Test (MPST) é um teste de campo simples para avaliar o desempenho anaeróbico em crianças e adolescentes. O participante é solicitado a correr o mais rápido possível por 15 m (marcados por linhas e cones), 6 vezes, com intervalo de 10 segundos entre cada sprint. Leva alguns minutos para ser concluído e requer apenas um espaço aberto, um cronômetro e dois cones. O MPST tem alta confiabilidade interobservador e teste-reteste (r = 0,97–0,99) para crianças com PC que andam (VERSCHUREN O, TAKKEN T, KETELAAR M, GORTER J.W, HOLDERS P.J.M, 2007). As propriedades em crianças e adolescentes com TEA serão estudadas neste estudo.

O 10×5 Sprint Test (10×5ST) avalia a agilidade e a capacidade anaeróbica em crianças ambulantes com PC. Neste teste, os participantes precisam correr 5 m separados por 2 cones, 10 vezes continuamente, dando voltas nos cones que marcam o final dos cinco metros (VERSCHUREN, TAKKEN, KETELAAR, GORTER, HELDERS, 2007). O Teste Sprint 10 × 5 Metros apresenta excelente confiabilidade interobservador (CCI > 0,97) e teste-reteste (r = 1), além de boa validade de construto relatada. Um aumento no tempo de exercício de 3,2 segundos é considerado uma mudança real (VERSCHUREN, TAKKEN, KETELAAR, GORTER, HELDERS, 2007). No entanto, as propriedades psicométricas ainda não foram estudadas na população de TEA, por isso serão investigadas neste estudo.

O TGMD-2 (anexo 4) é um teste para avaliação do desenvolvimento motor grosso de meninos e meninas entre três e 10 anos. O teste avalia 12 habilidades motoras fundamentais, das quais seis são habilidades de locomoção (correr, galopar, saltitar, dar uma passada, saltar horizontalmente e correr lateralmente) e seis são habilidades de controle de objetos (rebater, quicar, receber, chutar, arremessar por cima do ombro e rolar uma bola). Para cada habilidade são observados de 3 a 5 critérios motores específicos. O TGMD-2 permite uma avaliação separada de cada subteste (locomoção e controle de objeto) e ainda no subteste de controle de objeto, uma diferenciação por gênero. Os escores totais de cada subteste são somados e representados como escores brutos, que podem ser convertidos em quocientes motores (ULRICH, 2000). O TGMD-2 foi validado e confiável para crianças brasileiras no estudo de Valentini et al., (2012) e tem sido utilizado em crianças com TEA (KRUGER, SILVEIRA; MARQUES, 2019; MOHD NORDIN; ISMAIL & KAMAL NOR, 2021). O TGMD-2 possui excelentes índices de confiabilidade e validade (CAPIO; SIT; ABERNETHY, 2011a, 2011b). Entretanto, não apresenta valores de confiabilidade para população TEA.

Procedimentos

Confiabilidade

A confiabilidade teste-reteste dos instrumentos será realizada em dois momentos distintos, num intervalo de sete a 10 dias, por um mesmo examinador que irá avaliar as 50 crianças e adolescentes. Estas avaliações serão filmadas com uma câmera de vídeo e pontuadas posteriormente pelo examinador. Um segundo examinador assistirá e pontuará a primeira avaliação de cada participante, para a análise da confiabilidade inter-examinador. Ambos examinadores possuem certificação para administração do *Ignite Challenge*. Para a aplicação dos demais instrumentos nenhuma certificação é necessária, apenas treinamento prévio.

Responsividade e Índices de Mudança

Após a primeira avaliação da confiabilidade teste-reteste, os participantes que tiverem realizado intervenção (tratamento fisioterápico semanal, individual ou em grupo, durante 30 a 45 minutos) com duração de três meses, os instrumentos serão administrados novamente e filmado em câmera de vídeo. Um avaliador assistirá os vídeos da primeira

avaliação da análise da confiabilidade teste-reteste e da reavaliação e ponturá essas avaliações, será cegado quanto a data. A abordagem baseada na âncora será utilizada para análise do o índice de mudança mínima clinicamente importante (MMCI). Nessa abordagem, o índice é calculado baseando-se em um critério externo (Copay et al., 2007). Ao realizar esta abordagem deve-se assegurar que a âncora utilizada seja capaz de identificar os participantes que obtiveram uma mudança clinicamente importante (Engel et al., 2018). Será utilizado como critério externo uma Escala de Pontuação Global de três pontos (piorou, sem mudanças, melhorou) (Guyatt et al., 2002). Após dois meses de tratamento, os pais/responsáveis dos participantes serão perguntados como eles avaliavam as mudanças na função motora grossa de seus filhos(as). Participantes classificados como "melhorou" na percepção dos pais serão considerados como apresentando mudança clinicamente importante nos instrumentos.

Análise estatística

O Coeficiente de Correlação intra-classe (CCI) tipo será utilizado para avaliar a confiabilidade da pontuação total para cada um dos instrumentos. Valores abaixo de 0.74 representam confiabilidade moderada a pobre, entre 0.75 e 0.89 informam sobre boa confiabilidade e valores acima de 0.90, excelente (Portney & Walkins, 2009). O índice Kappa Ponderado (k) com o método de pesos incrementais será utilizado para avaliar a confiabilidade de cada item do teste (Portney & Walkins, 2009). Valores abaixo de 0.20 representam confiabilidade pobre, valores entre 0.20 e 0.40 sugerem confiabilidade razoável, entre 0.41 e 0.60 moderada, valores entre 0.61 e 0.80 substancial e acima de 0.80 quase perfeita (Landis & Koch et al., 2012). Intervalos de confiança a 95% (IC 95%) acompanharam os índices. Além disso, serão utilizados erro padrão de medida combinado (EPM) e coeficiente de variação (CV) como índices de variação. Valores de CV serão considerados adequados quando abaixo de 10% (Bruton et al., 2000). Com o objetivo de complementar os resultados dos índices de confiabilidade, o teste-t independente será realizado na análise da confiabilidade interexaminador e o teste-t pareado, na confiabilidade teste-reteste. Além disso, a análise de BlandAltman com seus limites de concordância a 95% será utilizada para ilustrar graficamente a variação dos escores nos instrumentos de cada participante com relação à média das duas ocasiões (teste-reteste) ou dos dois avaliadores (inter-examinador) (Bruton et al., 2000). A responsividade do instrumento será analisada pelo teste-t pareado. Medidas de tamanho de efeito (d) de

magnitude entre 0.20 e 0.50 revelaram efeito pequeno, entre 0.50 e 0.80 efeito moderado e acima de 0.80 efeito grande (COHEN, 1988). O MMD será estimado com dois intervalos de confiança: 90% (MMD90) para comparação com o valor reportado na versão original do instrumento, e 95% (MMD95) para auxiliar na interpretação dos valores de mudança (Terwee et al., 2007), através das fórmulas abaixo: $MMD90 = 1.65 \times \sqrt{2} \times EPM$ e $MMD95 = 1.96 \times \sqrt{2} \times EPM$ Onde EPM = erro padrão de medida.

Anteriormente ao cálculo do MMCI, o coeficiente de correlação de postos de Spearman (rs) vai estimar a correlação entre as mudanças nos escores dos instrumentos, após dois meses de tratamento, e a percepção de mudança dos pais/responsáveis pela Escala de Pontuação Global; onde r maior ou igual a 0.30 indicam que a âncora é considerada adequada para estimar o MMCI (Revicki et al., 2008). Em seguida, o MMCI será estimado pelo método da média de mudança (Engel et al., 2018), onde o índice é considerado o valor da média da pontuação dos testes, entre os participantes classificados como os que melhoraram segundo a Escala de Pontuação Global. Todas as análises foram conduzidas no Statistical Package for the Social Sciences (SPSS), versão 19.0.

REFERÊNCIAS

American Psychiatric Association. **Manual de diagnóstico e estatístico de transtornos mentais:** DSM-5. 5. ed. Porto Alegre: Artmed; 2014. 848 p.

ANDRADE, M.M.A. Análise da influência da abordagem de integração sensorial de Ayres® na participação escolar de alunos com transtorno do espectro autista. 2020.

BARROS, S.S.H. Padrão de prática de atividades físicas de crianças em idade pré- escolar. [Dissertação]. 2005. Pós-graduação em Educação Física da Universidade Federal de Santa Catarina.

BEATON, D.E. et al. Diretrizes para o processo de adaptação transcultural de medidas de autorrelato. **Spine** , v. 25, n. 24, pág. 3186-3191, 2000.

BEDELL, G.M. et al. Parent perspectives to inform development of measures of children's participation and environment. **Archives of Physical Medicine and Rehabilitation**, v. 92, n. 5, p. 765-773, 2011.

BURDETTE, H.L. et al. Parental report o f outdoor playtime as a measure of physical activity in preschool children. **Archives of Pediatrics & Adolescent Medicine**, 2004;158(4):353- 357.

CAMARGO, S. P. H.; RISPOLI, M. Análise do comportamento aplicada como intervenção para o autismo: definição, características e pressupostos filosóficos. **Revista Educação Especial**, v. 26, n. 47, p. 639-650, set./dez, 2013.

CAMARGO, S. P. H.; RISPOLI, M. Análise do comportamento aplicada como intervenção para o autismo: definição, características e pressupostos filosóficos. **Revista Educação Especial**, v. 26, n. 47, p. 639-650, set./dez, 2013 *apud* SKINNER, B. F. **Science and human behavior**. New York: Free Press, 1953.

CAPIO, C. M.; SIT, C. H. P.; ABERNETHY, B. Fundamental movement skills testing in children with cerebral palsy. **Disability and Rehabilitation**, v. 33, n. 25–26, p. 2519–2528, 2011b.

CARLON, S. L. et al. Differences in habitual physical activity levels of young people with cerebral palsy and their typically developing peers: A systematic review. **Disability and Rehabilitation**, v. 35, n. 8, p. 647–655, 2013.

CHAN, A.; TETZLAFF, J. M.; ALTMAN, D. G. SPIRIT 2013 Statement : Defining Standard Protocol Items for Clinical Trials. **Ann Intern Med**, v. 158, n. 3, p. 200–207, 2016.

CIEZA, A. et al. Linking health-status measurements to the international classification of functioning, disability and health. **Journal of rehabilitation medicine**, v. 34, n. 5, p. 205-210, 2002.

CLUTTERBUCK, G.; AULD, M.; JOHNSTON, L. Active exercise interventions improve gross motor function of ambulant/semi-ambulant children with cerebral palsy: a systematic review. **Disability and Rehabilitation**, v. 41, n. 10, p. 1131–1151, 2019a.

CLUTTERBUCK, G. L.; AULD, M. L.; JOHNSTON, L. M. SPORTS STARS study protocol: A randomised, controlled trial of the effectiveness of a physiotherapist-led modified sport intervention for ambulant school-aged children with cerebral palsy. **BMC Pediatrics**, v. 18, n. 1, p. 1–10, 2018.

CLUTTERBUCK, G. L.; AULD, M. L.; JOHNSTON, L. M. Performance of school-aged children with cerebral palsy at GMFCS levels I and II on high-level, sports-focussed gross motor assessments. **Disability and Rehabilitation**, v. 0, n. 0, p. 1–9, 2019b.

CLUTTERBUCK, Georgina L.; AULD, Megan L.; JOHNSTON, Leanne M. SPORTS STARS: a practitioner-led, peer-group sports intervention for ambulant children with cerebral palsy. Activity and participation outcomes of a randomised controlled trial. **Disability and Rehabilitation**, p. 1-9, 2020a.

CLUTTERBUCK, Georgina L.; AULD, Megan L.; JOHNSTON, Leanne M. SPORTS STARS: a practitioner-led, peer-group sports intervention for ambulant, school-aged children with cerebral palsy. Parent and physiotherapist perspectives. **Disability and Rehabilitation**, p. 1-10, 2020b.

COLOMBO-DOUGOVITO, A. M.; BLOCK, M. E.; ZHANG, X.; STREHLI, I. A multiple-method review of accommodations to gross motor assessments commonly used with children and adolescents on the autism spectrum. Autism, 24, n. 3, p. 693-706, 2020.

COSTER, W. et al. Psychometric evaluation of the Participation and Environment Measure for Children and Youth. **Developmental Medicine and Child Neurology**, v. 53, n. 11, p. 1030–1037, 2011.

COSTER, W. et al. Development of the participation and environment measure for children and youth: Conceptual basis. **Disability and Rehabilitation**, v. 34, n. 3, p. 238–246, 2012.

CRAIG, F. et al. Motor Competency and Social Communication Skills in Preschool Children with Autism Spectrum Disorder. **Autism Research**, v. 11, p. 893–902, 2018.

DI REZZE, Briano et al. Developing a classification system of social communication functioning of preschool children with autism spectrum disorder. **Developmental Medicine & Child Neurology**, v. 58, n. 9, p. 942-948, 2016.

DOYLE, C.A. & MCDOUGLE, C.J. Pharmacologic treatments for the behavioral symptoms associated with autism spectrum disorders across the lifespan. **Dialogues in clinical neuroscience**, v. 14, n. 3, p. 263, 2012.

DUNN, W. Caregiver Questionnaire–Sensory Profile. **United States of America**, 1999.

DUNN, W. **Sensory profile 2:** user's manual. San Antonio: NCS Pearson, 2014.

EDWARDS, L. C. et al. Definitions, Foundations and Associations of Physical Literacy: A Systematic Review. **Sports Medicine**, v. 47, n. 1, p. 113–126, 2017.

ELOI, D. S. et al. Adaptação transcultural do instrumento Autism Classification System of Functioning: Social Communication (ACSF: SC) para uso no Brasil. **Cad. Bras. Ter. Ocup.**, São Carlos, v. 27, n. 2, p. 293-301, 2019.

GALVÃO, É. R. V. P. et al. Medida da Participação e do Ambiente - Crianças e Jovens (PEM-CY). **Revista de Terapia Ocupacional da Universidade de São Paulo**, v. 29, n. 3, p. 237–245, 2018.

HALEY, S.M. et al. PEDI-CAT Version 1.4. 0: development, standardisation and administration manual. **Boston: Trustees of Boston Universtiy**, 2011.

HALEY, S.M. et al. Accuracy and precision of the Pediatric Evaluation of Disability Inventory computer-adaptive tests (PEDI-CAT). **Developmental Medicine & Child Neurology**, v. 53, n. 12, p. 1100-1106, 2011.

HILLIER, A; BUCKINGHAM, A; SCHENA, D. Physical activity among adults with autism: participation, attitudes, and barriers. **Perceptual and Motor Skills**, v. 127, n. 5, p. 874-890, 2020.

HOWELLS, K et al. Efficacy of group-based organised physical activity participation for social outcomes in children with autism spectrum disorder: a systematic review and meta-analysis. **Journal of autism and developmental disorders**, v. 49, n. 8, p. 3290-3308, 2019.

HUANG, J et al. "Meta-Analysis on Intervention Effects of Physical Activities on Children and Adolescents with Autism." **International journal of environmental research and public health** vol. 17,6 1950. 17 Mar. 2020

KHOURY, L. P. et al. **Manejo comportamental de crianças com Transtornos do Espectro do Autismo em condição de inclusão escolar:** Guia de orientações a professores [livro eletrônico]. São Paulo: Editora MEMNON, 2014.

KRAMER, J.M et al. “A new approach to the measurement of adaptive behavior: development of the PEDI-CAT for children and youth with autism spectrum disorders.” *Physical & occupational therapy in pediatrics* vol. 32,1 (2012).

KRIEGER, B et al. Cross-cultural adaptation of the Participation and Environment Measure for Children and Youth (PEM-CY) into German: a qualitative study in three countries. **BMC pediatrics**, v. 20, n. 1, p. 1-15, 2020.

KRUGER, G. R.; SILVEIRA, J. R.; MARQUES, A. C. Motor skills of children with autism spectrum disorder. **Revista Brasileira de Cineantropometria & Desempenho Humano**, 21, 2019.LAW, M. et al. Medida Canadense de Desempenho Ocupacional (COPM). Organização e Tradução Lívia de Castro Magalhães, Lílian Vieira Magalhães, Ana Amélia Cardoso. Belo Horizonte: **Editora UFMG**, 2009.

Lewis M, Bromley K, Sutton CJ, McCray G, Myers HL, Lancaster GA. Determining sample size for progression criteria for pragmatic pilot RCTs: the hypothesis test strikes back!. Pilot Feasibility Stud. 2021;7(1):40. Published 2021 Feb 3. doi:10.1186/s40814-021-00770-x.

LI, YONG-JIANG et al. Global prevalence of obesity, overweight and underweight in children, adolescents and adults with autism spectrum disorder, attention-deficit hyperactivity disorder: A systematic review and meta-analysis. **Obesity Reviews**, v. 21, n. 12, p. e13123, 2020.

LIU, T. Sensory Processing and Motor Skill Performance in Elementary School Children with Autism Spectrum Disorder. **Perceptual & Motor Skills: Physical Development & Measurement**, v. 116, n. 1, p. 197-209, 2013.

MAENNER, M. J. et al. Prevalence of Autism Spectrum Disorder Among Children Aged 8 Years — Autism and Developmental Disabilities Monitoring Network, 11 Sites, United States, 2016. **Morbidity and Mortality Weekly Report**, v. 69, n. 4, March, 2020.

MANCINI, M. C. et al. New version of the Pediatric Evaluation of Disability Inventory (PEDI-CAT): translation, cultural adaptation to Brazil and analyses of psychometric properties. **Brazilian journal of physical therapy**, v. 20, n. 6, p. 561-570, 2016.

MASSION, J. Sport et autism. **Science & Sports**, v. 21, p. 243-248, 2006.

MACDONALD, M; ESPOSITO, P; ULRICH, D. The physical activity patterns of children with autism. **BMC research notes**, v. 4, n. 1, p. 1-5, 2011.

MATTOS, J.C; D'ANTINO, M.E.F; CYSNEIROS, R.M. Tradução para o português do Brasil e adaptação cultural do Sensory Profile. **Psicologia: teoria e prática**, v. 17, n. 3, p. 104-120, 2015.

MOHD NORDIN, A; ISMAIL, J & KAMAL NOR, N. Motor development in children with autism spectrum disorder. **Frontiers in pediatrics**, p. 889, 2021.

OBRUSNIKOVA, I.; CAVALIER, A. R. Perceived Barriers and Facilitators of Participation in After-School Physical Activity by Children with Autism Spectrum Disorders. **Journal of Developmental Physical Disabilities**, v. 23, p.195–211, 2011.

OHARA, Reiko et al. Association between social skills and motor skills in individuals with autism spectrum disorder: a systematic review. **European Journal of Investigation in Health, Psychology and Education**, v. 10, n. 1, p. 276-296, 2020.

PAN, C. Y.; FREY, G. C. Identifying Physical Activity Determinants in Youth with Autistic Spectrum Disorders. **Journal of Physical Activity and Health**, v. 2, p. 412-422, 2005.

PATEL, D. R.; SOARES, N.; WELLS, K. Neurodevelopmental readiness of children for participation in sports. **Translational Pediatrics**, v. 6, n. 3, p. 167–173, 2017.

PEREIRA, A.; RIESGO, R. S.; WAGNER, M. B. Childhood autism: translation and validation of the Childhood Autism Rating Scale for use in Brazil. **Jornal de Pediatria,** v. 84, n. 6, p. 487-494, 2008.

Portney, L., & Watkins, M. (2000). Power and sample size. Foundations of Clinical Research. New Jersey: Prentice Hall Health, 705-30.

PORTNEY, Leslie Gross et al. Foundations of clinical research: applications to practice. **Upper Saddle River**, NJ: Pearson/Prentice Hall, 2009.

RAPIN, I.; GOLDMAN, S. The Brazilian CARS: a standardized screening tool for autism. **Jornal de Pediatria**, v. 84, n. 6, 2008.

ROBERTSON, C E.; BARON-COHEN, S. Sensory perception in autism. **Nature Reviews Neuroscience**, v. 18, n. 11, p. 671-684, 2017.

ROSENBAUM, P.; GORTER, J. W. The “F-words” in childhood disability: I swear this is how we should think. **Child: Care, Health and Development**, v. 38, n. 4, p. 457–463, 2012.

SIMPSON, K et al. Investigating the participation of children on the autism spectrum across home, school, and community: A longitudinal study. **Child: care, health and development**, v. 45, n. 5, p. 681-687, 2019.

SOCIEDADE BRASILEIRA DE PEDIATRIA. Manual de Orientação Departamento Científico de Pediatria do Desenvolvimento e Comportamento Transtorno do Espectro do Autismo, n. 5, Abr, 2019.

SOWA, M; MEULENBROEK, R. Effects of physical exercise on autism spectrum disorders: a meta-analysis. **Research in autism spectrum disorders**, v. 6, n. 1, p. 46-57, 2012.

SRINIVASAN, S M.; PESCATELLO, L S.; BHAT, A N. Current perspectives on physical activity and exercise recommendations for children and adolescents with autism spectrum disorders. **Physical therapy**, v. 94, n. 6, p. 875-889, 2014.

STANISH, H. I. et al. Physical activity levels, frequency, and type among adolescents with and without autism spectrum disorder. **Journal of autism and developmental disorders**, v. 47, n. 3, p. 785-794, 2017.

STINS, J F.; EMCK, C. Balance performance in autism: A brief overview. **Frontiers in psychology**, v. 9, p. 901, 2018.

TERWEE C, BOT S, BOER M, WINDT D, KNOL D, DEKKER J, BOUTER L, VET H. Quality Criteria were Proposed for Measurement Properties of Health Status Questionnaires. Journal of Clinical Epidemiology. 2007; 60: 34-42.

TERWEE, C. B. et al. Rating the methodological quality in systematic reviews of studies on measurement properties: a scoring system for the COSMIN checklist. **Quality of life research**, v. 21, n. 4, p. 651-657, 2012.

TERWEE C.B, MOKKINK L.B, KNOL D.L, OSTELO R.W.J.G, BOUTER L.M, DE VET H.C.W. Rating the methodological quality in systematic reviews of studies on measurement properties: a scoring system for the COSMIN checklist. **Quality of Life Research** 2011, July 6

ULRICH, D. A. Test of Gross Motor Development, 2nd ed. n. June, 2000.

VARNI, J. W.; BURWINKLE, T. M.; SEID, M. The PedsQL TM 4.0 as a school population health measure: Feasibility, reliability, and validity. **Quality of Life Research**, v. 15, n. 2, p. 203–215, 2006.

VERSCHUREN, O. et al. Reliability for Running Tests for Measuring Agility and Anaerobic Muscle Power in Children and Adolescents with Cerebal Palsy. [s.d.].

VERSCHUREN, O. et al. Validity of the muscle power sprint test in ambulatory youth with cerebral palsy. **Pediatric Physical Therapy,** v. 25, n. 1, p. 25–28, 2013.7

VERSCHUREN, Olaf et al. Exercise and physical activity recommendations for people with cerebral palsy. **Developmental Medicine & Child Neurology**, v. 58, n. 8, p. 798-808, 2016.

WILLIAMS, K et al. Functioning, participation, and quality of life in children with intellectual disability: an observational study. **Developmental Medicine & Child Neurology**, v. 63, n. 1, p. 89-96, 2021.

World Health Organization, The ICD-10 Classification of Mental and Behavioral Disorders: Clinical Descriptions and Diagnostic Guidelines. Geneva, Switzerland World Health Organization 1992;

ANEXO 1 – CARS - CHILDHOOD AUTISM RATING SCALE

CARS-Childhood Autism Rating Scale VERSÃO EM PORTUGUÊS

	I. RELAÇÕES PESSOAIS
**1**	Nenhuma evidência de dificuldade ou anormalidade nas relações pessoais: O comportamento<br>da criança é adequado à sua idade. Alguma timidez, nervosismo ou aborrecimento podem serobservados quando é dito à criança o que fazer, mas não em grau atípico.
1.5	
**2**	Relações levemente anormais: A criança pode evitar olhar o adulto nos olhos, evitar o adulto ou ter uma reação exagerada se a interação é forçada, ser excessivamente tímido, não responderao adulto como esperado ou agarrar-se ao pais um pouco mais que a maioria das crianças da mesma idade
2.5	
**3**	Relações moderadamente anormais: Às vezes, a criança demonstra indiferença (parece ignorar o adulto). Outras vezes, tentativas persistentes e vigorosas são necessárias para se conseguir a atenção da criança. O contato iniciado pela criança é mínimo.

3.5<br>**4**	Relações gravemente anormais: A criança está constantemente indiferente ou inconsciente ao que o adulto está fazendo. Ela quase nunca responde ou inicia contato com o adulto. Somente a tentativa mais persistente para atrair a atenção tem algum efeito.
	**Observações:**

	II. IMITAÇÃO
**1**	Imitação adequada: A criança pode imitar sons, palavras e movimentos, os quais são adequados para o seu nível de habilidade.
1.5<br>**2**	Imitação levemente anormal: Na maior parte do tempo, a criança imita comportamentos simples como bater palmas ou sons verbais isolados; ocasionalmente imita somente após estimulação ou com atraso.
2.5<br>**3**	Imitação moderadamente anormal: A criança imita apenas parte do tempo e requer uma grande dose de persistência ou ajuda do adulto; freqüentemente imita apenas após um tempo (com atraso).
3.5<br>**4**	Imitação gravemente anormal: A criança raramente ou nunca imita sons, palavras ou movimentos mesmo com estímulo e assistência.
	**Observações:**

III. RESPOSTA EMOCIONAL

**1**	Resposta emocional adequada à situação e à idade: A criança demonstra tipo e grau adequados de resposta emocional, indicada por uma mudança na expressão facial, postura e conduta.
1.5	
**2**	Resposta emocional levemente anormal: A criança ocasionalmente apresenta um tipo ou grau inadequados de resposta emocional. As vezes, suas reações não estão relacionadas a objetos ou a eventos ao seu redor.
2.5	
**3**	Resposta emocional moderadamente anormal: A criança demonstra sinais claros de resposta emocional inadequada (tipo ou grau). As reações podem ser bastante inibidas ou excessivas e sem relação com a situação; pode fazer caretas, rir ou tornar-se rígida até mesmo quando não estejam presentes objetos ou eventos produtores de emoção.
3.5	
**4**	Resposta emocional gravemente anormal: As respostas são raramente adequadas a situação. Uma vez que a criança atinja um determinado humor, é muito difícil alterá-lo. Por outro lado, a criança pode demonstrar emoções diferentes quando nada mudou.
	**Observações:**

IV. USO CORPORAL

**1**	Uso corporal adequado à idade: A criança move-se com a mesma facilidade, agilidade e coordenação de uma criança normal da mesma idade.
1.5	
**2**	Uso corporal levemente anormal: Algumas peculiaridades podem estar presentes, tais como falta de jeito, movimentos repetitivos, pouca coordenação ou a presença rara de movimentos incomuns
2.5	
**3**	Uso corporal moderadamente anormal: Comportamentos que são claramente estranhos ou incomuns para uma criança desta idade podem incluir movimentos estranhos com os dedos, postura peculiar dos dedos ou corpo, olhar fixo, beliscar o corpo, auto-agressão, balanceio, girar ou caminhar nas pontas dos pés.
3.5	
**4**	Uso corporal gravemente anormal: Movimentos intensos ou freqüentes do tipo listado acimasão sinais de uso corporal gravemente anormal. Estes comportamentos podem persistir apesar das tentativas de desencorajar as crianças a fazê-los ou de envolver a criança em outras atividades.
	**Observações:**

V. USO DE OBJETOS

**1**	Uso e interesse adequados por brinquedos e outros objetos: A criança demonstra interesse normal por brinquedos e outros objetos adequados para o seu nível de habilidade e os utiliza de maneira adequada.
1.5	
**2**	Uso e interesse levemente inadequados por brinquedos e outros objetos: A criança pode demonstrar um interesse atípico por um brinquedo ou brincar com ele de forma inadequada, de um modo pueril (exemplo: batendo ou sugando o brinquedo)
2.5	
**3**	Uso e interesse moderadamente inadequados por brinquedos e outros objetos: A criança pode demonstrar pouco interesse por brinquedos ou outros objetos, ou pode estar preocupada emusá-los de maneira estranha. Ela pode concentrar-se em alguma parte insignificante do

brinquedo, tornar-se fascinada com a luz que reflete do mesmo, repetitivamente mover alguma parte do objeto ou exclusivamente brincar com ele.

3.5

4 Uso e interesse gravemente inadequados por brinquedos e outros objetos: A criança pode engajar-se nos mesmos comportamentos citados acima, porém com maior freqüência e intensidade. É difícil distrair a criança quando ela está engajada nestas atividades inadequadas.

Observações:

VI. RESPOSTA A MUDANÇAS

1 Respostas à mudança adequadas a idade: Embora a criança possa perceber ou comentar as mudanças na rotina, ela é capaz de aceitar estas mudanças sem angústia excessiva.

1.5

2 Respostas à mudança adequadas à idade levemente anormal: Quando um adulto tenta mudar tarefas, a criança pode continuar na mesma atividade ou usar os mesmos materiais.

2.5

3 Respostas à mudança adequadas à idade moderadamente anormal: A criança resiste ativamente a mudanças na rotina, tenta continuar sua antiga atividade e é difícil de distraí-la. Ela pode tornar-se infeliz e zangada quando uma rotina estabelecida é alterada.

3.5

4 Respostas à mudança adequadas à idade gravemente anormal: A criança demonstra reações graves às mudanças. Se uma mudança é forçada, ela pode tornar-se extremamente zangada ou não disposta a ajudar e responder com acessos de raiva.

Observações:

VII. RESPOSTA VISUAL

1 Resposta visual adequada: O comportamento visual da criança é normal e adequado para sua idade. A visão é utilizada em conjunto com outros sentidos como forma de explorar um objeto novo.

1.5

2 Resposta visual levemente anormal: A criança precisa, ocasionalmente, ser lembrada de olhar para os objetos. A criança pode estar mais interessada em olhar espelhos ou luzes do que o fazem seus pares, pode ocasionalmente olhar fixamente para o espaço, ou pode evitar olhar as pessoas nos olhos.

2.5

3 Resposta visual moderadamente anormal: A criança deve ser lembrada freqüentemente de olhar para o que está fazendo, ela pode olhar fixamente para o espaço, evitar olhar as pessoas nos olhos, olhar objetos de um ângulo incomum ou segurar os objetos muito próximos aos olhos.

3.5

4 Resposta visual gravemente anormal: A criança evita constantemente olhar para as pessoas ou para certos objetos e pode demonstrar formas extremas de outras peculiaridades visuais descritas acima.

Observações:

VIII. RESPOSTA AUDITIVA

**1**	Respostas auditivas adequadas para a idade: O comportamento auditivo da criança é normal e adequado para idade. A audição é utilizada junto com outros sentidos.
1.5	
**2**	Respostas auditivas levemente anormal: Pode haver ausência de resposta ou uma resposta levemente exagerada a certos sons. Respostas a sons podem ser atrasadas e os sons podem necessitar de repetição para prender a atenção da criança. A criança pode ser distraída por sons externos.
2.5	
**3**	Respostas auditivas moderadamente anormal: As repostas da criança aos sons variam. Freqüentemente ignora o som nas primeiros vezes em que é feito. Pode assustar-se ou cobrir as orelhas ao ouvir alguns sons do cotidiano.
3.5	
**4**	Respostas auditivas gravemente anormal: A criança reage exageradamente e/ou ou despreza sons num grau extremamente significativo, independente do tipo de som.

Observações:

IX. RESPOSTA E USO DO PALADAR, OLFATO E TATO

**1**	Uso e reposta normais do paladar, olfato e tato: A criança explora novos objetos de um modo adequado a sua idade, geralmente sentindo ou olhando. Paladar ou olfato podem ser usados quando adequados. Ao reagir a pequenas dores do dia-a-dia, a criança expressa desconforto mas não reage exageradamente.
1.5	
**2**	Uso e reposta levemente anormais do paladar, olfato e tato: A criança pode persistir em colocar objetos na boca; pode cheirar ou provar/experimentar objetos não comestíveis. Pode ignorar ou ter reação levemente exagerada à uma dor mínima, para a qual uma criança normal expressaria somente desconforto.
2.5	
**3**	Uso e resposta moderadamente anormais do paladar, olfato e tato: A criança pode estar moderadamente preocupada em tocar, cheirar ou provar objetos ou pessoas. A criança pode reagir demais ou muito pouco.
3.5	
**4**	Uso e resposta gravemente anormais do paladar, olfato e tato: A criança está preocupada em cheirar, provar e sentir objetos, mais pela sensação do que pela exploração ou uso normal dos objetos. A criança pode ignorar completamente a dor ou reagir muito fortemente a desconfortos leves.

Observações:

X. MEDO OU NERVOSISMO

**1**	Medo ou nervosismo normais: O comportamento da criança é adequado tanto à situação quanto à idade
1.5	
**2**	Medo ou nervosismo levemente anormais: A criança ocasionalmente demonstra muito ou pouco medo ou nervosismo quando comparada às reações de uma criança normal da mesma idade e em situação semelhante.
2.5	

3 Medo ou nervosismo moderadamente anormais: A criança demonstra bastante mais ou bastante menos medo do que seria típico para uma criança mais nova ou mais velha em uma situação similar.

3.5

4 Medo ou nervosismo gravemente anormais: Medos persistem mesmo após experiências repetidas com eventos ou objetos inofensivos. É extremamente difícil acalmar ou confortar a criança. A criança pode, por outro lado, falhar em demonstrar consideração adequada aos riscos que outras crianças da mesma idade evitam.

Observações:

XI. COMUNICAÇÃO VERBAL

1 Comunicação verbal normal, adequada a idade e à situação.

1.5

2 Comunicação verbal levemente anormal: A fala demonstra um atraso global. A maior parte do discurso tem significado; porém, alguma ecolalia ou inversão pronominal podem ocorrer. Algumas palavras peculiares ou jargões podem ser usados ocasionalmente.

2.5

3 Comunicação verbal moderadamente anormal: A fala pode estar ausente. Quando presente, a comunicação verbal pode ser uma mistura de alguma fala significativa e alguma linguagem peculiar, tais como jargão, ecolalia ou inversão pronominal. As peculiaridades na fala significativa podem incluir questionamentos excessivos ou preocupação com algum tópico em particular.

3.5

4 Comunicação verbal gravemente anormal: Fala significativa não é utilizada. A criança pode emitir gritos estridentes e infantis, sons animais ou bizarros, barulhos complexos semelhantes à fala, ou pode apresentar o uso bizarro e persistente de algumas palavras reconhecíveis ou frases.

Observações:

XII. COMUNICAÇÃO NÃO-VERBAL

1 Uso normal da comunicação não-verbal adequado à idade e situação

1.5

2 Uso da comunicação não-verbal levemente anormal: Uso imaturo da comunicação não-verbal; a criança pode somente apontar vagamente ou esticar-se para alcançar o que quer, nas mesmas situações nas quais uma criança da mesma idade pode apontar ou gesticular mais especificamente para indicar o que deseja.

2.5

3 Uso da comunicação não-verbal moderadamente anormal: A criança geralmente é incapaz de expressar suas necessidades ou desejos de forma não verbal, e não consegue compreender a comunicação não-verbal dos outros.

3.5

4 Uso da comunicação não-verbal gravemente anormal: A criança utiliza somente gestos bizarros ou peculiares, sem significado aparente, e não demonstra nenhum conhecimento do significados associados aos gestos ou expressões faciais dos outros.

Observações:

XIII. NÍVEL DE ATIVIDADE

1 Nível de atividade normal para idade e circunstâncias: A criança não é nem mais nem menos ativa que uma criança normal da mesma idade em uma situação semelhante.

1.5

2 Nível de atividade levemente anormal: A criança pode tanto ser um pouco irrequieta quanto um pouco –preguiçosa‖ , apresentando, algumas vezes, movimentos lentos. O nível de atividade da criança interfere apenas levemente no seu desempenho.

2.5

3 Nível de atividade moderadamente anormal: A criança pode ser bastante ativa e difícil de conter. Ela pode ter uma energia ilimitada ou pode não ir prontamente para a cama à noite. Por outro lado, a criança pode ser bastante letárgica e necessitar de um grande estímulo para mover-se.

3.5

4 Nível de atividade gravemente anormal: A criança exibe extremos de atividade ou inatividade e pode até mesmo mudar de um extremo ao outro.

Observações:

XIV. NÍVEL E CONSISTÊNCIA DA RESPOSTA INTELECTUAL

1 A inteligência é normal e razoavelmente consistente em várias áreas: A criança é tão inteligente quanto crianças típicas da mesma idade e não tem qualquer habilidade intelectual ou problemas incomuns.

1.5

2 Funcionamento intelecual levemente anormal: A criança não é tão inteligente quanto crianças típicas da mesma idade; as habilidades apresentam-se razoavelmente regulares através de todas as áreas.

2.5

3 Funcionamento intelectual moderadamente anormal: Em geral, a criança não é tão inteligente quanto uma típica criança da mesma idade, porém, a criança pode funcionar próximo do normal em uma ou mais áreas intelectuais.

3.5

4 Funcionamento intelectual gravemente anormal: Embora a criança geralmente não seja tão inteligente quanto uma criança típica da mesma idade, ela pode funcionar até mesmo melhor que uma criança normal da mesma idade em uma ou mais áreas.

Observações:

	XV. IMPRESSÕES GERAIS
**1**	Sem autismo: a criança não apresenta nenhum dos sintomas característicos do autismo.
1.5	
**2**	Autismo leve: A criança apresenta somente um pequeno número de sintomas ou somente um grau leve de autismo.
2.5	
**3**	Autismo moderado: A criança apresenta muitos sintomas ou um grau moderado de autismo.
3.5	
**4**	Autismo grave: a criança apresenta inúmeros sintomas ou um grau extremo de autismo
	**Observações:**

Escore por categoria

**I**	**II**	**III**	**IV**	**V**	**VI**	**VII**	**VIII**	**IX**	**X**	**XI**	**XII**	**XIII**	**XIV**	**XV**	**Total**

Resultado:

15-30: sem autismo

30-36: autismo leve-moderado

36-60: autismo grave

ANEXO 2 – ACSF-SC

Instruções do instrumento ASCF:SC

PASSO 1
Por favor, leia o **Guia do Usuário do Instrumento ACSF:SC** antes de começar.

PASSO 2
Por favor, revise as descrições dos 5 níveis e suas distinções no **Instrumento ACSF:SC**.

PASSO 3
Pensando sobre o último mês, quais são as melhores habilidades de comunicação social que você observou essa criança fazer (mesmo que tenha sido observada apenas uma vez). Isso é chamado Capacidade.

O **Nível de Capacidade** da criança é______________________.

PASSO 4
A seguir revise o instrumento e, novamente, pensando sobre o último mês, quais foram as habilidades de comunicação social que você observou a criança fazendo mais consistentemente? Isso é chamado de Desempenho Típico.

O **Nível de Desempenho Típico** da criança é__________________.

- Os comportamentos de comunicação social da criança no último mês **devem corresponder ou se parecer com a caracterização geral** que é descrita no nível. Se a criança não corresponde à caracterização descrita ela deve ser classificada no nível de habilidade mais baixo.
- Se as habilidades de funcionalidade de comunicação social são **inferiores ao Nível V, classifique-a como V. Da mesma forma, se elas são superiores ao Nível I, classifique-a como I.**

Não se esqueça

Existe uma variedade de maneiras pelas quais as as crianças iniciam comunicação ou respondem à comunicação de outras pessoas, tais como:

expressões faciais, movimentos corporais ou gestos, linguagem de sinais, contato visual e uso de fixação visual para direcionar a atenção de outras pessoas, tecnologia, equipamentos ou ferramentas de CAA (exemplos: PECS, iPad, álbum de fotos, scrapbooks, dispositivos geradores de fala), e fala.

ACSF:SC
INSTRUMENTO

Tradução Português Brasileiro

Sistema de Classificação de Funcionalidade no Autismo: Comunicação Social
Version 2016

Tradução português brasileiro por:
Ana Amélia Cardoso*
Samara Costa, Adriana Queiroz,
Carla Ribeiro Lage, Cecília Pletschette Galvão
*Departamento de Terapia Ocupacional, Universidade Federal de Minas Gerais,
(anaameliato@eeffto.ufmg.br)

Por favor, consulte o Guia do Usuário do **ACSF:SC** e as Instruções do **ACSF:SC** antes de revisar os 5 níveis descritos dentro deste folheto.

Autism Classification System of Functioning: Social Communication
ACSF:SC Tool© 2016
Briano Di Rezze, Lonnie Zwaigenbaum, Mary Jo Cooley Hidecker,
Martha Cousins, Peter Szatmari, Mary Law, Paul Stratford, Peter Rosenbaum

CanChild Centre for Childhood Disability Research,
McMaster University, Hamilton, ON
www.canchild.ca

Nível V – No último mês, uma criança no nível V pode ter sido observada ...

Brincando com objetos ou falando consigo mesma.

Tentando iniciar ou reagir a palavras ou ações físicas específicas de outra pessoa. O objetivo de sua comunicação pode ser entendido apenas pelo seu cuidador primário ou professor/ terapeuta altamente experimente.

Nível IV – No último mês, uma criança no nível IV tem sido observada ...

Tentando iniciar comunicação com seu(s) cuidador(es) primário(s), solicitando ter suas necessidades atendidas.

Tentando responder a comunicação iniciada por pessoas que ela conhece (pode ser algo tão simples quanto o uso de uma expressão facial), mas pode não estar respondendo a pessoas que ela não conhece.

Nível III - No último mês, uma criança no nível III tem sido observada ...

Iniciando comunicação com pessoas que ela conhece, principalmente para solicitar que tenha suas necessidades atendidas.

Tentando iniciar comunicação com objetivos sociais usando solicitações simples, praticadas ou roteirizadas (verbalmente ou não verbalmente) sobre seus interesses/atividades preferidos.

Respondendo a comunicação de outras pessoas (como quando perguntado sobre questões simples como "O que é isso?"), mas a comunicação não é sustentada.

Nível II - No último mês, uma criança no nível II tem sido observada ...

Iniciando ou respondendo para se comunicar com objetivos sociais sobre seus interesses/ atividades preferidos, com a maioria das pessoas.

Sustentando comunicação até a outra pessoa mudar o assunto/ atividade ou até não estar sendo compreendido.

Nível I - No último mês, uma criança no nível I tem sido observada ...

Iniciando e respondendo para se comunicar com objetivos sociais sobre mais do que somente seus interesses/atividades preferidos, com a maioria das pessoas.

Sustentando comunicação com a maioria das pessoas. Apesar de poder ter alguma dificuldade, ela vai tentar responder à mudança no assunto/ atividade ou usar estratégias efetivas de comunicação para ser compreendida.

Distinções entre os Níveis

Distinção entre nível V e nível IV

Uma criança no Nível V está simplesmente REAGINDO à comunicação de outras pessoas & o objetivo de sua comunicação na melhor das hipóteses é conhecido apenas pelo cuidador primário ou professor/terapeuta altamente experiente,

***enquanto** uma criança no Nível IV está TENTANDO iniciar por necessidade própria e TENTANDO responder a pessoas que ela conhece.*

Distinção entre nível IV e nível III

Uma criança no Nível IV pode estar TENTANDO iniciar e responder às pessoas que ela conhece, para ter suas necessidades atendidas,

***equanto** uma criança no Nível III está fazendo essas coisas por sua própria necessidade, bem como TENTANDO iniciar a comunicação com objetivos sociais sobre seus interesses preferidos. Ela pode responder à solicitação de outras pessoas, mas a comunicação é roteirizada e não é facilmente sustentada.*

Distinção entre nível III e nível II

Uma criança no nível III está TENTANDO iniciar e responder com objetivos sociais sobre seus interesses/atividades preferidos,

***enquanto** uma criança no Nível II está iniciando e respondendo, com objetivos sociais, a maioria das pessoas, que podem continuar a interação. Entretanto, se alguma coisa muda, ou ela não é compreendida, a comunicação não dura.*

Distinção entre nível II e nível I

Uma criança no Nível II está se comunicando com outras pessoas com objetivos sociais, mas tem problemas para sustentar a interação se existirem mudanças,

***enquanto**, embora a interação possa não parecer perfeita, uma criança no Nível I tenta sustentar a interação usando estratégias efetivas de comunicação para ser compreendida, e se adaptar a mudanças*

ANEXO 3 –PEM-CY

FOR OFFICE USE ONLY: ____________ (ID)

Participation and Environment Measure – Children and Youth©
(Medida da Participação e do Contexto – Crianças e Jovens)

Wendy Coster, Mary Law, Gary Bedell

Permissão concedida para reproduzir a Medida da Participação e do Contexto—Crianças e Jovens (Participation and Environment Measure—Children and Youth, PEM-CY) em páginas inteiras com informação de copyright, para investigação e prática clínica e não para revenda. Modificações aos itens ou estrutura da PEM-CY, assim como traduções para outros idiomas, não podem ser realizadas sem permissão escrita dos autores.

© Copyright 2010 Trustees of Boston University
Tradução Portuguesa (2012), Susana Martins (susanaisabelmartins@gmail.com) e Manuela Sanches Ferreira, Escola Superior de Educação do Porto, Portugal

INSTRUÇÕES PARA PESQUISA

Participação refere-se ao envolvimento da criança em atividades importantes do quotidiano, em casa, na escola e na comunidade. O significado de participação inclui com que frequência a criança faz as atividades, E o quão envolvida está quando faz essas atividades.

O inquérito coloca uma série de questões acerca da participação da criança em 25 tipos de atividades que ocorrem em três contextos: casa, escola e comunidade. Apresentamos alguns exemplos para ilustrar cada tipo de atividade. No entanto, deve pensar em todas as atividades que pertencem a essa categoria quando responde a essas questões.

Para cada tipo de atividade perguntamos:

1. com que frequência a sua criança participou ao longo dos últimos 4 meses
2. quão envolvida está a sua criança quando participa em 1 ou 2 atividades deste tipo que, ele ou ela, faça com mais frequência
3. se gostaria que a participação da sua criança mudasse (ou não), se sim, como gostaria que mudasse

IMPORTANTE

Este inquérito não pergunta acerca do nível de independência da sua criança quando participa nas atividades. "Envolvimento" refere-se ao quão empenhada a sua criança está na atividade, usando que apoios, ajudas, adaptações, ou métodos que use regularmente ou que tenha disponível.

Quando selecionar a sua resposta, por favor pense acerca do nível de atenção, concentração, empenho emocional, ou satisfação da sua criança (considerando o uso de suportes ou ajudas que estão geralmente disponíveis).

Muito envolvida = De forma geral, a criança está empenhada durante a atividade. Mostra muita iniciativa e/ou interesse e atenção ao que ele ou ela e outros estão a fazer durante a atividade.

Algo envolvido = A criança está empenhada na atividade durante algum tempo. Mostra alguma iniciativa e/ou interesse e atenção ao que ele ou ela e outros estão a fazer durante a atividade.

Minimamente envolvido = A criança está empenhada uma pequena parte do tempo da atividade. Mostra pouca iniciativa e/ou interesse e atenção ao que ele ou ela e outros estão a fazer durante a atividade.

Se existem aspetos que ajudam ou tornam a participação da sua criança mais difícil, tais como equipamentos ou apoio de outros, pode dizer-nos acerca do seu impacto nas secções de contexto doméstico, contexto escolar e contexto comunitário deste inquérito.

© Copyright 2010 Trustees of Boston University
Tradução Portuguesa (2012), Susana Martins (susanaisabelmartins@gmail.com) e Manuela Sanches Ferreira, Escola Superior de Educação do Porto, Portugal

Participação em CASA

A) Tipicamente, com que frequência a sua criança participa nas atividades apresentadas ou parecidas, dentro de cada uma das categorias abaixo indicadas?

MARQUE UMA RESPOSTA ☑

B) Pense em cada uma das atividades ou parecidas, dentro de cada uma das categorias abaixo indicadas, em que a sua criança participe com mais frequência. Tipicamente, quão envolvida esta a sua criança quando faz essas atividades?

MARQUE UMA RESPOSTA ☑

C) Gostaria que a participação da sua criança mudasse neste tipo de atividade?

SE SIM, MARQUE TODAS AS QUE SE APLICAM ☑

	Diariamente	Algumas vezes por semana	Uma vez por semana	Algumas vezes por mês	Uma vez por mês	Algumas vezes nos últimos 4 meses	Uma vez nos últimos 4 meses	Nunca (saltar para a questão C)	5 Muito Envolvido	4	3 Algo Envolvido	2	1 Minimamente Envolvido	Não desejo mudança	Sim, fazer mais vezes	Sim, fazer menos vezes	Sim, estar mais Envolvido	Sim, estar menos Envolvido	Sim, estar Envolvido numa maior variedade de atividades
**1) Jogos de computador e consolas**																			
**2) Jogos e brincadeiras no interior** (ex. brincar com brinquedos, puzzles, jogos de tabuleiro, brincar às cozinhas ou jogos de faz de conta)																			
**3) Artes, trabalhos manuais, música e passatempos** (ex. fazer trabalhos manuais e de arte, ouvir música, tocar um instrumento, colecionar, ler por prazer, cozinhar por gosto)																			
**4) Ver TV, vídeos e DVDs**																			
**5) Estar com outras pessoas** (ex. interagir com pares, familiares, hóspedes)																			
**6) Socializar usando tecnologias** (ex. telefone, computador)																			
**7) Tarefas domésticas** (ex. pôr/tirar louça da máquina de lavar louça, limpar o quarto ou outras áreas da casa, cozinhar, levar o lixo, pôr a mesa, cuidar de animal doméstico)																			
**8) Cuidados pessoais** (ex. vestir-se, escolher a roupa, escovar o cabelo e dentes, colocar maquiagem)																			
**9) Preparação para a escola (não trabalho de casa)** (ex. reunir materiais, preparar a mochila, colocar lanche na mochila, rever horário)																			
**10) Trabalhos de casa** (ex. leituras diárias, trabalhos para casa, projectos escolares)																			

© Copyright 2010 Trustees of Boston University

Tradução Portuguesa (2012), Susana Martins (susanaisabelmartins@gmail.com) e Manuela Sanches Ferreira, Escola Superior de Educação do Porto, Portugal

Contexto de CASA

Os seguintes aspetos <u>ajudam ou tornam mais difícil</u> a participação da sua criança em atividades em casa? SELECIONE UMA RESPOSTA ☑	Não é um problema	Geralmente ajuda	Às vezes ajuda; às vezes dificulta	Geralmente torna mais difícil
1. A disposição física ou a quantidade de espaço e mobília em sua casa				
2. As qualidades sensoriais do contexto doméstico (ex. quantidade e/ou tipo de som, luz, temperatura, textura dos objetos)				
3. As exigências físicas das atividades típicas do contexto doméstico (ex. força, resistência, coordenação)				
4. As exigências cognitivas das atividades típicas do contexto doméstico (ex. concentração, atenção, resolução de problemas)				
5. As exigências sociais das atividades típicas do contexto doméstico (ex. comunicação, interação com outros)				
6. O relacionamento da sua criança com os elementos da família em casa (ex. irmãos mais novos, pais, avós)				
7. As atitudes e ações das babysitters, terapeutas e outros profissionais que cuidam da sua criança em contexto doméstico				

SELECIONE UMA RESPOSTA ☑	Não é necessário	Geralmente, sim	Às vezes sim; às vezes não	Geralmente, não
8. Há em sua casa serviços disponíveis e/ou adequados para apoiar a participação da sua criança?				

© Copyright 2010 Trustees of Boston University
Tradução Portuguesa (2012), Susana Martins (susanaisabelmartins@gmail.com) e Manuela Sanches Ferreira, Escola Superior de Educação do Porto, Portugal

Contexto de CASA

Os seguintes estão disponíveis/ ou adequados para apoiar a participação da sua criança em casa? SELECIONE UMA RESPOSTA ☑	Geralmente, sim	Às vezes sim; às vezes não	Geralmente, não
9. Materiais em casa (ex. equipamento desportivo, material de trabalhos manuais, material de leitura, dispositivos de auxílio e tecnologias, horários de imagens ou palavras)			
10. Informação (ex. acerca de atividades, serviços, programas)			
11. Tem (ou a sua família) tempo suficiente para apoiar a participação da criança em casa?			
12. Tem (ou a sua família) dinheiro suficiente para apoiar a participação da criança em casa?			

Quais algumas das coisas que faz, ou outros elementos da família, que ajudam a sua criança a participar com sucesso nas atividades em casa? POR FAVOR LISTE ATÉ 3 ESTRATÉGIAS
1.
2.
3.

© Copyright 2010 Trustees of Boston University
Tradução Portuguesa (2012), Susana Martins (susanaisabelmartins@gmail.com) e Manuela Sanches Ferreira, Escola Superior de Educação do Porto, Portugal

Participação na ESCOLA

A) Tipicamente, com que <u>frequência</u> a sua criança participa nas <u>atividades apresentadas ou parecidas</u>, dentro de cada uma das categorias abaixo indicadas?

MARQUE UMA RESPOSTA ☑

B) Pense em <u>cada uma das atividades ou parecidas</u>, dentro de cada uma das categorias abaixo indicadas, em que a sua criança participe com mais frequência. Tipicamente, <u>quão envolvida</u> está a sua criança quando faz essas atividades?

MARQUE UMA RESPOSTA ☑

C) Gostaria que a participação da sua criança <u>mudasse</u> neste tipo de atividade?

SE SIM, MARQUE TODAS AS QUE SE APLICAM ☑

	Diariamente	Algumas vezes por semana	Uma vez por semana	Algumas vezes por mês	Uma vez por mês	Algumas vezes nos últimos 4 meses	Uma vez nos últimos 4 meses	Nunca (Ir para a questão C)	5 Muito Envolvido	4	3 Algo Envolvido	2	1 Minimamente Envolvido	Mudança não desejada	Sim, fazer mais vezes	Sim, fazer menos vezes	Sim, estar mais Envolvido	Sim, estar menos Envolvido	Sim, estar Envolvido numa maior variedade de atividades
**1) Atividades em sala de aula** (ex. trabalho de grupo, discussões na turma, testes, trabalhos na sala)																			
**2) Excursões e eventos na escola** (ex. ir ao museu, feira escolar, concerto ou peça, danças, angariação de fundos)																			
**3) Equipas dinamizadas pela escola, clubes e organizações** (ex. grupos, clubes, equipas, associação de estudantes)																			
**4) Estar com os colegas fora da sala** (ex. estar com os colegas no almoço, no intervalo ou em outras pausas do dia escolar)																			
**5) Papéis específicos na escola** (ex. tutor de outro aluno, delegado de turma)																			

© Copyright 2010 Trustees of Boston University
Tradução Portuguesa (2012), Susana Martins (susanaisabelmartins@gmail.com) e Manuela Sanches Ferreira, Escola Superior de Educação do Porto, Portugal

Contexto ESCOLAR

Os seguintes aspetos <u>ajudam ou tornam mais difícil</u> a participação da sua criança em atividades na escola? SELECIONE UMA RESPOSTA ☑	Não é um problema	Geralmente ajuda	Às vezes ajuda; às vezes dificulta	Geralmente torna mais difícil
1. A disposição física ou a quantidade de espaço na sala de aula, recreio, ou em outras áreas do edifício escolar (ex. presença de passeios, disponibilidade de rampas ou elevadores no edifício escolar)				
2. As qualidades sensoriais do contexto escolar (ex. barulho, multidões, iluminação, etc.)				
3. Condições atmosféricas exteriores (ex. temperatura, clima)				
4. As exigências físicas das atividades escolares (ex. força, resistência, coordenação)				
5. As exigências cognitivas das atividades típicas do contexto escolar (ex. concentração, atenção, resolução de problemas)				
6. As exigências sociais das atividades típicas do contexto escolar (ex. comunicação, interação com outros)				
7. Atitudes e ações de professores, treinadores ou pessoal da escola face à sua criança				
8. O relacionamento da sua criança com os colegas				
9. A segurança da escola (ex. supervisão, crime, violência)				

Os seguintes estão disponíveis/ ou adequados para apoiar a participação da sua criança na escola? SELECIONE UMA RESPOSTA ☑	Não é necessário	Geralmente, sim	Às vezes sim; às vezes não	Geralmente, não
10. Acesso a transporte pessoal para ir para a escola (ex. carro familiar ou bicicleta)				
11. Acesso a transportes públicos para ir para a escola (ex. autocarro, comboio, metro)				
12. Programas e serviços (ex. depois da escola, recreativos, recursos especiais, ajudas/assistentes educacionais)				
13. Políticas e procedimentos relacionados com a escola (ex. critérios de elegibilidade para serviços, regras de comportamento)				

© Copyright 2010 Trustees of Boston University
Tradução Portuguesa (2012), Susana Martins (susanaisabelmartins@gmail.com) e Manuela Sanches Ferreira, Escola Superior de Educação do Porto, Portugal

Contexto ESCOLAR

Os seguintes estão disponíveis/ ou adequados para apoiar a participação da sua criança na escola? SELECIONE UMA RESPOSTA ☑	Geralmente, sim	Às vezes sim; às vezes não	Geralmente, não
14. Materiais (ex. dispositivos de auxílio ou tecnologias, material de leitura, equipamento desportivo, material de trabalhos manuais)			
15. Informação (ex. acerca de atividades, serviços, programas)			
16. Tem (ou a sua família) tempo suficiente para apoiar a participação da criança na escola?			
17. Tem (ou a sua família) dinheiro suficiente para apoiar a participação da criança na escola?			

Quais algumas das coisas que faz, ou outros elementos da família, que ajudam a sua criança a participar com sucesso nas atividades na escola? POR FAVOR LISTE ATÉ 3 ESTRATÉGIAS
1.
2.
3.

© Copyright 2010 Trustees of Boston University
Tradução Portuguesa (2012), Susana Martins (susanaisabelmartins@gmail.com) e Manuela Sanches Ferreira, Escola Superior de Educação do Porto, Portugal

Participação na COMUNIDADE

A) Tipicamente, com que frequência a sua criança participa nas atividades apresentadas ou parecidas, dentro de cada uma das categorias abaixo indicadas?

MARQUE UMA RESPOSTA ☑

B) Pense em cada uma das atividades ou parecidas, dentro de cada uma das categorias abaixo indicadas, em que a sua criança participe com mais frequência. Tipicamente, quão envolvida está a sua criança quando faz essas atividades?

MARQUE UMA RESPOSTA ☑

C) Gostaria que a participação da sua criança mudasse neste tipo de atividade?

SE SIM, MARQUE TODAS AS QUE SE APLICAM ☑

	A) Diariamente	A) Algumas vezes por semana	A) Uma vez por semana	A) Algumas vezes por mês	A) Uma vez por mês	A) Algumas vezes nos últimos 4 meses	A) Uma vez nos últimos 4 meses	A) Nunca (ir para a questão C)	B) 5 Muito Envolvido	B) 4	B) 3 Algo Envolvido	B) 2	B) 1 Minimamente Envolvido	C) Mudança não desejada	C) Sim, fazer mais vezes	C) Sim, fazer menos vezes	C) Sim, estar mais Envolvido	C) Sim, estar menos Envolvido	C) Sim, estar Envolvido numa maior variedade de atividades
**1) Saídas na vizinhança** (ex. fazer compras na loja/ centro comercial, ir ao cinema, ir ao restaurante, ir à livraria ou biblioteca local)																			
**2) Eventos na comunidade** (ex. ir a uma peça, concerto, eventos desportivos, desfile)																			
**3) Atividades físicas organizadas** (ex. desportos em equipa ou treinos tais como futebol, hóquei, artes marciais, dança, equitação, natação, ginástica)																			
**4) Atividades físicas não estruturadas** (ex. caminhadas na natureza, andar de bicicleta, patins em linha, andar de skate, brincar às escondidas ou apanha, "dar uns toques" com bola)																			
**5) Aulas e cursos (não escolares)** (ex. música, arte, outras línguas, computadores)																			

© Copyright 2010 Trustees of Boston University
Tradução Portuguesa (2012), Susana Martins (susanaisabelmartins@gmail.com) e Manuela Sanches Ferreira, Escola Superior de Educação do Porto, Portugal

Participação na COMUNIDADE

A) Tipicamente, com que frequência a sua criança participa nas atividades apresentadas ou parecidas, dentro de cada uma das categorias abaixo indicadas? MARQUE UMA RESPOSTA ☑

B) Pense em cada uma das atividades ou parecidas, dentro de cada uma das categorias abaixo indicadas, em que a sua criança participe com mais frequência. Tipicamente, quão envolvida está a sua criança quando faz essas atividades? MARQUE UMA RESPOSTA ☑

C) Gostaria que a participação da sua criança mudasse neste tipo de atividade? SE SIM, MARQUE TODAS AS QUE SE APLICAM ☑

	A) Diariamente	A) Algumas vezes por semana	A) Uma vez por semana	A) Algumas vezes por mês	A) Uma vez por mês	A) Algumas vezes nos últimos 4 meses	A) Uma vez nos últimos 4 meses	A) Nunca (ir para a questão C)	B) 5 Muito Envolvido	B) 4	B) 3 Algo Envolvido	B) 2	B) 1 Minimamente Envolvido	C) Mudança não desejada	C) Sim, fazer mais vezes	C) Sim, fazer menos vezes	C) Sim, estar mais Envolvido	C) Sim, estar menos Envolvido	C) Sim, estar Envolvido numa maior variedade de atividades
6) Organizações, grupos, clubes e atividades de voluntariado ou liderança (ex. escuteiros, grupos de jovens, associações)																			
7) Encontros e atividades religiosas ou espirituais (ex. ir à igreja ou templo, aulas de religião-catequese, grupos)																			
8) Estar com outras crianças da comunidade (ex. sair com amigos, encontros informais fora do contexto de casa ou escola)																			
9) Trabalho remunerado (ex. babysitting, trabalhar numa loja, fazer tarefas ou recados a troco de dinheiro/ pagamento)																			
10) Viagens ou visitas em que passa noite fora (ex. dormir em casa de familiares ou amigos, férias, acampamentos)																			

© Copyright 2010 Trustees of Boston University
Tradução Portuguesa (2012), Susana Martins (susanaisabelmartins@gmail.com) e Manuela Sanches Ferreira, Escola Superior de Educação do Porto, Portugal

Contexto da COMUNIDADE

Os seguintes aspectos ajudam ou tornam mais difícil a participação da sua criança em atividades na comunidade? SELECIONE UMA RESPOSTA ☑	Não é um problema	Geralmente ajuda	Às vezes ajuda; às vezes dificulta	Geralmente torna mais difícil
1. A disposição física ou a quantidade de espaço no exterior e interior dos edifícios (ex. distância até às lojas, existência de passeios, existência de rampas ou elevadores)				
2. As qualidades sensoriais dos contextos comunitários (ex. barulho, multidões, iluminação)				
3. As exigências físicas de atividades típicas (ex. força, resistência, coordenação)				
4. As exigências cognitivas das atividades típicas (ex. concentração, atenção, resolução de problemas)				
5. As exigências sociais das atividades típicas (ex. comunicação, interação com outros)				
6. O relacionamento da sua criança com os colegas				
7. Atitudes e ações de outros membros da comunidade face à sua criança (ex. lojistas, instrutores, treinadores, outros familiares)				
8. Condições atmosféricas exteriores (ex. temperatura, clima)				
9. A segurança da comunidade (ex. tráfico, crime, violência)				

Os seguintes estão disponíveis/ ou adequados para apoiar a participação da sua criança na comunidade? SELECIONE UMA RESPOSTA ☑	Não é necessário	Geralmente, sim	Às vezes sim; às vezes não	Geralmente, não
10. Acesso a transporte pessoal para ir a atividades na comunidade (ex. carro familiar ou bicicleta)				
11. Acesso a transportes públicos para ir a atividades na comunidade (ex. autocarro, comboio, metro)				
12. Programas e serviços (ex. programas de desporto inclusivo, assistentes de apoio pessoal)				

© Copyright 2010 Trustees of Boston University
Tradução Portuguesa (2012), Susana Martins (susanaisabelmartins@gmail.com) e Manuela Sanches Ferreira, Escola Superior de Educação do Porto, Portugal

Contexto da COMUNIDADE

Os seguintes estão disponíveis/ ou adequados para apoiar a participação da sua criança na comunidade?<br><br>SELECIONE UMA RESPOSTA ☑	Geralmente, sim	Às vezes sim; às vezes não	Geralmente, não
13. Informação (ex. acerca de atividades, serviços, programas)			
14. Equipamentos ou material (ex. equipamento desportivo, material de trabalhos manuais, material de leitura, dispositivos de auxílio ou tecnologias)			
15. Tem (ou a sua família) tempo suficiente para apoiar a participação da criança na comunidade?			
16. Tem (ou a sua família) dinheiro suficiente para apoiar a participação da criança na comunidade?			

Quais algumas das coisas que faz, ou outros elementos da família, que ajudam a sua criança a participar com sucesso nas atividades na comunidade?<br><br>POR FAVOR LISTE ATÉ 3 ESTRATÉGIAS
1.
2.
3.

© Copyright 2010 Trustees of Boston University
Tradução Portuguesa (2012), Susana Martins (susanaisabelmartins@gmail.com) e Manuela Sanches Ferreira, Escola Superior de Educação do Porto, Portugal

ANEXO 4 – TESTE DESENVOLVIMENTO MOTOR GROSSO – SEGUNDA EDIÇÃO

TGMD-2: Sub teste: Habilidades de locomoção

Habilidade Motora	Materiais	Descrição	Critérios de êxito	1ª	2ª	Escore
Corrida "Vamos correr o mais rápido possível"	18 metros de espaço livre, 2 cones e fita adesiva.	Dois cones (duas linhas) distanciados 15 metros entre si. Dizer à criança para correr o mais rápido possível entre os cones após o sinal.	1. Os braços movem-se em oposição às pernas e com os braços fletidos.			
			2. Existe uma breve fase aérea em que ambos os pés não contactam o solo.			
			3. Não apoia todo o pé simultaneamente. Contacta primeiro no chão com o calcanhar ou a ponta do pé.			
			4. A perna livre flete aproximadamente 90° (perto das nádegas).			
			Escore da habilidade			
Galope "Vamos andar de cavalinho"	8 metros de espaço livre, fita adesiva ou dois cones.	Marcar uma distância de 8 metros com os cones ou a fita adesiva. Dizer à criança para galopar de um cone ao outro. Na segunda tentativa galopar em direção ao 1° cone.	1. Braços fletidos e ao nível da cintura durante a saída do solo.			
			2. Um passo em frente com o pé dominante seguido de um passo do pé não dominante até a uma posição adjacente ou atrás do pé dominante.			
			3. Existe um período de tempo aéreo em que os pés se encontram fora do chão.			
			4. Mantêm um padrão rítmico em quatro galopes consecutivos.			
			Escore da habilidade			
Salto com um pé "Vamos pular igual o saci quatro vezes com um perna" (Depois volta com a outra perna)	No mínimo 5 metros de espaço livre.	Dizer à criança para realizar três saltos com o seu pé dominante e depois com o outro pé. Repete duas vezes.	1. A perna livre oscila para frente num movimento pendular para produzir força.			
			2. O pé da perna livre permanece atrás do corpo.			
			3. Braços fletidos oscilando para frente para produzir força.			
			4. Executa três vezes consecutivas com o pé dominante.			
			5. Executa três vezes consecutivas com o pé não dominante.			
			Escore da habilidade			
Passada ou saltar por cima "Vamos correr e saltar o saquinho de areia"	No mínimo 6 metros de espaço livre, um saquinho de areia e fita adesiva.	Colocar o saquinho de areia no chão. Colar uma fita adesiva no chão de modo a que fique paralela e afastada acerca de 3 m do saco de areia. A criança posiciona-se em cima da fita adesiva, corre e salta por cima do saco.	1. Salta num pé e cai com o pé oposto.			
			2. Período aéreo maior do que na corrida normal.			
			3. O braço do lado oposto ao pé de chamada vai à frente no salto.			
			Escore da habilidade			
Salto Horizontal "Vamos saltar o mais longe possível"	No mínimo 3 metros de espaço livre e fita adesiva.	Colocar uma marca de partida no chão. A criança terá de partir atrás da linha. Dizer à criança para saltar o mais longe possível. Repete duas vezes.	1. Movimento preparatório inclui a flexão dos joelhos com os braços estendidos atrás do corpo.			
			2. Braços balançam para frente e para cima atingindo a máxima extensão acima da cabeça.			
			3. Saída do solo e recepção ao solo com ambos os pés simultaneamente.			
			4. Os braços são trazidos para baixo durante a queda.			
			Escore da habilidade			
Deslocamento lateral ou corrida lateral "Vamos correr de lado em cima da linha"	No mínimo 3 metros de espaço livre, uma linha estreita e dois cones.	Colocar os dois cones separados a 7, 5 metros. Dizer à criança para deslocar-se ao longo da linha de um cone ao outro e voltar para trás. Repetir novamente.	1. O corpo permanece lateral de modo que os ombros estão alinhados com a linha do chão.			
			2. Um passo lateral com o pé de apoio do lado do deslocamento seguido de um deslocamento do outro para um ponto próximo do pé.			
			3. No mínimo realiza quatro passos consecutivos para a direita.			
			4. No mínimo realiza quatro passos consecutivos para a esquerda.			
			Escore da habilidade			

TGMD-2: Sub teste: Controle de objetos

Habilidade Motora	Materiais	Descrição	Critérios de êxito	1ª	2ª	Escore
Rebater uma bola "Vamos bater forte na bola" (Deixe a criança escolher qual o lado ela quer fazer)	Bola pequena e leve, bastão de plástico e o suporte da bola	Colocar a bola no suporte ao nível da cintura da criança. Dizer à criança para bater na bola com força. Repetir uma segunda vez	1. A mão dominante segura o bastão acima da mão não dominante.			
			2. O lado não dominante do corpo enfrenta o lançador imaginário com os pés paralelos			
			3. Rotação do tronco (cintura e ombros) durante o movimento			
			4. Transfere o peso do corpo para o pé da frente.			
			5. O bastão contacta a bola			
			Escore da habilidade			
Quicar a bola "Vamos quicar a bola quatro vezes e depois segurar"	Bola com 20 a 25 cm de diâmetro para crianças com 3-5 anos; uma bola de basquetebol para crianças com 6-10 anos	Dizer à criança para quicar a bola quatro vezes consecutivas no mesmo local, usando uma mão e termina agarrando a bola.	1. Contacta a bola com uma mão ao nível da cintura dominante			
			2. Empurra a bola com os dedos (sem bater)			
			3. A bola contacta o solo à frente ou ao lado do pé dominante			
			4. Mantém o controle da bola durante quatro dribles consecutivos sem necessitar de mexer os pés para alcança-lo.			
			Escore da habilidade			
Receber a bola "Agora você tem que pegar a bola que eu andar para você"	Uma bola de plástico com 10 cm de diâmetro; 5 metros de espaço livre e fita adesiva	Marcar duas linhas com uma distância entre si de 5 metros. A criança fica numa linha e o lançador fica na outra linha. Lançar a bola por baixo diretamente para a criança com um ligeiro arco orientando a para o seu peito. Dizer à criança para agarrar a bola com as duas mãos. A bola deve ser recebida entre os ombros e a cintura da criança.	1. Na fase inicial, as mãos encontram-se à frente do corpo e os cotovelos fletidos.			
			2. Os braços estendem-se para a bola quando esta se aproxima			
			3. A bola é agarrada apenas com as mãos			
			Escore da habilidade			
Chutar a bola "Vamos correr e chutar a bola"	Bola de plástico ou de futebol com 20 a 25 centímetros de diâmetro, saquinho de areia, 10 m de espaço livre e fita adesiva	Marcar uma linha a 10 metros da parede e outra linha a 6 metros da parede. Colocar a bola em cima do saquinho de areia que se encontra na linha mais próxima da parede. Dizer à criança para se colocar na outra linha. Dizer à criança para correr até à bola e chutar com força contra a parede.	1. Aproximação contínua e rápida à bola			
			2. Dá um passo alongado ou pequeno salto antes do contato com a bola			
			3. O pé que não pontapeia deverá ficar ao lado da linha da bola ou ligeiramente atrás da bola.			
			4. Pontapeia a bola com a parte interna do pé dominante ou com os dedos.			
			Escore da habilidade			
Arremesso da bola por cima "Vamos jogar a bola na parede lá no alto"	Bola de tênis, uma parede, fita adesiva, e 6m de espaço livre	Colar uma fita adesiva no chão a 6 metros da parede. A criança deve ficar atrás da linha de frente para a parede. Dizer à criança para lançar a bola com força contra a parede.	1. O giro é iniciado com um movimento da mão/braço para baixo			
			2. Rotação da cintura e dos ombros até o momento em que o lado não lançador se volta para direção do arremesso.			
			3. O peso é transferido como um passo para o pé oposto à mão lançadora			
			4. Movimento contínuo da mão lançadora que cruza diagonalmente para o lado oposto após lançamento.			
			Escore da habilidade			
Arremesso da bola por baixo "Vamos jogar a bola por baixo pra fazer o gol"	Bola de tênis para crianças com 3-6 anos; e uma bola pequena para crianças com 7-10 anos; dois cones; fita adesiva; 8 m de espaço livre	Colocar dois cones contra parede a uma distância entre si de 1,21 m. Colar uma fita adesiva no chão a 6 m da parede. Dizer à criança para rolar a bola com força de modo passar entre os cones.	1. A mão que lança balança para baixo e para trás do tronco enquanto o mesmo está orientado para os cones.			
			2. Passo à frente dado pelo pé oposto à mão que lança			
			3. Dobra os joelhos para se baixar.			
			4. Liberta a bola perto do chão de modo a que a bola não quique mais de 10 cm de altura.			
			Escore da habilidade			

ANEXO 5 – QUESTIONÁRIO DO PERFIL DE ALFABETIZAÇÃO FÍSICA

QUESTIONÁRIO DO PERFIL DE ALFABETIZAÇÃO FÍSICA

Nome do respondente: ______________________ Relação com a criança, adolescente ou adulto jovem: __________

Nome da criança, adolescente ou adulto jovem: ______________________ Idade: ________ Data: __________

▸ PARTE I

▸ Ele (a) utiliza algum dispositivo de tecnologia assistiva (ex.: órtese, andador, muleta, cadeira de rodas, bengalas) ou ajuda de terceiros nas seguintes situações:

Para se locomover dentro de casa? ☐ Sim ☐ Não Tipo de tecnologia ou ajuda de terceiros: __________

Para se locomover na escola? ☐ Sim ☐ Não Tipo de tecnologia ou ajuda de terceiros: __________

Para se locomover na comunidade? ☐ Sim ☐ Não Tipo de tecnologia ou ajuda de terceiros: __________

Para participar de algum esporte/atividade recreativa? ☐ Sim ☐ Não Tipo de tecnologia ou ajuda de terceiros: __________

Tipo de atividade: __________ Local: __________

▸ Ele (a) participa de algum esporte ou atividade recreativa? ☐ Sim ☐ Não Se sim, complete a tabela a seguir:

Esporte/atividade recreativa	Frequência e duração	Local	Características	Tipo de atividade
Exemplo: futebol/queimada/pega-pega	3 vezes por semana, 60 minutos	Clube do bairro	Grama, terreno irregular...	Em grupo, individual

▸ Ele (a) tem interesse em participar de algum esporte ou atividade recreativa? ☐ Sim ☐ Não Qual(is)? __________

▸ Observações: __________

QUESTIONÁRIO DO PERFIL DE ALFABETIZAÇÃO FÍSICA - QPAF

▸ PARTE II

▸ Lembre-se que nessa parte do questionário estamos avaliando o desempenho das habilidades e o grau de satisfação. Indique o nível de desempenho (de 0 a 2) e, na última coluna, marque com um X a satisfação com o desempenho dessa habilidade.

Físico					
**Habilidades**<br>No último mês, a criança, adolescente ou adulto jovem realizou atividades esportivas e recreativas:	**0**<br>não realiza	**1**<br>realiza parcialmente	**2**<br>realiza	**A** não sei<br>**B** não teve oportunidade	**Quão satisfeito você está com o desempenho da sua criança/adolescente/adulto jovem nessa habilidade?**
1. Usando habilidades de locomoção (ex.: correr, saltar, propulsão de cadeira de rodas ou qualquer equipamento que permita locomoção)?					1 2 3 4 5 6 7 8 9 10
2. Usando habilidades de manipulação de objetos utilizados na prática esportiva ou recreativa (ex.: arremessar, agarrar e quicar uma bola)?					1 2 3 4 5 6 7 8 9 10
3. Com coordenação (ex.: consegue realizar movimentos dos braços e pernas juntos, como quicar uma bola enquanto se locomove)?					1 2 3 4 5 6 7 8 9 10
4. Com força (ex.: usa o próprio corpo para se puxar, empurrar ou levantar do chão e/ou consegue levantar um objeto pesado, como uma bola pesada)?					1 2 3 4 5 6 7 8 9 10
5. Com agilidade e rapidez (ex.: se movimenta rapidamente para passar uma bola, alcança os colegas em brincadeiras de correr, etc.)?					1 2 3 4 5 6 7 8 9 10
6. Com resistência física (ex.:, consegue participar da brincadeira ou jogo sem ser interrompido pelo cansaço)?					1 2 3 4 5 6 7 8 9 10
7. Com equilíbrio (ex.: participa de brincadeiras ou jogos sem cair com frequência)?					1 2 3 4 5 6 7 8 9 10

▸ Observações: __________

QUESTIONÁRIO DO PERFIL DE ALFABETIZAÇÃO FÍSICA - QPAF

Social					
**Habilidades**<br>No último mês, a criança, adolescente ou adulto jovem realizou atividades esportivas e recreativas:	**0**<br>não realiza	**1**<br>realiza parcialmente	**2**<br>realiza	**A** não sei<br>**B** não teve oportunidade	**Quão satisfeito você está com o desempenho da sua criança/adolescente/adulto jovem nessa habilidade?**
8. Socializando com os colegas (ex.: interage de forma adequada com os companheiros de equipe)?					1 2 3 4 5 6 7 8 9 10
9. Socializando com adultos (ex.: interage de forma adequada com os profissionais, técnicos, outros pais)?					1 2 3 4 5 6 7 8 9 10
10. Colaborando com os demais (ex.: comunica, coopera e interage com seus colegas e treinador)?					1 2 3 4 5 6 7 8 9 10
11. Com empatia (ex.: demonstra justiça, inclusão e respeito com a equipe)?					1 2 3 4 5 6 7 8 9 10
12. Jogando bem em equipe (ex.: é um 'bom perdedor/ganhador', inclui os outros participantes, respeita a dinâmica da equipe)?					1 2 3 4 5 6 7 8 9 10
**Cognitivo**					
13. Conhecendo diferentes jogos e brincadeiras (ex.: queimada, futebol, etc.) e suas regras gerais (ex.: objetivo principal, forma de pontuação, etc.)?					1 2 3 4 5 6 7 8 9 10
14. Conhecendo estratégias (ex.: possíveis táticas esportivas e maneiras de jogar)?					1 2 3 4 5 6 7 8 9 10
15. Sabendo como se manter segura (ex.: usa capacete em esportes de risco, comporta-se com responsabilidade para não se machucar ou machucar o colega)?					1 2 3 4 5 6 7 8 9 10
16. Sabendo da sua importância (ex.: entende a importância de realizar uma atividade física e/ou sabe o que fazer para permanecer ativo)?					1 2 3 4 5 6 7 8 9 10
17. Reconhecendo e se ajustando a diferentes situações sem instruções (ex.: corre devagar em locais escorregadios ou é gentil com um jogador menor)?					1 2 3 4 5 6 7 8 9 10

QUESTIONÁRIO DO PERFIL DE ALFABETIZAÇÃO FÍSICA - QPAF

Psicológico					
**Habilidades**<br>No último mês, a criança, adolescente ou adulto jovem realizou atividades esportivas e recreativas:	**0**<br>não realiza	**1**<br>realiza parcialmente	**2**<br>realiza	**A** não sei<br>**B** não teve oportunidade	**Quão satisfeito você está com o desempenho da sua criança/adolescente/adulto jovem nessa habilidade?**
18. Com motivação (ex.:demonstrando empolgação para se envolver)?					1 2 3 4 5 6 7 8 9 10
19. Com engajamento (ex.:permanecendo envolvido na atividade)?					1 2 3 4 5 6 7 8 9 10
20. Com confiança (ex.:demonstrando segurança e acreditando que é possível realizar determinada atividade)?					1 2 3 4 5 6 7 8 9 10
21. Com autocontrole (ex.:controla a raiva ou angústia e supera o nervosismo sozinho)?					1 2 3 4 5 6 7 8 9 10
22. Sabendo reconhecer e gerenciar as necessidades físicas (ex.: quando precisa descansar, quando deve parar para tomar água, hora de ir ao banheiro)?					1 2 3 4 5 6 7 8 9 10
23. Conhecendo seus pontos fortes e desafios pessoais (ex.: sabe quais habilidades desempenha muito bem e em quais precisa melhorar)?					1 2 3 4 5 6 7 8 9 10
24. Explorando ambientes para praticar diferentes atividades (ex.: faz atividades diferentes em um ambiente favorito ou pratica uma atividade favorita em diferentes ambientes)?					1 2 3 4 5 6 7 8 9 10

▸ PONTUAÇÃO (Espaço reservado aos profissionais de saúde)

POR DOMÍNIO		GERAL
Domínio Físico<br>☐ (Soma dos itens 1-7) ÷ 14 = ☐ X 100 ☐ % (Pontuação total do domínio físico)	Domínio Cognitivo<br>☐ (Soma dos itens 13-17) ÷ 10 = ☐ X 100 = ☐ (Pontuação total do domínio cognitivo)	☐ (Soma dos itens 18-24) ÷ 48 = ☐ X 100 = ☐ (Pontuação geral do QPAF)
Domínio Social<br>☐ (Soma dos itens 8-12) ÷ 10 = ☐ X 100 = ☐ (Pontuação total do domínio social)	Domínio Psicológico<br>☐ (Soma dos itens 18-24) ÷ 14 = ☐ X 100 = ☐ (Pontuação total do domínio psicológico)	▸ Observações:

QUESTIONÁRIO DO PERFIL DE ALFABETIZAÇÃO FÍSICA - QPAF

ANEXO 6- CARTA DE ANUÊNCIA

CARTA DE ANUÊNCIA

Autorizo a utilização das Quadras Abertas de Voleibol e Basquete, de acordo a disponibilidade de agendamento, para a realização do Projeto de Pesquisa ***"Efetividade do Sports Stars Brasil sobre desfechos de atividade, participação e alfabetização física em crianças e adolescentes com transtorno do espectro do autismo: um ensaio controlado randomizado"*** a ser realizado sob orientação do Prof. Dr. Hércules Ribeiro Leite, do Programa de Pós-graduação em Ciências da Reabilitação dos Departamentos de Fisioterapia e Terapia Ocupacional da UFMG, em colaboração com a Profa. Dra. Andressa da Silva de Mello, do Departamentode Esportes.

Belo Horizonte, 26 de abril de 2022.

Profa. Kátia Lúcia Moreira Lemos

Assinado de forma digital por Profa. Kátia Lúcia Moreira Lemos
DN: cn=Profa. Kátia Lúcia Moreira Lemos, o, ou=Universidade Federal de Minas Gerais, email=[illegible], c=BR
Dados: 2022.04.28 09:10:15 -03'00'

APÊNDICE A - TERMO DE CONSENTIMENTO LIVRE E ESCLARECIDO

TERMO DE CONSENTIMENTO LIVRE E ESCLARECIDO
(PAIS, MÃES OU RESPONSÁVEIS)

Título do Estudo: **Efetividade do *Sports Stars* Brasil sobre desfechos de atividade, participação e alfabetização física em adolescentes com transtorno do espectro do autismo: um ensaio controlado randomizado**

Prezados pais ou responsáveis,

O Sr(a) e seu filho estão convidados a participar desta pesquisa coordenada pelo professor Dr. Hércules Ribeiro Leite que tem como objetivo investigar a efetividade do *Sports Stars Brasil,* um tratamento fisioterapêutico em grupo, centrado na realização de atividades esportivas em crianças e adolescentes com Transtorno do Espectro Autista (TEA)*;* e investigar se essa intervenção combinada com um acompanhamento fisioterápico no contexto do esporte é mais eficaz para facilitar a participação dos indivíduos em atividades esportivas. Para realizar essa pesquisa, nós precisamos de sua autorização para que seu (sua) filho(a) possa participar do estudo. Sua participação nesse estudo nos ajudará a investigar se o *Sports Stars* é um tratamento eficaz para introduzir crianças e adolescentes com TEA em atividades esportivas.

Após a obtenção do seu consentimento para participar desta intervenção, haverá um sorteio para verificar se seu filho (a) participará do programa *Sports Stars* imediatamente ou depois de quatro meses de tratamento fisioterápico que ele realiza normalmente. O programa *Sports Stars* ocorrerá em grupos de 3-4 participantes com idades semelhantes, uma vez por semana, uma hora cada, durante oito semanas consecutivas, nas quadras esportivas da Escola de Educação Física, Fisioterapia e Terapia Ocupacional da Universidade Federal de Minas Gerais. Durante cada sessão semanal serão trabalhadas habilidades motoras de seu (sua) filho(a), através de atividades de corrida, salto, atividades com bola, assim como a introdução a prática dos esportes: futebol, handball, atletismo e basquete. Caso seu(sua) filho(a) seja sorteado para realizar o programa após quatro meses, ele(a) continuará realizando as atividades de fisioterapia que ele já costuma realizar, durante este tempo, conforme for estabelecido pelo profissional que atende seu (sua) filho (a).

Após seu (sua) filho(a) realizar o programa *Sports Stars* seu(sua) filho(a) será encaminhado(a) para realizar atividades esportivas, uma vez por semana, em torno de uma hora, em algum programa e modalidade de esporte de sua preferência.

Após o seu consentimento, realizaremos uma avaliação fisioterápica com você e seu(sua) filho (a). Você será perguntado(a) através de uma série de questionários sobre as características do seu filho(a); sobre como o seu filho participa em atividades do dia-a-dia (em casa, na escola e na comunidade); sobre quais as principais dificuldades que ele(a) tem para realizar atividades de corrida, salto e atividades com bola; e sobre o que você gostaria que seu(sua) filho(a) fizesse diferente para ser capaz de realizar atividades físicas e recreativas. Estes questionários levarão em torno de 30 minutos para serem respondidos. Seu (sua) filho(a) realizará uma avaliação física guiada por um fisioterapeuta treinado. Nessa avaliação faremos testes que avaliarão a força muscular, equilíbrio e como seu(sua) filho(a) realiza atividades de corrida, salto e atividades com bola. Essa série de avaliações tem o objetivo de verificar o que seu(sua) filho(a) é capaz de fazer. As avalições do(a) seu(sua) filho(a) durarão em torno de uma hora com períodos de descanso, caso seja necessário. Caso você não queira que seu(sua) filho(a) realize alguma das atividades propostas, o teste será interrompido em qualquer momento. Essas avaliações e questionários serão repetidos após repetidos após 7 dias, 8 semanas e 12 semanas da primeira avaliação em algum horário que você e seu (sua) filho(a) estejam disponíveis.

Por se tratar de um treino de atividades dinâmicas e esportivas em grupo, esta intervenção, bem como o processo de a avaliação, oferecem um pequeno risco de que seu(sua) filho(a) se canse, caia ou se machuque durante as atividades esportivas. Assim, faremos todas as atividades e exercícios e todas avaliações com a supervisão máxima de um profissional treinado. Caso ocorra alguma intercorrência as atividades serão interrompidas e nossa equipe dará completa assistência a você e ao seu (sua) filho(a) e os devidos primeiros

socorros caso necessário. Todas as avaliações realizadas com seu(sua) filho(a) serão filmadas para pontuação dos testes. Você ou seu(sua) filho (a) poderão se sentir constrangidos durante a filmagem. Para evitar que isso aconteça, todos os detalhes dos procedimentos para sigilo das filmagens serão previamente explicados e discutidos com você e seu (sua) filho(a). Ressaltamos que os vídeos serão utilizados apenas para pontuar o teste. Os vídeos obtidos pelas filmagens serão mantidos em completo sigilo. Ao responder os questionários, durante a realização dos testes e das atividades do programa, você e/ou seu(sua) filho(a) poderão se sentir desconfortáveis ou constrangidos com alguma pergunta ou procedimento. Caso isso aconteça poderemos interromper qualquer um dos testes ou questionários bem como as atividades do programa, em qualquer momento, e será respeitada a sua vontade sem nenhum prejuízo para vocês.

Para garantir que as informações desse estudo sejam confidenciais, as informações obtidas de você e de sua criança receberão um código de identificação ao entrar no estudo e o nome do seu(ua) filho(a) não será divulgado em qualquer situação. Os dados e vídeos gerados nesta pesquisa serão armazenados na Escola de Educação Física, Fisioterapia e Terapia Ocupacional da UFMG por 5 anos no gabinete 3125 do professor Dr. Hércules Ribeiro Leite (telefones de contato no final deste documento) e ficará sob responsabilidade, do mesmo. Se as informações originadas do estudo forem publicadas em revista ou evento científico, você e sua criança não serão identificados, sendo sempre representados por abreviações ou nomes fictícios.

Para essa pesquisa são esperados alguns benefícios, dentre eles: as informações obtidas do estudo poderão contribuir para a explicar os possíveis benefícios de um tratamento fisioterapêutico em grupo, centrado na realização de atividades esportivas em crianças e adolescentes com Transtorno do Espectro Autista e as estratégias educativas que serão oferecidas poderão favorecer o entendimento a respeito das limitações funcionais das crianças e doas adolescentes participantes, favorecendo os cuidados de saúde e promovendo a funcionalidade, a participação social e a alfabetização física das crianças e adolescentes com TEA, em diferentes contextos.

Ressaltamos que sua participação nesta pesquisa é inteiramente voluntária e vocês não receberão nenhum pagamento ou compensação financeira para participar. Além disso, vocês não terão nenhum tipo de despesa adicional com este estudo. Caso você tenha gastos com deslocamento para as avaliações ou para realizar as intervenções do programa, nossa equipe ficará responsável em te reembolsar com o valor da condução em dinheiro (ida e volta) em cada encontro ou momento de avaliação. É importante destacar também que você e seu(sua) filho(a) são livres para consentir na participação ou no abandono do estudo a qualquer momento. Haverá duas vias deste documento e uma via o é destinada a você em caso de dúvidas. Você poderá obter qualquer informação deste estudo com os pesquisadores, e informações de aspecto ético no Comitê de Ética em Pesquisa da Universidade Federal de Minas Gerais (UFMG). Os telefones estão listados abaixo. Estaremos a sua disposição para responder perguntas ou prestar esclarecimentos sobre o andamento do trabalho.

Caso você concorde em participar do estudo, por favor, assine no espaço indicado abaixo.

Agradecemos a sua colaboração.

Atenciosamente,

____

Prof. Hércules Ribeiro Leite
Coordenador da Pesquisa
Professor Adjunto-Departamento de Fisioterapia, UFMG

____

Amanda Cristina Fernandes
Fisioterapeuta

CONSENTIMENTO

Eu,____, responsável por _____________________________________declaro que li e entendi todas as informações sobre o "**Efetividade do *Sports Stars* Brasil sobre desfechos de atividade, participação e alfabetização física em crianças e adolescentes com transtorno do espectro do autismo: um ensaio controlado randomizado",** sendo os objetivos e procedimentos explicados claramente. Tive tempo suficiente para pensar e escolher participar do estudo e tive oportunidade de tirar todas as minhas dúvidas. Estou assinando este termo voluntariamente e tenho direito de, agora ou mais tarde, discutir qualquer dúvida em relação ao projeto.

____

Assinatura do pai/responsável

Belo Horizonte,_____de________________de 20___.

Telefone para contato/informações:

Professor Dr. Hércules Ribeiro Leite
Departamento de Fisioterapia, UFMG
Fone: (31) 3409-7404 email: herculesdtnaa@gmail.com

Amanda Cristina Fernandes
Fisioterapeuta
Fone: (38) 99912-7785 email: amandacristina40@gmail.com

Em caso de dúvidas relacionadas às questões éticas:

Comitê de Ética em Pesquisa - COEP/UFMG: Av. Pres. Antônio Carlos, 6627 –Unidade Administrativa II 2º. Andar –Sala 2005 – CEP 31270-901 Belo Horizonte – MG Telefone: (31) 3409-4592. E-mail: coep@prpq@ufmg.br

APÊNDICE B – TERMO DE CONSENTIMENTO LIVRE E ESCLARECIDO

TERMO DE CONSENTIMENTO LIVRE E ESCLARECIDO

(PAIS, MÃES OU RESPONSÁVEIS)

Título do Estudo: **Viabilidade e efeito do Sports Stars Brasil em adolescentes com transtorno do espectro autista**

Prezados pais ou responsáveis,

O Sr(a) e seu filho estão convidados a participar desta pesquisa coordenada pelo professor Dr. Hércules Ribeiro Leite que tem como objetivo investigar a efetividade do *Sports Stars Brasil,* um tratamento fisioterapêutico em grupo, centrado na realização de atividades esportivas em adolescentes com Transtorno do Espectro Autista (TEA)*;* e investigar se essa intervenção combinada com um acompanhamento fisioterápico no contexto do esporte é mais eficaz para facilitar a participação dos indivíduos em atividades esportivas. Para realizar essa pesquisa, nós precisamos de sua autorização para que seu (sua) filho(a) possa participar do estudo. Sua participação nesse estudo nos ajudará a investigar se o *Sports Stars* é um tratamento eficaz para introduzir adolescentes com TEA em atividades esportivas.

O programa *Sports Stars* ocorrerá em grupos de 4 participantes com idades semelhantes, uma vez por semana, uma hora cada, durante oito semanas consecutivas, nas quadras esportivas da Escola de Educação Física, Fisioterapia e Terapia Ocupacional da Universidade Federal de Minas Gerais. Durante cada sessão semanal serão trabalhadas habilidades motoras de seu (sua) filho(a), através de atividades de corrida, salto, atividades com bola, assim como a introdução a prática dos esportes: futebol, handball, atletismo e basquete.

Após seu (sua) filho(a) realizar o programa *Sports Stars* seu(sua) filho(a) será encaminhado(a) para realizar atividades esportivas, uma vez por semana, em torno de uma hora, em algum programa e modalidade de esporte de sua preferência.

Após o seu consentimento, realizaremos uma avaliação fisioterápica com você e seu(sua) filho (a). Você será perguntado(a) através de uma série de questionários sobre as características do seu filho(a); sobre como o seu filho participa em atividades do dia-a-dia (em casa, na escola e na comunidade); sobre quais as principais dificuldades que ele(a) tem para realizar atividades de corrida, salto e atividades com bola; e sobre o que você gostaria que seu(sua) filho(a) fizesse diferente para ser capaz de realizar atividades físicas e recreativas. Estes questionários levarão em torno de 30 minutos para serem respondidos. Seu (sua) filho(a) realizará uma avaliação física guiada por um fisioterapeuta treinado. Nessa avaliação faremos testes que avaliarão a força muscular, equilíbrio e como seu(sua) filho(a) realiza atividades de corrida, salto e atividades com bola. Essa série de avaliações tem o objetivo de verificar o que seu(sua) filho(a) é capaz de fazer. As avaliações do(a) seu(sua) filho(a) durarão em torno de uma hora com períodos de descanso, caso seja necessário. Caso você não queira que seu(sua) filho(a) realize alguma das atividades propostas, o teste será interrompido em qualquer momento. Essas avaliações e questionários serão repetidos após 8 e 12 semanas da primeira avaliação em algum horário que você e seu (sua) filho(a) estejam disponíveis.

Por se tratar de um treino de atividades dinâmicas e esportivas em grupo, esta intervenção, bem como o processo de avaliação, oferecem um pequeno risco de que seu(sua) filho(a) se canse, caia ou se machuque durante as atividades esportivas. Assim, faremos todas as atividades e exercícios e todas avaliações com a supervisão máxima de um profissional treinado. Caso ocorra alguma intercorrência as atividades serão interrompidas e nossa equipe dará completa assistência a você e ao seu (sua) filho(a) e os devidos primeiros socorros caso necessário. Todas as avaliações realizadas com seu(sua) filho(a) serão filmadas para pontuação dos testes. Você ou seu(sua) filho (a) poderão se sentir constrangidos durante a filmagem. Para evitar que isso aconteça, todos os detalhes dos procedimentos para sigilo das filmagens serão previamente explicados e discutidos com você e seu (sua) filho(a). Ressaltamos que os vídeos serão utilizados apenas para pontuar o teste. Os vídeos obtidos pelas filmagens serão mantidos

em completo sigilo. Ao responder os questionários, durante a realização dos testes e das atividades do programa, você e/ou seu(sua) filho(a) poderão se sentir desconfortáveis ou constrangidos com alguma pergunta ou procedimento. Caso isso aconteça poderemos interromper qualquer um dos testes ou questionários bem como as atividades do programa, em qualquer momento, e será respeitada a sua vontade sem nenhum prejuízo para vocês.

Para garantir que as informações desse estudo sejam confidenciais, as informações obtidas de você e de sua criança receberão um código de identificação ao entrar no estudo e o nome do seu(ua) filho(a) não será divulgado em qualquer situação. Os dados e vídeos gerados nesta pesquisa serão armazenados na Escola de Educação Física, Fisioterapia e Terapia Ocupacional da UFMG por 5 anos no gabinete 3125 do professor Dr. Hércules Ribeiro Leite (telefones de contato no final deste documento) e ficará sob responsabilidade, do mesmo. Se as informações originadas do estudo forem publicadas em revista ou evento científico, você e sua criança não serão identificados, sendo sempre representados por abreviações ou nomes fictícios.

Para essa pesquisa são esperados alguns benefícios, dentre eles: as informações obtidas do estudo poderão contribuir para a explicar os possíveis benefícios de um tratamento fisioterapêutico em grupo, centrado na realização de atividades esportivas em crianças e adolescentes com Transtorno do Espectro Autista e as estratégias educativas que serão oferecidas poderão favorecer o entendimento a respeito das limitações funcionais dos adolescentes participantes, favorecendo os cuidados de saúde e promovendo a funcionalidade, a participação social e a alfabetização física dos adolescentes com TEA, em diferentes contextos.

Ressaltamos que sua participação nesta pesquisa é inteiramente voluntária e vocês não receberão nenhum pagamento ou compensação financeira para participar. Além disso, vocês não terão nenhum tipo de despesa adicional com este estudo. Caso você tenha gastos com deslocamento para as avaliações ou para realizar as intervenções do programa, nossa equipe ficará responsável em te reembolsar com o valor da condução em dinheiro (ida e volta) em cada encontro ou momento de avaliação. É importante destacar também que você e seu(sua) filho(a) são livres para consentir na participação ou no abandono do estudo a qualquer momento. Haverá duas vias deste documento é uma via que é destinada a você em caso de dúvidas. Você poderá obter qualquer informação deste estudo com os pesquisadores, e informações de aspecto ético no Comitê de Ética em Pesquisa da Universidade Federal de Minas Gerais (UFMG). Os telefones estão listados abaixo. Estaremos a sua disposição para responder perguntas ou prestar esclarecimentos sobre o andamento do trabalho.

Caso você concorde em participar do estudo, por favor, assine no espaço indicado abaixo.

Agradecemos a sua colaboração.

Atenciosamente,

____

Prof. Hércules Ribeiro Leite

Coordenador da Pesquisa

Professor Adjunto-Departamento de Fisioterapia, UFMG

____

Lidiane Francisca Borges

Fisioterapeuta

____

Amanda Cristina Fernandes

Fisioterapeuta

CONSENTIMENTO

Eu, ____, responsável por _____________________________________declaro que li e entendi todas as informações sobre o "**Viabilidade e efeito do Sports Stars em adolescentes com transtorno do espectro autista",** sendo os objetivos e procedimentos explicados claramente. Tive tempo suficiente para pensar e escolher participar do estudo e tive oportunidade de tirar todas as minhas dúvidas. Estou assinando este termo voluntariamente e tenho direito de, agora ou mais tarde, discutir qualquer dúvida em relação ao projeto.

____

Assinatura do pai/responsável

Belo Horizonte,_____de________________de 20___.

Telefone para contato/informações:

Professor Dr. Hércules Ribeiro Leite

Departamento de Fisioterapia, UFMG

Telefone: (31) 3409-7404 - E-mail: herculesdtna@gmail.com

Lidiane Francisca Borges Ferreira

Fisioterapeuta

Telefone: (31) 98824-8035 - E-mail: lidiborges.fisio@gmail.com

Amanda Cristina Fernandes

Fisioterapeuta

Telefone: (38) 99912-7785- E-mail: amandacristina40@gmail.com

Em caso de dúvidas relacionadas às questões éticas:

Comitê de Ética em Pesquisa - COEP/UFMG: Av. Pres. Antônio Carlos, 6627 –Unidade Administrativa II 2º. Andar –Sala 2005 – CEP 31270-901 Belo Horizonte – MG Telefone: (31) 3409-4592 - E-mail: coep@prpq@ufmg.br

APÊNDICE C – TERMO DE ASSENTIMENTO LIVRE E ESCLARECIDO

TERMO DE ASSENTIMENTO LIVRE E ESCLARECIDO

(CRIANÇAS 6 ANOS A 11 ANOS E 29 DIAS)

Título da Pesquisa: Efetividade do Sports Stars Brasil sobre a participação e alfabetização física de crianças e adolescentes com Transtorno do Espectro do Autismo: Um ensaio controlado randomizado.

Olá,

Você está convidado a participar desta pesquisa coordenada pelo professor Dr. Hércules Ribeiro Leite que vai estudar sobre um tratamento de Fisioterapia em grupo chamado *Sports Stars*. Este tratamento foca na realização de atividades de esportes para crianças e adolescentes com Transtorno do Espectro Autista (TEA). Nossa pesquisa também quer descobrir se o programa *Sports Stars* também funciona quando é combinada com um acompanhamento de um fisioterapeuta durante a realização de esportes. Para realizar essa pesquisa, nós precisamos de sua autorização. Sua participação nos ajudará a entender se o *Sports Stars* é um bom tratamento para incentivar crianças e adolescentes com TEA a realizarem esportes. Os detalhes dessa pesquisa estão explicados a seguir:

UMA AVENTURA COM O SPORT STARS BRASIL

OI PESSOAL, MEU NOME É ANDRÉ E TENHO AUTISMO, VAMOS CONVERSAR SOBRE O SPORT STARS BRASIL? O PROJETO É UMA FISIOTERAPIA EM GRUPO, COM ATÉ 6 CRIANÇAS COMO A GENTE.	BEM LEMBRADO ANDRÉ E O MAIS LEGAL É QUE ELE VAI ACONTECER 1 VEZ NA SEMANA, POR 1 HORA, DURANTE 2 MESES. SERÁ NAS QUADRAS DA UFMG. E QUEREM SABER QUAIS SERÃO AS NOSSAS BRINCADEIRAS?	VAMOS BRINCAR DE CORRIDA, SALTO, HANDBALL, FUTEBOL, BASQUETE E ATLETISMO.
AGORA VAMOS ENTENDER COMO SERÁ A DINÂMICA. PARA PARTICIPAR DO PROJETO, VOCÊ E O PAPAI OU MAMÃE, TERÃO QUE CONCORDAR E ASSINAR.	HAVERÁ UM SORTEIO PARA DEFINIR SE VOCÊ VAI PARTICIPAR AGORA OU DAQUI 4 MESES. SE VOCÊ FOR SORTEADO PARA PARTCIPAR DAQUI 4 MESES, VOCÊ VAI CONTINUAR REALIZANDO AS ATIVIDADES DO SEU DIA A DIA.	O PAPAI OU A MAMÃE OU SEU RESPONSÁVEL, RESPONDERÃO PERGUNTAS SOBRE SUAS CARACTERÍSTICAS, COMO É SUA PARTICIPAÇÃO EM ATIVIDADES DO DIA A DIA E QUAIS SÃO SUAS DIFICULDADES PARA REALIZAR ATIVIDADES DE CORRIDA, SALTO E ATIVIDADES COM BOLA. LEVARÁ CERCA DE 30 MINUTINHOS. E PRECISAREMOS DURANTE ESSAS AVALIAÇÕES, REALIZAR FILMAGENS, GRAVAR AUDIOS E TIRAR FOTOS, PARA DEPOIS SABERMOS EM QUAL ATIVIDADE VOCÊ CONSEGUIU GANHAR MAIS PONTOS.

BOM, NOSSAS BRINCADEIRAS DURAM CERCA DE 60 MINUTOS. PORTANTO, CASO QUEIRE, VOCÊ PODE DESCANSAR ENTRE ELAS.	COMO VOCÊ JÁ SABE, IREMOS BRINCAR DE CORRER, PULAR, CHUTAR E OUTRAS BRINCADEIRAS. PODE ACONTECER DE VOCÊ SE CANSAR, CAIR OU MACHUCAR.	MAS NÃO SE PREOCUPE, TODAS AS SUAS ATIVIDADES E AVALIAÇÕES, SERÃO REALIZADAS COM UM FISIOTERAPEUTA PERTINHO DE VOCÊ, PARA QUE EVITE QUE ISSO OCORRA.
MAS SE OCORRER, TEMOS UMA EQUIPE DE SUPER HERÓIS PRONTOS PARA TE AJUDAR EM TUDO O QUE FOR NECESSÁRIO	UM DETALHE MUITO IMPORTANTE TODOS DEVEM FAZER USO DA MÁSCARA PARA SE PROTEGEREM DO CORONA VÍRUS	GOSTOU? ENTÃO VENHA PARTICIPAR COM A GENTE. E CASO VOCÊ SEJA SORTEADO PARA BRINCAR DAQUI ALGUNS MESES, NÃO SE PREOCUPE, O TEMPO PASSA RAPIDINHO E LOGO ESTAREMOS JUNTOS.

Fonte: próprio autor

Para essa pesquisa são esperados alguns benefícios, dentre eles: as informações obtidas do estudo poderão contribuir para a explicar os possíveis benefícios de um tratamento fisioterapêutico em grupo, centrado na realização de atividades esportivas em crianças e adolescentes com Transtorno do Espectro Autista e as estratégias educativas que serão oferecidas poderão favorecer o entendimento a respeito das limitações funcionais das crianças e doas adolescentes participantes, favorecendo os cuidados de saúde e promovendo a funcionalidade, a participação social e a alfabetização física das crianças e adolescentes com autismo, em diferentes contextos.

Para garantir que as informações desse estudo sejam guardadas e que ninguém possa ter acesso, as informações obtidas de você receberão um código e o seu nome não será divulgado em qualquer situação. Os dados e vídeos gerados nesta pesquisa serão armazenados na Escola de Educação Física, Fisioterapia e Terapia Ocupacional da UFMG por 5 anos no gabinete 3125 do professor Dr. Hércules Ribeiro Leite (telefones de contato no final deste documento) e ficará sob responsabilidade, do mesmo. Se as informações deste

estudo forem publicadas em revista ou congresso, você não será identificado, sendo sempre utilizado códigos ou nomes de mentira.

A sua participação nesta pesquisa é voluntária e seus pais ou responsáveis não receberão nenhum pagamento para participar. Além disso, vocês não terão nenhum tipo de gasto com este estudo. Caso seus pais tenham gastos com o transporte para as avaliações ou para realizar as sessões do programa, nossa equipe ficará responsável devolver o valor do dinheiro da passagem (ida e volta) em cada encontro ou momento de avaliação. É importante destacar também que você é livre para participar ou abandona o estudo a qualquer momento. Uma via deste documento é para a você em caso de dúvidas. Você poderá ter qualquer informação deste estudo com os pesquisadores e com o Comitê de Ética em Pesquisa da Universidade Federal de Minas Gerais (UFMG). Os telefones estão abaixo. Se você precisar pode fazer perguntas sobre o andamento do trabalho.

Caso você concorde em participar do estudo, por favor, assine no espaço indicado abaixo.

Agradecemos a sua colaboração.

Atenciosamente,

Amanda Cristina Fernandes
Fisioterapeuta

Prof. Hércules Ribeiro Leite
Coordenador da Pesquisa
Professor Adjunto-Departamento de Fisioterapia, UFMG

Consentimento

Eu,____, responsável por ____________________________________declaro que li e entendi todas as informações sobre o "**Efetividade do *Sports Stars* Brasil sobre desfechos de atividade, participação e alfabetização física em crianças e adolescentes com transtorno do espectro do autismo: um ensaio controlado randomizado",** sendo os objetivos e procedimentos explicados claramente. Tive tempo suficiente para pensar e escolher participar do estudo e tive oportunidade de tirar todas as minhas dúvidas. Estou assinando este termo voluntariamente e tenho direito de, agora ou mais tarde, discutir qualquer dúvida em relação ao projeto.

____

Assinatura ou iniciais da criança

Belo Horizonte,_____de________________de 20___.

Telefone para contato/informações:
Professor Dr. Hércules Ribeiro Leite
Departamento de Fisioterapia, UFMG
Fone: (31) 3409-7404 email: herculesdtnaa@gmail.com

Amanda Cristina Fernandes
Fisioterapeuta
Fone: (38) 99912-7785 email: amandacristina40@gmail.com

Em caso de dúvidas relacionadas às questões éticas:
Comitê de Ética em Pesquisa - COEP/UFMG: Av. Pres. Antônio Carlos, 6627 –Unidade Administrativa II 2º. Andar –Sala 2005 – CEP 31270-901 Belo Horizonte – MG Telefone: (31) 3409-4592. E-mail: coep@prpq@ufmg.br

APÊNDICE D – TERMO DE ASSENTIMENTO LIVRE E ESCLARECIDO

TERMO DE ASSENTIMENTO LIVRE E ESCLARECIDO

(ADOLESCENTES 12 A 17 ANOS E 11 MESES)

Título do Estudo: **Viabilidade e efeito do Sports Stars Brasil em adolescentes com transtorno do espectro autista**

Prezado adolescente,

Você está convidado a participar desta pesquisa coordenada pelo professor Dr. Hércules Ribeiro Leite que tem como objetivo investigar os resultados de um tratamento de Fisioterapia em grupo chamado *Sports Stars*. Este tratamento foca na realização de atividades esportivas para adolescentes com Transtorno do Espectro Autista (TEA). Nossa pesquisa também quer descobrir se essa intervenção também funciona quando é combinada com um acompanhamento de um fisioterapeuta durante a prática esportiva de adolescentes. Para realizar essa pesquisa, nós precisamos de sua autorização. Sua participação nessa pesquisa nos ajudará a investigar se o *Sports Stars* é um tratamento eficaz para incentivar adolescentes com TEA a realizarem atividades esportivas.

O programa *Sports Stars* vai acontecer em grupos de 4-6 participantes com idades parecidas (um grupo somente com adolescentes), uma vez por semana, uma hora cada, durante oito semanas nas quadras esportivas da Escola de Educação Física, Fisioterapia e Terapia Ocupacional da Universidade Federal de Minas Gerais. Durante cada atendimento serão trabalhadas com você habilidades de corrida, salto, atividades com bola e também vamos te ensinar a praticar alguns esportes: futebol, handball, atletismo e basquete.

Após você e seus pais/responsáveis aceitarem participar, nós realizaremos uma avaliação com fisioterapeuta com você e seus pais. Seus pais ou responsáveis serão perguntados(as) através de uma entrevista sobre as suas características; sobre como você participa em atividades do dia-a-dia (em casa, na escola e no seu bairro); sobre quais as principais dificuldades você tem para realizar atividades de corrida, salto e atividades com bola; e sobre o que eles gostariam que você fizesse diferente para ser capaz de realizar atividades físicas e de lazer. Estes questionários levarão em torno de 30 minutos para serem respondidos. Você realizará alguns testes com um fisioterapeuta treinado. Nessa avaliação vamos observar como está a sua força muscular e como você realiza atividades de corrida, salto e atividades com bola. Esses testes são para nós observarmos o que você é capaz de fazer. Essas avaliações durarão em torno de uma hora com períodos de descanso, caso seja você precise. Caso você não queira que realizar alguma das atividades, nós vamos parar o teste em qualquer momento. Essas avaliações e entrevistas serão repetidas depois de 8 e 12 semanas da primeira avaliação em algum horário que você esteja disponível.

Como faremos atividades agitadas e esportivas em grupo, este tratamento e os testes podem levar a um pequeno risco de você se cansar, cair ou se machucar durante as atividades esportivas. Assim, faremos todas as atividades e exercícios e todas avaliações com um fisioterapeuta bem próximo de você. Caso aconteça alguma coisa, nós vamos parar as atividades e nossa equipe vai cuidar de você e ficaremos responsáveis por qualquer dano ou risco. Todas as avaliações realizadas por você serão filmadas para pontuação dos testes. Você ou seus pais podem se sentir com vergonha durante a filmagem. Para evitar que isso aconteça, todos os detalhes do que vamos fazer com a filmagem vão ser explicados antes. Os vídeos serão utilizados somente para observar os seus testes com atenção. Os vídeos obtidos pelas filmagens serão mantidos guardados. Ao responder os questionários, durante a realização dos testes e das atividades do programa, você e/ou seus pais poderão se sentir constrangidos com alguma pergunta ou procedimento. Caso isso aconteça poderemos para qualquer um dos testes ou questionários bem como

as atividades do programa, em qualquer momento, e será respeitada a sua vontade sem nenhum problema.

Para essa pesquisa são esperados alguns benefícios, dentre eles: as informações obtidas do estudo poderão contribuir para a explicar os possíveis benefícios de um tratamento fisioterapêutico em grupo, centrado na realização de atividades esportivas em crianças e adolescentes com Transtorno do Espectro Autista e as estratégias educativas que serão oferecidas poderão favorecer o entendimento a respeito das limitações funcionais das crianças e doas adolescentes participantes, favorecendo os cuidados de saúde e promovendo a funcionalidade, a participação social e a alfabetização física das crianças e adolescentes com autismo, em diferentes contextos.

Para garantir que as informações desse estudo sejam guardadas e que ninguém possa ter acesso, as informações obtidas de você receberão um código e o seu nome não será divulgado em qualquer situação. Os dados e vídeos gerados nesta pesquisa serão armazenados na Escola de Educação Física, Fisioterapia e Terapia Ocupacional da UFMG por 5 anos no gabinete 3125 do professor Dr. Hércules Ribeiro Leite (telefones de contato no final deste documento) e ficará sob responsabilidade, do mesmo. Se as informações originadas do estudo forem publicadas em revista ou evento científico, você e sua criança não serão identificados, sendo sempre representados por abreviações ou nomes fictícios.

A sua participação nesta pesquisa é inteiramente voluntária e vocês não receberão nenhum pagamento para participar. Além disso, vocês não terão nenhum tipo de gasto com este estudo. Caso seus pais tenham gastos com o transporte para as avaliações ou para realizar as sessões do programa, nossa equipe ficará responsável devolver o valor da condução em dinheiro (ida e volta) em cada encontro ou momento de avaliação. É importante destacar também que você é livre para participar ou abandonar o estudo a qualquer momento. Uma via deste documento é para a você em caso de dúvidas. Você poderá obter qualquer informação deste estudo com os pesquisadores e com o Comitê de Ética em Pesquisa da Universidade Federal de Minas Gerais (UFMG). Os telefones estão listados abaixo. Estaremos a sua disposição para responder perguntas ou prestar esclarecimentos sobre o andamento do trabalho.

Caso você concorde em participar do estudo, por favor, assine no espaço indicado abaixo.

Agradecemos a sua colaboração.

Atenciosamente,

____

Prof. Hércules Ribeiro Leite

Coordenador da Pesquisa

Professor Adjunto-Departamento de Fisioterapia, UFMG

____

Lidiane Francisca Borges Ferreira

Fisioterapeuta

____

Amanda Cristina Fernandes

Fisioterapeuta

Consentimento

Eu,____declaro que li e entendi todas as informações sobre o "**Viabilidade e efeito do Sports Stars em adolescentes com transtorno do espectro autista",** sendo os objetivos e procedimentos explicados de forma clara. Tive tempo suficiente para pensar e escolher participar do estudo e tive oportunidade de tirar todas as minhas dúvidas. Estou assinando este termo voluntariamente e tenho direito de, agora ou mais tarde, discutir qualquer dúvida em relação ao projeto.

____

Assinatura do adolescente

Belo Horizonte,_____de________________de 20___.

Telefone para contato/informações:

Professor Dr. Hércules Ribeiro Leite

Departamento de Fisioterapia, UFMG

Telefone: (31) 3409-7404 - E-mail: herculesdtna@gmail.com

Lidiane Francisca Borges Ferreira

Fisioterapeuta

Telefone: (31) 98824-8035 - E-mail: lidiborges.fisio@gmail.com

Amanda Cristina Fernandes

Fisioterapeuta

Telefone: (38) 99912-7785- E-mail: amandacristina40@gmail.com

Laboratório de Investigação & Intervenção no Desenvolvimento na Infância e Adolescência (IDEIA)

Telefone: (31) 3409-4796

Em caso de dúvidas relacionadas às questões éticas:

Comitê de Ética em Pesquisa - COEP/UFMG: Av. Pres. Antônio Carlos, 6627 –Unidade Administrativa II 2º. Andar –Sala 2005 – CEP 31270-901 Belo Horizonte – MG Telefone: (31) 3409-4592 - E-mail: coep@prpq@ufmg.br
