# Supplementary material for: Sports Stars Brazil in children with autism spectrum disorder: A feasibility randomized controlled trial protocol
Source: PLoS One. 2023 Nov 8;18(11):e0291488. doi: 10.1371/journal.pone.0291488 (PMC10631688; doi:10.1371/journal.pone.0291488)
Supplement: Attachment — Submitted filename: Comments to authors.docx [file pone.0291488.s008.docx]

**Comments to authors**

The paper aims to assess Effectiveness of Sports Stars Brazil in children with autism spectrum disorder: A randomized controlled trial protocol Sports Stars Brazil in children with autism spectrum disorder

The paper is relevant in its discipline. Although the paper is well written, the authors need to consider some issues as highlighted below.

**Title**

**Should you use the word “effectiveness” or “effect”?**

**Abstract**
your abstract is well written but it would be better to include the **Background** which should include:

- **Case (context)**
- **Gap (Punch Line)**

**Background**

This section should give a summary of the main issue of the study. It is better to include the magnitude of the problem from globe to local, efforts made by the government or other concerned body to address the issue and the gap to be filled by this study.

**Methods**

Line 1 “an is” should be removed.

Line 2 wil should be changed to will.

The sampling procedure – in your document “procedure” is not clearly written. As this is a randomized controlled trial, how are going to select the participants and randomize??

Is it individual randomization? How are you going to minimize information contamination as this is a kind of behavior intervention??

These issues should be clearly stated in the sampling procedure?

Your sample size calculation is also not clear. Even if there is no previous study to estimate the effect size, you could have used the conventional effect size of small, medium or high and calculated your sample size.

Data analysis

“Differences between groups will be calculated using unpaired t tests”. Better to say independent t-test. There is not unpaired t- test in statistics. Paired - t-test or independent t- test.

“Differences between groups over time will be analyzed using repeated measured analysis of variance followed by Tukey post hoc test for parametric data”.

ANOVA is used for the crude analysis of mean difference between groups. It does not show the difference in difference. So it is advisable to use appropriate statistical models to determine the difference overtime between the groups.

**Ethical consideration**
“All participants signed the informed consent form”. But your study population is under the legal age to give informed consent.

From whom are you going to obtain consent? Assent??
