# Supplementary material for: Sports Stars Brazil in children with autism spectrum disorder: A feasibility randomized controlled trial protocol
Source: PLoS One. 2023 Nov 8;18(11):e0291488. doi: 10.1371/journal.pone.0291488 (PMC10631688; doi:10.1371/journal.pone.0291488)
Supplement: Attachment — Submitted filename: renamed_ff0a7.docx [file pone.0291488.s009.docx]

# COMMENTS – Manuscript PONE-D-22-31855

Title: “Effectiveness of Sports Stars Brazil in children with autism spectrum disorder: A randomized controlled trial protocol Sports Stars Brazil in children with autism spectrum disorder”

**Important note: This review pertains only to ‘statistical aspects’ of the study and so ‘clinical aspects’ [like medical importance, relevance of the study, ‘clinical significance and implication(s)’ of the whole study, etc.] are to be evaluated [should be assessed] separately/independently. Further please note that any ‘statistical review’ is generally done under the assumption that (such) study specific methodological [as well as execution] issues are perfectly taken care of by the investigator(s). This review is not an exception to that and so does not cover clinical aspects {however, seldom comments are made only if those issues are intimately / scientifically related & intermingle with ‘statistical aspects’ of the study}. Agreed that ‘statistical methods’ are used as just tools here, however, they are vital part of methodology [and so should be given due importance]. I look at the manuscript in/with statistical view point, other reviewer(s) look(s) at it with different angle so that in totality the review is very comprehensive. However, there should be efforts from authors side to improve (may be by taking clues from reviewer’s comments). Therefore, please do not limit the revision only (with respect) to comments made here.**

COMMENTS: Overall though there are not many flaws in the manuscript, I have few concerns. First and foremost {very serious} is about the ‘sample Size’ section. You stated that “due to the lack of suitable research to determine effect size, the sample size calculation was performed ---” which may be true. But even for medium effect size, according to table-2 on page 158 of Cohen’s paper “A power primer” in Psychological Bulletin, 1992, vol.:112, pp 155-159 [which is a sort of summary of the excellent book by Jacob Cohen titled ‘Statistical power analysis for the behavioral sciences’, Academic Press, 1977, New York] even for medium effect size you need n=64 per group (type-I error=0.05, power=80%). Therefore, what is you stated [“the sample size was performed considering a parallel group design, with no weighted patient preference, power of 0.81, type I error rate of 0.05, means method of analysis, and a loss up to 20% over time, a total of 36 participants will be required for the study (18 per group)] then implies that there is certainly some very serious error in your calculations. Kindly check (and modify the protocol manuscript accordingly).

Note that ‘How the required minimum sample size for this study was determined is nevertheless a very-very important question [one of the important items in CONSORT checklist, item 7a] for any type of study (clinical trial or else) which needs to be discussed in adequate details {including assumptions made at the time of estimation, power of the study, software used, etc.}’. Moreover, in ‘Strengths and Weakenesses of the Study’ section you say “The sample size was calculated to provide the appropriate statistical power to detect precise between-group differences for the primary outcomes”. I very doubtful about this statement. Please re-check. Otherwise, this small sample size is acceptable only for ‘pilot’ study (but if considered as pilot study, ‘pilot’ word should appear in title).

I even doubt if just saying that (refer to ‘Methods and Analysis - Study Design’ section) that “The results will be further reported following the Consolidated Standards of Reporting Trials (CONSORT)” is sufficient? I guess, not (because the important phrase ‘CONSORT’ appears only once in this manuscript). It is well-known that while reporting [findings from and even planning] ‘Clinical Trial’ one should follow CONSORT guidelines [since your article type is ‘Clinical Trial’, you are supposed to cover important CONSORT items in the report or even in the ‘Protocol’].

In ‘Data Analysis’ section since you stated that “Differences between groups will be calculated using unpaired t tests”, please note that, [especially because “the sample size calculation was performed applying a probabilistic model for data for trials from our primary outcome, the Goal Attainment Scaling (GAS) endpoint”], please note that though the measures/tools used are appropriate, most of them yield data that are in [at the most] ‘ordinal’ level of measurement [and not in ratio level of measurement for sure {as the score two times higher does not indicate presence of that parameter/phenomenon as double (for example, a Visual Analogue Scales VAS score or say ‘depression’ score)}]. Then application of suitable non-parametric test(s) is/are indicated/advisable [even if distribution may be ‘Gaussian’ (also called ‘normal’)]. Agreed that there is/are no non-parametric test(s)/technique(s) available to be used as alternative in all situation(s) [suitable / most desired/applicable], but should be used whenever/wherever they are available. This [‘ordinal’ level of measurement of data of primary outcome, the Goal Attainment Scaling (GAS) endpoint] also has implications in estimation of required sample size [it is sometimes suggested to inflate the sample size by 20-25% while dealing with ‘ordinal’ level of measurement data].

As pointed out in ‘important note’ above “This review pertains only to ‘statistical aspects’ of the study and so ‘clinical aspects’ should be assessed separately/independently [one should carefully consider/look at the clinical implications of the study]. In my opinion, to rescue this article (which is quite possible), some amount of re-vision (re-drafting) may be needed. However, please do not limit the revision only (with respect) to comments made here. The respected ‘Editor’ may consider accepting if found ‘clinical implications’ (of this study) valuable. ‘Major revision’ is recommended [assuming that the respected editor would to like to give chance of improvement of the manuscript as in my opinion, the study has potential].
