# Supplementary material for: Sports Stars Brazil in children with autism spectrum disorder: A feasibility randomized controlled trial protocol
Source: PLoS One. 2023 Nov 8;18(11):e0291488. doi: 10.1371/journal.pone.0291488 (PMC10631688; doi:10.1371/journal.pone.0291488)
Supplement: Attachment — Submitted filename: Response to Reviewers.docx [file pone.0291488.s011.docx]

**Review**

**Reviewer 2, Comment 1**

Reviewer #2: I am satisfied that my comments on the original version have been sufficiently addressed.

**Response:** Thanks again for your comments.

**Reviewer 3, Comment 1**

COMMENTS: It is very good that “considering all the queries raised and the reviewer’s suggestion on earlier draft, you decided to reformulate your paper as ‘pilot randomized controlled trial protocol’. However, in my knowledge the primary purpose of publishing a research protocol [of main study/trial] is a means to allow the academic community to evaluate whether subsequent analysis and results are in line with the investigators' initial objectives. Additionally, it informs the academic community on ongoing research and may avoid duplication of work. Agreed that making study protocols publicly available has the benefit of disseminating the most contemporary ideas with respect to study design and data analysis.

Nevertheless, the purpose/intention of ‘protocol for a pilot/feasibility study/trial is not known {at least to me and may be to many others as well}. Will these learned authors may please explain the purpose of protocol for this pilot study? Agreed that now this being a ‘pilot’ study in nature, sample size is not a big issue. However, [though many things are ignored (loosely looked at / evaluated)] in case of ‘pilot studies’, methodological issues need to be very rigorous followed {like in case of clinical trial, CONSORT guidelines are to be strictly observed/followed & you may know that a separate document on CONSORT for Pilot trials is available}.

**Response:**

We are in line with you. The publication of trial protocols, including pilot and feasibility trial protocols, has been advocated as an important strategy to improve transparency in the conduct and communication of pivotal trials and pilot/feasibility trials (Eldridge et al., 2016). A pilot study protocol is an important step in main study design and study rigor. There are several reasons for advocating the publication of pilot protocols as stated by CONSORT extension to pilot and feasibility:

- *The rationale of a pilot trial is to investigate areas of uncertainty about the future definitive RCT*
- *The primary aims and objectives of a pilot trial are therefore about feasibility, and this should guide the methodology used in the pilot trial*
- *Assessments or measurements to address each pilot trial objective should be the focus of data collection and analysis. This might include outcome measures likely to be used in the definitive trial but, equally, it might not*
- *Since the aim of a pilot trial is to assess the feasibility of proceeding to the future*

*definitive RCT, a decision process about how to proceed needs to be built into the*

*design of the pilot trial. This might involve formal progression criteria to decide*

*whether to proceed, to proceed with amendments, or not to proceed*

- *Methods used to address each pilot trial objective can be qualitative or quantitative. A mixed methods approach could result in both types of data being reported within the same paper. Equally, a process evaluation or other qualitative study can be done alongside a pilot trial and reported separately in more detail*
- *The number of participants in a pilot study should be based on the feasibility objectives and some rationale should be given*
- *Formal hypothesis testing for effectiveness (or efficacy) is not recommended. The aim of a pilot trial is not to assess effectiveness (or efficacy) and it will usually be underpowered to do this Research collaboration. Publishing the pilot protocol will enhance communication among groups of researchers interested in the same area and potential collaborations that may improve recruitment, expansion of the study and avoid doubling the research efforts*.

Considered all the purposes mentioned above of a feasibility study, we have better addressed and clarify in the new version of the manuscript our aims/objectives and methods to follow the CONSORT for pilot and feasibility recommendations. Please see the updates in the new version highlight in grey.

We also inserted a sentence into the discussion that highlights the benefits of the feasibility study. (Page 21, lines 437-439.

*This study presents a protocol of a feasibility RCT of Sports Stars Brazil in children with ASD compared to a waitlist control group. The main objective of the randomized pilot or feasibility testing is to assess the feasibility of drive the definitive future RCT.*

We also would like to emphasize that this protocol complies with the CONSORT guidelines for randomized pilot and feasibility trials. Furthermore, following the MacIntosh study (2020) [published previously on PLOS ONE], we would like to highlight that in the new version of the manuscript we divide our aims/objectives/methods into two following the Thabane et al (2010) framework, as following:

(Page 6, lines 110-121).

*This paper presents a feasibility Randomized clinical trial (RCT) protocol aiming to assess the feasibility of the Sports Stars Brazil intervention for children with ASD as articulated by Thabane et al (2010) framework. This framework encompasses that the aim of feasibility study might be linked to one or more of the following four classifications: process, resources, management and scientific. In this study, we will focus on two feasibility classifications*

*First, we will assess the process of feasibility that will determine the ability to enroll participants, the assessments completion rates, as well as adverse effects, satisfaction and adherence. To this purpose, a priori success criteria will be established when appropriated. Second, we will evaluated the scientific feasibility for estimating the effect size and variance of nine outcomes aligned with the fPRCs components.*

**Reviewer 3, Comment 2**

Though for most of the ‘Comments’ made earlier by me, the ‘Response’ is same, namely: We acknowledge that the statistical analysis and sample size calculation were the weaknesses of our study, as stated by the editor and all the three reviewers. Thus, considering all the queries raised and the reviewer’s suggestion, we decided to reformulate our paper, where a pilot RCT design have been reported accordingly throughout the updated manuscript. Consequently, we have also updated the statistical analysis to follow the new aims and purposes of the study. Pages 17-18, lines 363 to 371: “The sample size calculation was conducted using four main characteristics: significance level, power, an estimate of group difference and standard deviation.67 As there are no published studies on the effectiveness of Sports Stars intervention in children with ASD, to the ability to estimate group differences and variances is limited. It is known that one of the main goals of a pilot study is to give information on sample size for a future RCT.68 Therefore, while no sample size calculation is necessary in a pilot trial, 15-20 subjects by group are suggested to estimate effects from pilot studies and determine if enrollment is sufficient to progress to a full RCT.69 Our aim is to enroll 18 participants per group as the minimum sample size.

Note the following: As you said, “it is known that one of the main goals of a pilot study is to give information on sample size for a future RCT” by quoting reference number 68 is 100% true. Therefore, while no sample size calculation is necessary in a pilot trial is also agreed. Further you said “15-20 subjects by group are suggested to estimate effects from pilot studies” is not agreed because “According to document ‘CONSORT for Pilot trial’ - “Formal hypothesis testing for effectiveness (or efficacy) is not recommended. The aim of a pilot trial is not to assess effectiveness (or efficacy) and it will usually be underpowered to do this””, therefore please check the reference quoted w.r.t. this statement (reference 70).

Therefore, I do not have any specific recommendation [though only as system requirement I choose major revision]. Let the respected editor decide the future course. However, I request you to kindly note that I do not wish to re-review this paper/article again (let authors do any changes on accepting earlier & present comments).

**Response:** Thanks for your comments. We are in line with you in the new version of the manuscript we have better clarify our aims/purposes/methods to follow the CONSORT for pilot and feasibility study. The sample size section was rewritten, as following (Page 18, lines 382-387):

*This study is designed to investigate the feasibility of conducting a future RCT to evaluate the effectiveness of Sports Stars and to build decision-making processes to guide the execution of this larger study, particularly concerning satisfaction, adherence. Despite of no sample size calculation is necessary in a feasibility trial, 15-20 subjects by group are suggested to determine if enrollment is sufficient to progress to a full RCT. Our aim is to identify 36 potential participants to reach our target sample (18 per group).*

**Reviewer 4, Comment 1**

In the abstract- “Methods” section- better to write “This study will be conducted with 36 participants with ASD aged 6 to 12 years old”.

**Response:** Modified as suggested.

**Reviewer 4, Comment 2**

Please expand the abbreviations at the first time they are being mentioned.

**Response:** We appreciate your comment, abbreviations have been expanded.

**Reviewer 4, Comment 3**

I am not comfortable with the conclusions mentioned in the abstract “The results from this pilot study will inform if a full clinical trial is warranted and identify any necessary modifications required to the protocol”- I am not clear on what nature of the findings from the pilot will decide if a full clinical trial is required or not. I feel that a pilot trial is more appropriate to see what planning and preparations are required to conduct a full clinical trial. These could include barriers to acceptance of the interventions, the required sample size, feasibility of delivering the intervention etc.

**Response:** We appreciate your comment. The conclusion of the abstract has been reformulated as suggested (page 3, lines 29-31).

*The results of this feasibility study will inform which components are critical to planning and preparing a future RCT study, aiming to ensure that the RCT will be feasible, rigorous and justifiable.*

**Reviewer 4, Comment 4**

The inclusion criteria states that children aged 6-12 years will be eligible for inclusion. Any reason for such a broad age range? The effect of intervention being tested may vary depending on the age at which it is being initiated. It would be better if the authors could plan to have an age-stratified analysis, in addition to the overall analysis.

**Response:** Thank you for your comment and your suggestion. The original Sports Stars Protocol was designed for children aged 6 to 12 years with Cerebral Palsy, demonstrating positive results for all age groups (Clutterbuck, Auld, & Johnston, 2022). As stated by the other reviewers, it was necessary to reformulate the purposes of our feasibility study, since its main purposes its not to estimate effects and differences between groups. Thus, the new version of the manuscript is focusing on the feasibility aspects. The results of this pilot study will inform which components are critical to planning and preparing a future RCT study, aiming to ensure that the RCT will be feasible, rigorous and justifiable. In the future, using the full RCT data we can extrapolate the data, using other statistical analysis, to try to identify any age difference, etc.

Clutterbuck, G. L., Auld, M. L., & Johnston, L. M. (2022). SPORTS STARS: a practitioner-led, peer-group sports intervention for ambulant, school-aged children with cerebral palsy. Parent and physiotherapist perspectives. Disability and rehabilitation, 44(6), 956-965.

**Reviewer 4, Comment 5**

The exclusion criteria states that participants with cognitive, behavioral, or clinical limitations will be excluded. Will the investigators assess the child for these limitation at the time of screening for the study OR they will go by documented evidence?

**Response:** No specific instruments will be applied to assess these limitations, only subjective assessment during screening or parent reporting. This limitation has been included in the Discussion section. Page 21, lines 455-458.

*Finally, no specific instrument will be applied to assess possible cognitive alterations of the participants. Only subjective assessment of cognitive functioning will be performed during screening or through parents' reports.*

**Reviewer 4, Comment 6**

Regarding the consenting process- will the children or their caregivers (e.g., parents) provide informed consent? I worry that some younger children may not be able to understand the trial and what is expected from them better. Further, there would be children aged under 7 years and for them “assent” may be required. It would be good if the authors could provide more clarity on this.

**Response:** Parents and children signed the free and informed consent form and the free and informed assent form, respectively (This information can be found on page 20, lines 417 to 419). The term of assent has a language aimed at the child population, including numerous illustrations that facilitate the understanding of children. In addition, a researcher was available to assist in reading the terms and clarify any doubts the parents or children might have.

**Reviewer 4, Comment 7**

Please expand on what will the control group children receive (Nothing or the standard/routine care?). If it’s the routine care, then please elaborate on what is being currently provided under routine care.

**Response:** We appreciate your comment and agree that the information is not clear, and therefore, have been reformulated see page 16, lines 344-352).

*Participants in the control group will receive their standard care, including maintaining existing occupational therapy and/or physical therapy intervention programs. Children with ASD in Brazil are expected to receive 1 to 2 sessions of occupational therapy (but not physical therapy) per week in public or private clinics Both interventions provide individualized treatment plans tailored to the needs of each child. Generally speaking, physical therapy provides a general exercise program that involves gross motor training, muscle strengthening, and balance and coordination training. Occupational therapy usually involves sensory integration, neuropsychomotor development and participation in occupations (activities of daily living and education)*

References:

Eldridge, S. M., Chan, C. L., Campbell, M. J., Bond, C. M., Hopewell, S., Thabane, L., & Lancaster, G. A. (2016). CONSORT 2010 statement: extension to randomised pilot and feasibility trials. bmj, 355.

MacIntosh, A., Desailly, E., Vignais, N., Vigneron, V., & Biddiss, E. (2020). A biofeedback-enhanced therapeutic exercise video game intervention for young people with cerebral palsy: A randomized single-case experimental design feasibility study. PLoS One, 15(6), e0234767.

Thabane L, Ma J, Chu R, Cheng J, Ismaila A, Rios LP, et al. A tutorial on pilot studies: the what, why and how. BMC Med Res Methodol. 2010;10: 1. pmid:20053272
